# Somatic structural rearrangements in genetically engineered mouse mammary tumors

**DOI:** 10.1186/gb-2010-11-10-r100

**Published:** 2010-10-13

**Authors:** Ignacio Varela, Christiaan Klijn, Phillip J Stephens, Laura J Mudie, Lucy Stebbings, Danushka Galappaththige, Hanneke van der Gulden, Eva Schut, Sjoerd Klarenbeek, Peter J Campbell, Lodewyk FA Wessels, Michael R Stratton, Jos Jonkers, P Andrew Futreal, David J Adams

**Affiliations:** 1 Wellcome Trust Sanger Institute, Hinxton, Cambridge CB10 1SA, UK; 2 Netherlands Cancer Institute, Division of Molecular Biology, Plesmanlaan 121, 1066CX Amsterdam, The Netherlands; 3 Delft University of Technology, Delft Bioinformatics Lab., PO Box 5031. 2600 GA, Delft, The Netherlands; 4 Institute of Cancer Research, 15 Cotswold Road, Belmont, Sutton, Surrey, SM2 5NG, UK

## Abstract

**Background:**

Here we present the first paired-end sequencing of tumors from genetically engineered mouse models of cancer to determine how faithfully these models recapitulate the landscape of somatic rearrangements found in human tumors. These were models of *Trp53*-mutated breast cancer, *Brca1*- and *Brca2*-associated hereditary breast cancer, and E-cadherin (*Cdh1*) mutated lobular breast cancer.

**Results:**

We show that although *Brca1- *and *Brca2-*deficient mouse mammary tumors have a defect in the homologous recombination pathway, there is no apparent difference in the type or frequency of somatic rearrangements found in these cancers when compared to other mouse mammary cancers, and tumors from all genetic backgrounds showed evidence of microhomology-mediated repair and non-homologous end-joining processes. Importantly, mouse mammary tumors were found to carry fewer structural rearrangements than human mammary cancers and expressed in-frame fusion genes. Like the fusion genes found in human mammary tumors, these were not recurrent. One mouse tumor was found to contain an internal deletion of exons of the *Lrp1b *gene, which led to a smaller in-frame transcript. We found internal in-frame deletions in the human ortholog of this gene in a significant number (4.2%) of human cancer cell lines.

**Conclusions:**

Paired-end sequencing of mouse mammary tumors revealed that they display significant heterogeneity in their profiles of somatic rearrangement but, importantly, fewer rearrangements than cognate human mammary tumors, probably because these cancers have been induced by strong driver mutations engineered into the mouse genome. Both human and mouse mammary cancers carry expressed fusion genes and conserved homozygous deletions.

## Background

Cancers form in humans as a result of the accumulation of mutations that co-operate together in subversion of growth control and the cell death signals that would normally result in apoptosis. Somatic mutations in cancer genomes can be classified as those that contribute to the evolution of the cancer, so-called 'driver mutations', and 'passenger mutations' that can be used to reveal the signature of the underlying mutagenic process, but do not contribute to tumorigenesis. Generally, passenger mutations are thought to substantially outnumber driver mutations, meaning that functional validation is generally important to distinguish between these types of mutations. This complexity has led to the development of genetically engineered mouse models (GEMMs) that aim to faithfully recreate features of human cancers and in so doing create a platform for assessing the causality of candidate cancer genes [1]. Recently, we showed that there is a significant overlap in the cancer genes and pathways operative in human and mouse cancers [2]. Despite these similarities, however, there are fundamental differences in the ways cancers form in the two species. Unlike human tumors, cancers that form in mice are generally chromosomally stable and telomere dysfunction is rare [3]. Mouse cells also appear to be easier to transform than human cells, requiring fewer oncogenic events [4]. Nevertheless, there are many examples of GEMM tumor models that effectively recapitulate cardinal features of cognate human cancers [1], suggesting that basic features of many tumor suppressor networks, cell cycle checkpoints, and apoptotic pathways have been conserved through evolution.

Pioneering studies performed over 30 years ago showed that retroviral insertional mutagenesis could be used to discover cancer genes in the mouse, and *c-Myc*, *EviI *and *Bcl11a/b *are just a few genes discovered in this way [5]. More recently, transposon-mediated mutagenesis has been employed for cancer gene discovery in the mouse [6,7]. Unlike the analysis of human tumors, genomic analysis of mouse cancers is an approach that has been less widely exploited owing mainly to a lack of tools. Despite this, screening for DNA aberrations in GEMM tumors has lead to the discovery of several important cancer driver genes that have subsequently been shown to play a role in human cancer [8,9]. Until now, analysis of structural DNA rearrangements in mouse tumors has mainly relied on inferred breakpoint analysis based on copy number changes gleaned from array-based comparative genomic hybridization (aCGH) [10]. The major disadvantages of this technique include the above-base pair resolution, the lack of specific information as to how breakpoints relate to one another, and the techniques' inability to detect rearrangements that are copy number neutral. Paired-end massively parallel sequencing (PE-MPS) can be used to overcome these inherent shortcomings, as this technique allows all sequence rearrangements to be identified at base-pair resolution, including copy number neutral changes such as inversions and translocations.

We recently used PE-MPS to find structural rearrangements in 24 human breast cancers [11], a malignant melanoma [12] and a lung cancer [13]. PE-MPS has also been applied to the analysis of acute myeloid leukemias, a non-small cell lung cancer, and breast cancers by others [14-18]. An important limitation of human cancer genome sequencing is that the identification of driver mutations is complicated by the intrinsic heterogeneity in the genetic background of human populations, and therefore in the profile of somatic mutations that may arise. Analysis of cancers arising in inbred mouse strains, which have a defined genetic make-up, therefore potentially facilitates the identification of driver mutations. Moreover, since mice can be engineered to carry known cancer causing mutations that will act as potent promoters of tumor formation, it might be expected that the ratio of driver to passenger mutations will be significantly enriched compared to human tumors. Finally, experimental tumor models may permit the identification of genetic aberrations associated with specific traits such as tumor progression, metastasis and therapy resistance, which cannot be readily assessed in humans. Together, these advantages make GEMMs an ideal system to screen for genetic aberrations associated with cancer.

In this study we used PE-MPS to analyze the genomes of eight mouse mammary tumors derived from four different GEMMs of breast cancer: *K14cre;Brca1*^*flox/flox*^*;Trp53*^*flox/flox*^, *K14cre;Brca2*^*flox/flox*^*;Trp53*^*flox/flox*^, *K14cre;Cdh1*^*flox/flox*^*;Trp53*^*flox/flox *^and *K14cre;Trp53*^*flox/flox *^[19-21] (Table 1). In these GEMMs, epithelium-specific expression of Cre recombinase induces mammary tumors driven by deletion of *Trp53 *alone, or in combination with deletion of *Brca1*, *Brca2 *or *Cdh1 *(encoding E-cadherin). The *K14cre;Brca1*^*flox/flox*^*;Trp53*^*flox/flox *^and *K14cre;Brca2*^*flox/flox*^*;Trp53*^*flox/flox *^mice develop mammary tumors with a defect in homologous recombination (HR) due to genetic knockout of *Brca1 *or *Brca2*, respectively [22-24]. In contrast, tumors arising in *K14cre;Cdh1*^*flox/flox*^*;Trp53*^*flox/flox *^and *K14cre;Trp53*^*flox/flox *^mice are HR-proficient, assuming that they have not gained a functional mutation in a member of the HR repair machinery during their evolution. Our primary aim was to characterize somatic rearrangements in these different mouse tumor models to see whether they resemble rearrangements found in human breast cancers, while our secondary aim was to identify features of the somatic rearrangements that may distinguish between HR-proficient and HR-deficient tumors. Discovery of the genomic features that discriminate between these two functionally different types of tumors may facilitate the identification of patients with HR-deficient tumors, who can be effectively treated with platinum drugs [22] or poly(ADP-ribose) polymerase (PARP) inhibitors [23].

**Table 1:** Overview of mouse tumors analyzed

Number of samples	Genotype	Latency (days)	Homologous repair	p53 status	Reference	Identifiers	Histology	Tumor (%)
2	*K14cre;Trp53*^*flox/flox*^	504	Proficient	Heterozygous	Jonkers *et al. *2001 [20]	PD3685a	Mesenchymal	75
		386				PD3686a	Mesenchymal	50
2	*K14cre;Cdh1*^*flox/flox*^*;Trp53*^*flox/flox*^	449	Proficient	Null	Derksen *et al. *2006 [19]	PD3679a	mILC^a^	85
		328				PD3680a	mILC^a^/Carcinoma	85
2	*K14cre;Brca1*^*flox/flox*^*;Trp53*^*flox/flox*^	99	Deficient	Null	Liu *et al. *2007 [21]	PD3681a	Carcinoma	95
		247				PD3682a	Carcinoma	95
2	*K14cre;Brca2*^*flox/flox*^*;Trp53*^*flox/flox*^	144	Deficient	Null	Jonkers *et al. *2001 [20]	PD3683a	Carcinoma	95
		227				PD3684a	Carcinoma	95

^a^Mouse invasive lobular carcinoma.

## Results

### Mouse models used in this study and tumor sequencing

We sought to determine whether the functional abrogation of HR would lead to differences in DNA structural rearrangements in mouse models of breast cancer. To test this we used PE-MPS to analyze four HR-deficient mouse mammary tumors derived from *K14cre;Brca1*^*flox/flox*^*;Trp53*^*flox/flox *^and *K14cre;Brca2*^*flox/flox*^*;Trp53*^*flox/flox *^conditional knock-out mice [19-21], and four tumors derived from *K14cre;Cdh1*^*flox/flox*^*;Trp53*^*flox/flox *^and two *K14cre;Trp53*^*flox/flox *^mice that do not carry engineered mutations in the HR machinery [19-21,24]. All tumors were genotyped to confirm homozygous deletion of all *flox *alleles, except for the *K14cre;Trp53*^*flox/flox *^tumors, which showed heterozygous loss of *Trp53 *as determined by Southern blot analysis (Additional file 1). We sequenced the remaining *Trp53 *allele in these tumors but were unable to identify any somatic mutations resulting in a loss of heterozygosity. The features of the samples used in this study are listed in Table 1 and in the Materials and methods.

**Figure 1:**
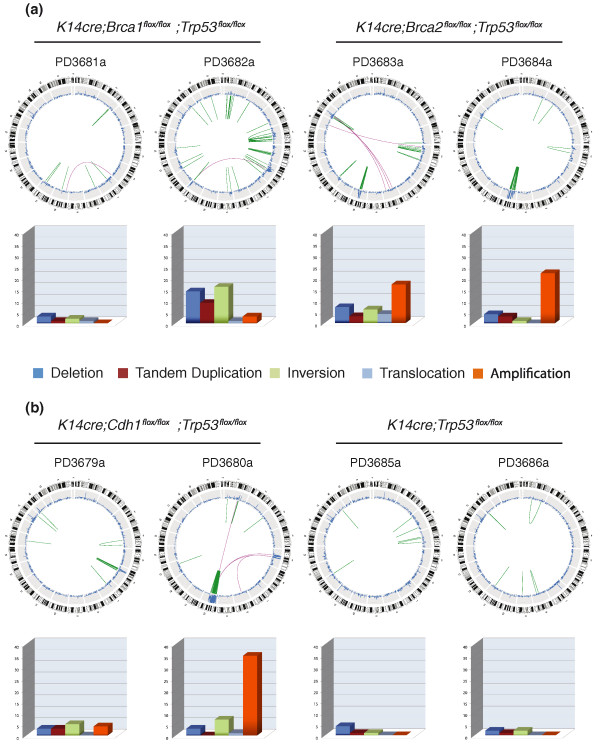
**Overview of somatic rearrangements in mouse mammary tumors**. **(a) **Homologous recombination deficient tumors. **(b) **Homologous recombination proficient tumors. Circos plots showing the genome-wide distribution of structural aberrations. An ideogram of the mouse genome is show in the outer ring. The blue line indicates changes in copy number as determined by read coverage density. Intra- (green) and inter-chromosomal (purple) rearrangements are shown by lines within the circle. The bar plots show the absolute number of rearrangements found per type. Dark blue denotes deletions, red denotes tandem duplications, green denotes inversions, light blue denotes interchromosomal rearrangements, and orange denotes amplifications.

We used the Illumina GAII platform at the Sanger Institute to obtain around 60 million paired-end 37-bp reads from each sample by sequencing Illumina libraries prepared using DNA fragmented to around 450 bp (Additional file 2). Paired-end sequencing resulted in an average of 7.5 × physical coverage of the mouse genome of each tumor. Discordantly mapped reads were flagged as those potentially marking structural rearrangements. We filtered these reads for the presence of long terminal repeats and short interspersed repetitive elements (SINEs) to reduce false positive variant calls. All candidate rearrangements spanned by at least two independent reads and larger than 10 kbp that passed these filters were validated using genomic PCR on the tumor sample and matched normal (spleen) DNA to confirm that the breakpoint was somatic. An overview of validated rearrangements is shown in Figure 1 and Additional file 3. Importantly, we detected the recombination event associated with Cre-mediated deletion of the *Brca1 *alelle (20 kb) in one tumor but we did not detect the deletions associated with recombination of the *Cdh1 *(14 kb), *Trp53 *(8 kb) or *Brca2 *(7 kb) alleles. As mentioned above, our analysis was designed to detect rearrangements >10 kb, meaning that we would not expect to retrieve Cre-mediated *Brca2 *or *p53 *rearrangements, although examination of read data over the *Brca2 *and *p53 *loci provided support for the presence of these deletions. The fact that we were unable to detect Cre-mediated deletion of *Cdh1 *in PD3679a or PD3680a or *Brca1 *in PD3682a suggests that some rearrangements are not detectable by our approach, possibly because of the sequence depth we generated across these tumors, the repeat structure of the mouse genome at these loci, or the filtering we performed prior to analysis. In our previous analysis of human breast cancers we estimated that we were able to recover around 50% of the structural rearrangements found in a cancer genome. A similar figure to that reported by others [11,17].

### Somatic rearrangements in mouse models for breast cancer

In general, tumors with homozygous deletion of *Trp53 *(PD3681a, PD3682a, PD3683a, PD3684a, PD3679a, PD3680a) showed a larger number of rearrangements than the *Trp53 *heterozygous tumors (PD3685a, PD3686a) (Figure 1; *P *< 0.02, two tailed *t*-test). Two *K14cre;Brca2*^*flox/flox*^*;Trp53*^*flox/flox *^tumors and one *K14cre;Cdh1*^*flox/flox*^*;Trp53*^*flox/flox *^tumor were found to harbor large amplicons within the same region of chromosome 10. Although these amplicons contained many rearrangements, we could not identify any recurrent somatic event. No specific type of rearrangement was found to discriminate between the different genotypes of the tumors sequenced (Figure 1), and as seen in human breast tumors, mouse mammary tumors showed significant heterogeneity in their genomic profiles. Importantly, none of the mouse mammary tumors showed the tandem duplication phenotype that we have observed in human *BRCA1*-mutated and triple-negative breast tumors (that is, tumors that do not express ERBB2, and estrogen and progesterone receptors) [11].

### Microhomology and non-template DNA at rearrangement breakpoints

**Figure 2:**
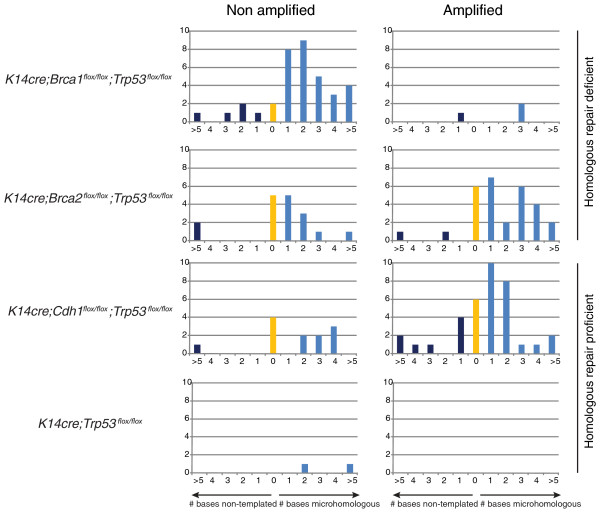
**Overview of breakpoint sequence homology and non-templated DNA sequence per genotype**. The bar plots show the amount of microhomology and non-templated DNA found binned by sequence length. The plots show aggregate values per genotype, separated into amplification-associated and non-amplification associated rearrangements.

We used conventional capillary sequencing to determine the exact DNA sequence at the breakpoints of the rearrangements (Figure 2). All tumors showed evidence of sequence microhomology at the breakpoints, a hallmark of non-homologous end-joining (NHEJ) or microhomology-mediated repair (MHMR) [25]. When specifically examining the results for the non-amplicon related rearrangements, the *Brca1*-mutated tumors showed a remarkable amount of microhomology at the breakpoints, which indicates a potential preference for NHEJ or MHMR by tumors of this genotype (Figure 2). Remarkably, the *Brca2*-mutated tumors with a functionally similar defect in HR showed no such inclination towards microhomology.

### Generation of fusion genes and their expression

**Figure 3:**
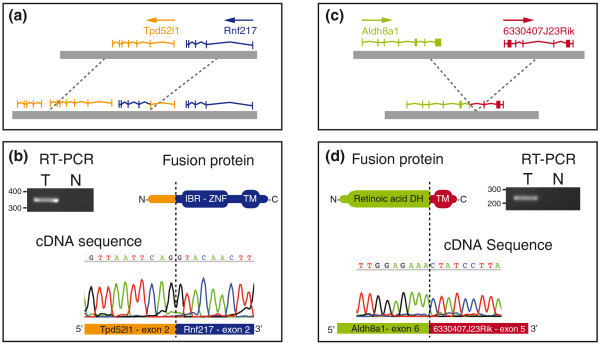
**Expressed fusion genes found in tumor PD3680a**. **(a) **Schematic representation of the fusion of genes *Tpd52l1 *and *Rnf217 *by tandem duplication. **(b) **RT-PCR product of RNA between exon 2 of *Tpd52l1 *and exon 2 of *Rnf217*. Sequence trace of the RT-PCR product confirming the fusion at the RNA level. A schematic representation of the putative fusion gene product. IBR-ZNF, in between ring fingers-zinc finger domain PF01485; TM, transmembrane domain - predicted by TMHMM. **(c) **Schematic representation of the fusion of genes *Aldh8a1 *and *6330407J23Rik *by deletion. **(d) **RT-PCR product of RNA between exon 6 of *Aldh8a1 *and exon 5 of *6330407J23Rik*. Sequence trace of the RT-PCR product confirming the fusion at the RNA level. A schematic representation of the putative fusion gene product. Retinoic acid DH, aldehyde dehydrogenase PF00171; TM, transmembrane domain - predicted by TMHMM.

Validation of the breakpoints led to the prediction of three in-frame fusion genes, as well as two in-frame internally rearranged genes. To test whether these predicted fusions were transcribed, we extracted RNA from the sequenced tumors and applied RT-PCR using three distinct primer pairs spanning the predicted fusion boundaries in the transcript. Two fusion genes originating from the chromosome 10 amplicon in tumor PD3680a were found to be expressed (Figure 3). We confirmed these fusions at the RNA level by capillary sequencing of RT-PCR products (Figure 3). The fusion between the genes *Rnf217 *and *Tpd52l1 *is the result of a 200-kb tandem duplication (Figure 3a). This fusion transcript encodes a protein in which the first two exons of *Tpd52l1*, which does not code for any known protein domains, and all exons of *Rnf217*, which contains an abrogated zinc finger domain (IBR-ZNF) with a carboxy-terminal transmembrane domain, are joined together. The largest part of the fusion protein is derived from *Rnf217*, the function of which is unknown. The fusion between *Aldh8a1 *and *6330407J23Rik *is the result of an 8-Mb deletion. The fusion transcript encodes a protein that contains most of the retinoic acid dehydrogenase domain of *Aldh8a1 *fused to a carboxy-terminal transmembrane domain encoded by the last four exons of *6330407J23Rik*.

Using RT-PCR we screened RNA from an additional 19 *K14cre;Brca2*^*flox/flox*^*;Trp53*^*flox/flox *^mouse mammary tumors, all of which carried the chromosome 10 amplification, but we were unable to find any evidence for expression of either fusion gene in these tumors. We conclude that, similar to human breast cancers, mouse mammary tumors contain non-recurrent in-frame fusion genes.

### Internally rearranged genes

**Figure 4:**
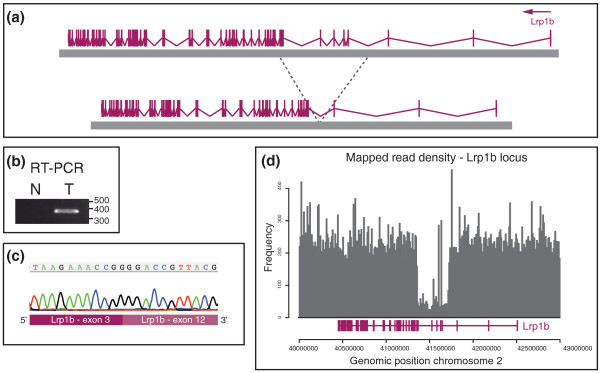
**The internally rearranged gene *Lrp1b*/*LRP1b***. **(a) **Schematic representation of the *Lrp1b *internal deletion in PD3682a. **(b) **RT-PCR product of RNA between exon 3 and exon 12 of *Lrp1b*. **(c) **Sequence trace of the RT-PCR product confirms the fusion between exon 3 and 12. **(d) **Read coverage density confirms the deletion of exons 4 to 11.

Of the two predicted in-frame internally rearranged genes, one, *Lrp1b*, was found to be expressed by RT-PCR (Figure 4B). *Lrp1b *encodes a member of the low density lipoprotein (LDL) receptor gene family. We confirmed the internal deletion of *Lrp1b *exons 4 to 11 by capillary sequencing of the RT-PCR product (Figure 4C). The reduced number of reads mapping to the *Lrp1b *locus further confirmed the intragenic deletion of this gene (Figure 4D). The read density at the *Lrp1b *locus was similar to the read density at the homozygously deleted *Trp53 *locus, suggesting homozygous deletion of *Lrp1b*.

### Internal deletions of human *LRP1B*

**Figure 5:**
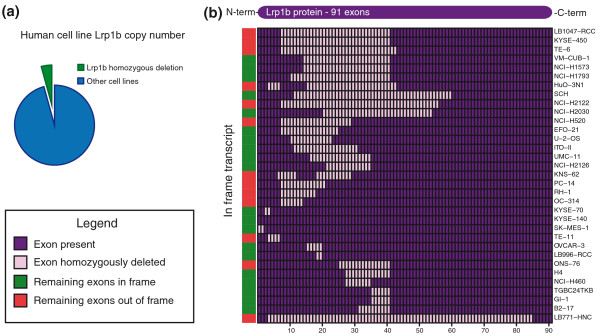
**Human cell lines frequently carry homozygous deletions of the *LRP1B *gene**. **(a) **Pie chart showing the proportion of the 770 cell lines containing homozygous deletions in the *LRP1B *gene. **(b) **Heatmap showing which exons of the *LRP1B *gene have been homozygously deleted. The x-axis shows the exons in transcriptional order. The y-axis shows the different cell lines, clustered using hierarchical Euclidean clustering on the deletion patterns. The color bar along the y-axis shows whether an in-frame transcript would remain if these exons have been deleted.

To determine whether the internal deletion of exons in the *Lrp1b *gene is relevant to human cancer, we examined 770 human cancer cell lines for which we had previously generated high-resolution aCGH (Affymetrix SNP6) data [26]. We analyzed these cell lines through the CONAN copy number analysis algorithm [26]. We then used the PICNIC copy number algorithm [27] to identify tumors carrying homozygous deletions of exons of *LRP1B*. Out of the 770 cell lines, 33 (4.2%) harbored internal homozygous deletions of *LRP1B *(Figure 5). Importantly, deletion of *LRP1B *did not correlate with *P53 *status (*P *= 0.096). Thirty-two of the *LRP1b *deletions removed one or more exons and intragenic deletions of *LRP1b *were predicted to generate in-frame transcripts in 20 of them. To follow up on this analysis, we analyzed a collection of 102 sporadic breast cancers [28] but were unable to identify internal deletions of *LRP1b*, suggesting that it is a relatively rare event in sporadic breast cancer (Additional file 4), or that it is associated with a subtype of disease not represented by this dataset.

## Discussion

Massively parallel sequencing of tumors from mouse models of human cancer has several advantages. It permits in-depth analysis of the evolution of cancer genomes during tumor development, progression and metastasis, and during therapeutic interventions, including development of therapy resistance. Here, we show that PE-MPS provides an effective means to generate comprehensive catalogues of somatic structural rearrangements in tumors from GEMMs of human breast cancer. Compared to our recent study of somatic rearrangements in human breast cancers, the absolute number of rearrangements we have observed in mouse mammary cancers is lower [11]. This might be due to the nature of the models studied where we have engineered into the mouse genome one or more known tumor-initiating lesions, thus reducing the requirement for several tumor suppressors and oncogenes to be mutated. It may also be due to the fact that these mice develop tumors very quickly, after about 200 days (*K14cre;Brca1*^*flox/flox*^*;Trp53*^*flox/flox *^and *K14cre;Brca2*^*flox/flox*^*;Trp53*^*flox/flox *^and *K14cre;Cdh1*^*flox/flox*^*;Trp53*^*flox/flox *^models) [19-21] or around 400 days (*K14cre;Trp53*^*flox/flox *^model) [20] and therefore there is less opportunity for a substantial passenger mutation load to accumulate. We previously found that human primary breast tumors and breast cancer cell lines carry tandem duplications [11]. In contrast, we have not been able to identify these rearrangements in any of the mouse tumors we sequenced. The tandem duplication phenotype in human tumors might be associated with a specific breast cancer subtype that is not fully recapitulated by the mouse models we studied, or these rearrangements may be associated with the slow kinetics of human breast cancer development, or possibly of more fundamental differences between the mouse and human genomes. The differences in the structure of the mouse and human mammary cancer genome may also reflect fundamental differences in the biology of mouse and human cells [4]. Mouse cells, for example, do not undergo telomere erosion and will readily undergo immortilization *in vitro*, whereas human cells will enter replicative senescence under the same conditions. Based on this and other observations, it has been suggested that fewer mutations are required to transform or immortalize mouse cells and the fact that structures such as telomeres play an important role in how the genome is rearranged in cancer makes it plausible to suggest that mouse cancer genomes may show different rearrangements to their human counterparts.

The presence of microhomology sequences at the breakpoints of chromosomal rearrangements is a hallmark of NHEJ or MHMR [25]. We only found a clear preference for microhomologous sequences in the non-amplified rearrangements in the *K14cre;Brca1*^*flox/flox*^*;Trp53*^*flox/flox *^tumors. This could hint towards a dependence of *Brca1*-deficient tumors on NHEJ. It should be noted, however, that sample numbers are too low to draw any statistical conclusions from this observation. We did not find a clear preference for 0-base microhomology in amplified rearrangements as reported for human breast cancer [11]. Despite the fact that we did not find compelling evidence for homologous recombination deficiency in the *Brca1*- and *Brca2*-deficient tumors, we have previously shown that tumors from these models are highly sensitive to the PARP inhibitor AZD2281 [24]. This may suggest that these tumors carry a significant load of other rearrangements possibly driven by defects in other repair mechanisms.

We observed two expressed fusion genes, both originating from a complex amplification on chromosome 10 in the same *K14cre;Brca2*^*flox/flox*^*;Trp53*^*flox/flox *^tumor (PD3680a). The possible function of these fusion transcripts and their relevance to cancer development are currently unknown. It is becoming increasingly apparent that fusion genes are present in a large number of epithelial tumors, but so far few have been shown to be recurrent. The amplification on chromosome 10 itself was found in three samples, yet the minimal amplicon is several mega-bases long, containing many genes. Sequencing a larger number of mouse tumors will be necessary to define the driver genes in this rearrangement.

Strikingly, the observed rate of homozygous deletions within *LRP1B *in human cancer cell lines is equivalent to or higher than known recessive tumor-suppressor genes such as *PTEN*, *RB1 *and *SMAD4 *in the same cell line dataset (Figure 5) [26]. It should be noted, however, that the *LRP1B *gene is large (around 2 Mb) so is potentially at higher risk of accumulating homozygous deletions. Moreover, the *LRP1B *locus is a known fragile site (*FRA2F*). This may indicate that deletions at this locus are sequence driven, rather than associated with tumorigenesis. In a recent study, however, analysis of human aCGH data revealed that the *LRP1B *locus (*FRA2F*) was the least sensitive fragile site in the aphidicolin fragility assay, and scored highest in a computational measure for homozygous deletion selection pressure [26]. Furthermore, *LRP1B *is not only a frequently deleted gene in tumors but it is also frequently point mutated in lung cancer, and its promoter is frequently methylated in several cancer types [29,30]. Analysis of expression array data available in Oncomine revealed that *LRP1b *is expressed in human breast cancer cells, although we were unable to detect recurrent deletions of *LRP1b *in a collection of sporadic primary human breast cancers, suggesting that deletion of this gene may be a relatively rare event (Additional file 4), or that it may be associated with a specific subtype of disease not represented by the collection we analyzed [31]. In support of a role for *LRP1b *in breast tumorigenesis, it was recently shown that deletion of *LRP1b *is associated with the evolution of MCF10A cells, which are an immortal mammary epithelial cell line, into malignant tumors in a xenograft model of mammary cancer [31]. Thus, *LRP1b *may have cell or subtype-specific disease associated roles in mammary tumorigenesis. We recently developed two knockout mouse models of *Lrp1b *in which we disrupted internal exons of the gene revealing a critical role for this transmembrane receptor in embryonic development. These mice represent an invaluable tool for assessing the role of *Lrp1b *in tumorigenesis [32].

## Conclusions

In our study we present the first genome-wide screen for somatic structural rearrangements in genetically engineered mouse tumors using PE-MPS. We analyzed tumors of four genotypes of mouse mammary cancer, of which two were HR-proficient and two were HR-deficient. We could not find any features of the rearrangements found in these cancers that were specific for either the HR-proficient or HR-deficient tumor types, within the small collection of tumors we analyzed. For now, it appears as though NHEJ or MHMR processes are used as often in HR-proficient tumors as in HR-deficient tumors. As we previously reported for human mammary tumors, mouse mammary tumors showed evidence of microhomology and non-template DNA repair, and expressed fusion transcripts, which are a poorly understood feature of human epithelial tumors.

## Materials and methods

### Tumor collection

The mouse models of breast cancer used in this study have been described previously [19-21]. Mutant alleles in these models were generated in E14 embryonic stem cells (129P2/Ola) and transmitted onto an FVB/n background. Tumors were isolated from mice when they became palpable and were bisected with half the tumor being processed for histopathological analysis and the other half being processed for DNA extraction. Each tumor evolved in an independent animal. Tumor latency was as follows: PD3686a (386 days), PD3679a (449 days), PD3685a (509 days), PD3680a (328 days), PD3681a (99 days), PD3682a (247 days), PD3683a (144 days), PD3684a (227 days). Tumors were graded for stroma and necrosis. All of the tumors analyzed in this study were assessed to be composed of, on average, 85% tumor nuclei: PD3686a (50%), PD3679a (85%), PD3685a (75%), PD3680a (85%), PD3681a (95%), PD3682a (95%), PD3683a (95%), PD3684a (95%).

### Library construction and paired-end sequencing

Genomic libraries from eight mouse mammary cancers were generated using 5 μg of total genomic DNA. Briefly, 5 μg of genomic DNA was randomly fragmented to around 450 bp by focused acoustic shearing (Covaris Inc. Woburn, Massachusetts, USA). These fragments were electrophoresed on a 2% agarose gel and the 400- to 550-bp fraction was excised and extracted using the Qiagen (Crawley, West Sussex, UK) gel extraction kit (with gel dissolution in chaotropic buffer at room temperature to ensure recovery of (A+T)-rich sequences).

The size-fractionated DNA was end repaired using T4 DNA polymerase, Klenow polymerase and T4 polynucleotide kinase. The resulting blunt-ended fragments were A-tailed using a 3'-5' exonuclease-deficient Klenow fragment and ligated to Illumina paired-end adaptor oligonucleotides in a 'TA' ligation at room temperature for 15 minutes. The ligation mixture was electrophoresed on a 2% agarose gel and size-selected by removing a 2-mm horizontal slice of gel at approximately 600 bp using a sterile scalpel blade. DNA was extracted from the agarose as above. Ten nanograms of the resulting DNA was PCR-amplified for 18 cycles using 2 units of Phusion polymerase. PCR cleanup was performed using AMPure beads (Agencourt BioSciences Corporation Beverly, MA, USA) following the manufacturer's protocol. We prepared Genome Analyzer paired-end flow cells on the supplied Illumina cluster station and generated 37-bp paired-end sequence reads on the Illumina Genome Analyzer platform following the manufacturer's protocol. Images from the Genome Analyzer were processed using the manufacturer's software to generate FASTQ sequence files. These were aligned to the mouse genome (NCBI build 37) using the MAQ algorithm v.0.6.8. A detailed breakdown of the sequencing and mapping of the data for each tumor is provided in Additional file 2.

### Data submission

The sequence data generated as part of this project are available in the European Nucleotide Archive (ENA). The project accession is [ENA:ERP000258].

### Reads removed from structural variant analysis

Reads that failed to align in the expected orientation or distance apart were further evaluated using the SSAHA algorithm to remove mapping errors in repetitive regions of the genome. In addition, during the PCR enrichment step, multiple PCR products derived from the same genomic template can occasionally be sequenced. To remove these, reads where both ends mapped to identical genomic locations (plus or minus a single nucleotide) were considered PCR duplicates, and only the read pair with the highest mapping quality retained. Further, erroneous mapping of reads originating from DNA present in sequence gaps in NCBI build m37 assembly were removed by excluding the highly repetitive regions within 1 Mb of a centromeric or telomeric sequence gap. Additional read pairs, where both ends mapped to within less than 500 bp of one another, but in the incorrect orientation, were excluded from analysis, unless support for a putative rearrangement was indicated by additional read pairs. The majority of these singleton read pairs are likely to be artifacts resulting from either intramolecular rearrangements generated during library amplification or mispriming of the sequencing oligonucleotide within the bridge amplified cluster. Finally, read pairs where both ends mapped to within 500 bp of a previously identified germline structural variant were removed from further analysis, as these are likely to represent the same germline allele.

### Generation of genome-wide copy number plots

Generation of high-resolution copy number plots has been described previously [11,12,33]. Briefly, the mouse reference genome was divided into bins of approximately 15 kb of mappable sequence and high-quality, correctly mapping read pairs, with a MAQ alternative mapping quality ≥35, were assigned to their correct bin and plotted. A binary circular segmentation algorithm originally developed for genomic hybridization microarray data was applied to these raw plots to identify change points in copy number by iterative binary segmentation [34].

### PCR confirmation of putative rearrangements

The following criteria were used to determine which incorrectly mapping read pairs were evaluated by confirmatory PCR: 1, reads mapping ≥10 kb apart spanned by ≥2 read-independent read pairs (where at least one read pair had an alternative mapping quality ≥35); 2, reads mapping ≥10 kb apart spanned by 1 read pair (with an alternative mapping quality ≥35), with both ends mapping to within 100 kb of a change point in copy number identified by the segmentation algorithm; 3, reads mapping ≥600 bp apart spanned by ≥2 read-independent read pairs (where at least one read pair had an alternative mapping quality ≥35) with both ends mapping to within 100 kb of a change point in copy number identified by the segmentation algorithm; 4, selected read pairs mapping between 600 bp and 10 kb apart spanned by ≥2 independent read pairs (where at least one read pair had an alternative mapping quality ≥35). Primers were designed to span the possible breakpoint and to generate a maximum product size of 1 kb. PCR reactions were performed on tumor and normal genomic DNA for each set of primers at least twice, using the following thermocycling parameters: 95°C × 15 minutes (95°C × 30 s, 60°C × 30 s, 72°C × 30 s) for 30 cycles, 72°C × 10 minutes. Products giving a band were sequenced by conventional Sanger capillary methods and compared to the reference sequence to identify breakpoints. Somatically acquired rearrangements were defined as those generating a reproducible band in the tumor DNA with no band in the normal (spleen) DNA following PCR amplification, together with unambiguously mapping sequence data suggesting a rearrangement. To support the somatic origin of the rearrangements identified in this study, we compared our calls to known structural variants [35]. Importantly, >95% of our somatic variant calls did not map in the vicinity of previously described germline structural variants.

### Breakpoint analysis

All breakpoints defined to the base-pair level were used in the analysis of breakpoint sequence context, excluding shards and overlapping regions. Analysis was performed on all breakpoints together, and also on subsets divided into deletions, tandem duplications, amplicons, other intrachromosomal events, and all interchromosomal events. We extracted 10 bp and 100 bp on either side of the breakpoint sites for analysis.

### RT-PCR analysis of fusion transcripts

RNA was extracted from mouse mammary tumor samples using Trizol (Invitrogen, Paisley, Scotland, UK) and reverse transcribed using random hexamers. Three combinations of two forward and two reverse PCR primers were designed to span the fusion breakpoints. Primer sequences are shown in Additional file 5. We used 2 μl of the 1:20 cDNA dilution in the following PCR program: 2.5 min 95°, 35 cycles of (I) 30 s 95° (II) 30 s 58° (III) 50 s 72°, 5 minutes 72°C. If the PCR showed an amplification product, we employed capillary sequencing with both forward and reverse primers on the PCR product to confirm the sequence at the exon-exon boundaries and to determine if the fusion transcript was in-frame.

### Sequencing of *Trp53 *in PD3685a and PD3686a

Primers were design to amplify all exons of *Trp53 *(ENSMUST00000108658; CCDS36193). The PCR reactions and capillary sequencing were performed in duplicates on tumor and normal DNA samples following standard protocols. The resulting traces were aligned against the mouse reference genome (NCBIM37) using the BLAST algorithm. We obtained 90.2% sequence coverage of *Trp53 *in PD3685a and 90.6% sequence coverage of this gene in PD3686a. Traces were manually inspected to identify potential somatic mutations. Primer sequences are provided in Additional file 6.

### Analysis of Affymetrix SNP6 data from human cancer cell lines

We used the web-based analysis tool CONAN to determine which cell lines had a homozygous deletion in the *LRP1B *gene. We analyzed the PICNIC output for all cell lines flagged with homozygous deletions at the *LRP1B *locus. PICNIC assigns an absolute copy number score to SNP6 probes [26,27]. To determine the exact location of the homozygous deletions, we called all exons within a contiguous region of copy number call = 0 as homozygously deleted.

## Abbreviations

aCGH: array comparative genomic hybridization; bp: base pair; GEMM: genetically engineered mouse model; HR: homologous recombination; MHMR: microhomology-mediated repair; NHEJ: non-homologous end-joining; PARP: poly(ADP-ribose) polymerase; PE-MPS: paired end massive parallel sequencing.

## Authors' contributions

IV, CK, PJS, LJM, HvG and ES performed experiments. LS, DG, PJC, and LFAW performed data analysis. MRS, JJ, PAF and DJA designed the research. CK and DJA wrote the paper.

## Supplementary Material

Additional file 1 **A Southern blot hybridization showing the status of *Trp53 *(*p53*) in eight tumors sequenced as part of this study**. The PCR primers shown were used to generate a Southern blot probe that was hybridized with size-fractioned genomic DNA. The 'wt' band represents the wild-type allele. The 'del' band represents the recombined *Trp53 *allele. Click here for file

Additional file 2 **An overview of the sequencing and sequence read mapping metrics for the eight mouse mammary cancers sequenced as part of this study**. Click here for file

Additional file 3 **A list of somatic structural rearrangements found in eight mouse mammary cancers**. Each row represents a single somatically acquired rearrangement. The 'Sample' column represents the mammary cancer that was sequenced as part of this study in which the rearrangement was found. 'No. Reads' denotes the number of mapped reads spanning the rearrangement. 'Simplified Nomenclature' refers to the type of rearrangement. The 'Chr1' column represents the chromosome on which the rearrangement is resident. For intrachromosomal rearrangements the 'Chr2' column will denote the same chromosome as shown in the Chr1 column. For interchromosomal rearrangements Chr2 will carry a different chromosome. Position and strand represent the location of the rearrangement and the strand to which the sequence reads have mapped. The 'Size' column represents the distance between the mapped reads for an intrachromosomal rearrangement while the 'ReadName' column reports a single read that uniquely identifies the rearrangement. Click here for file

Additional file 4 **Copy number analysis of the *LRP1b *locus in 102 sporadic human breast cancers**. Click here for file

Additional file 5 **Primers used for fusion gene validation**. Click here for file

Additional file 6 **Primers used to sequence *Trp53***. Click here for file
