# Supplementary material for: Somatic structural rearrangements in genetically engineered mouse mammary tumors
Source: Genome Biol. 2010 Oct 13;11(10):R100. doi: 10.1186/gb-2010-11-10-r100 (PMC3218656; doi:10.1186/gb-2010-11-10-r100)
Supplement: Additional file 1 — A Southern blot hybridization showing the status of Trp53 (p53) in eight tumors sequenced as part of this study. The PCR primers shown were used to generate a Southern blot probe that was hybridized with size-fractioned genomic DNA. The 'wt' band represents the wild-type allele. The 'del' band represents the recombined Trp53 allele. [file gb-2010-11-10-r100-S1.PDF]

Additional File 1: *Trp53* Southern blot.

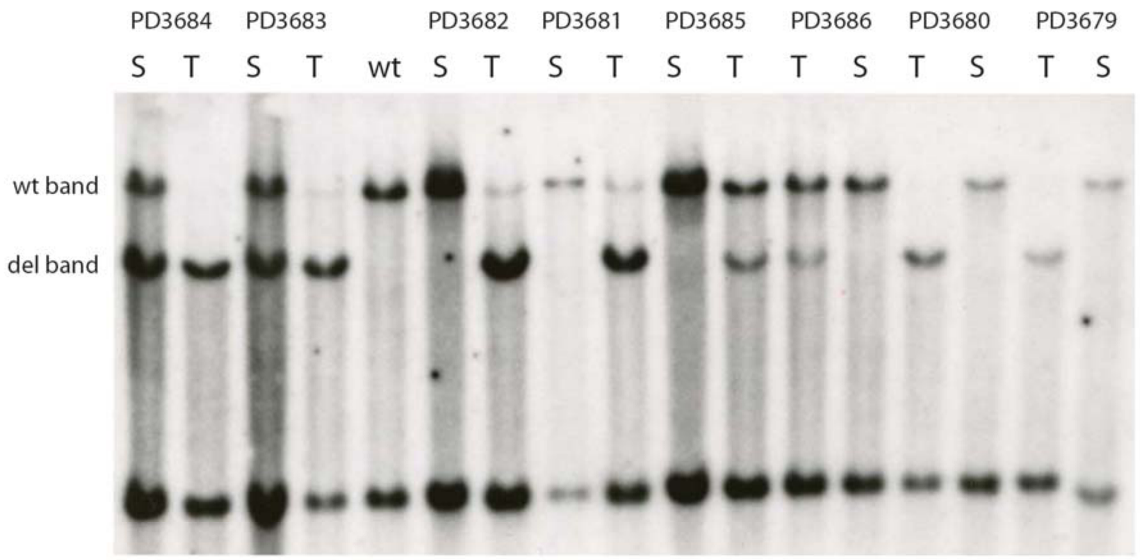

S - Spleen
T - Tumor
wt - Wild Type Control

On DNA isolated from the 8 mouse tumors, Southern blot analysis was performed as described previously. Probes used for Southern blot were the following:

Trp53 Southern Probes
11F CTACCTGAAGACCAAGAAGG
11R TGGAGGATATGGACCCTATG
