# Supplementary material for: Somatic structural rearrangements in genetically engineered mouse mammary tumors
Source: Genome Biol. 2010 Oct 13;11(10):R100. doi: 10.1186/gb-2010-11-10-r100 (PMC3218656; doi:10.1186/gb-2010-11-10-r100)
Supplement: Additional file 4 — Copy number analysis of the LRP1b locus in 102 sporadic human breast cancers. [file gb-2010-11-10-r100-S4.PDF]

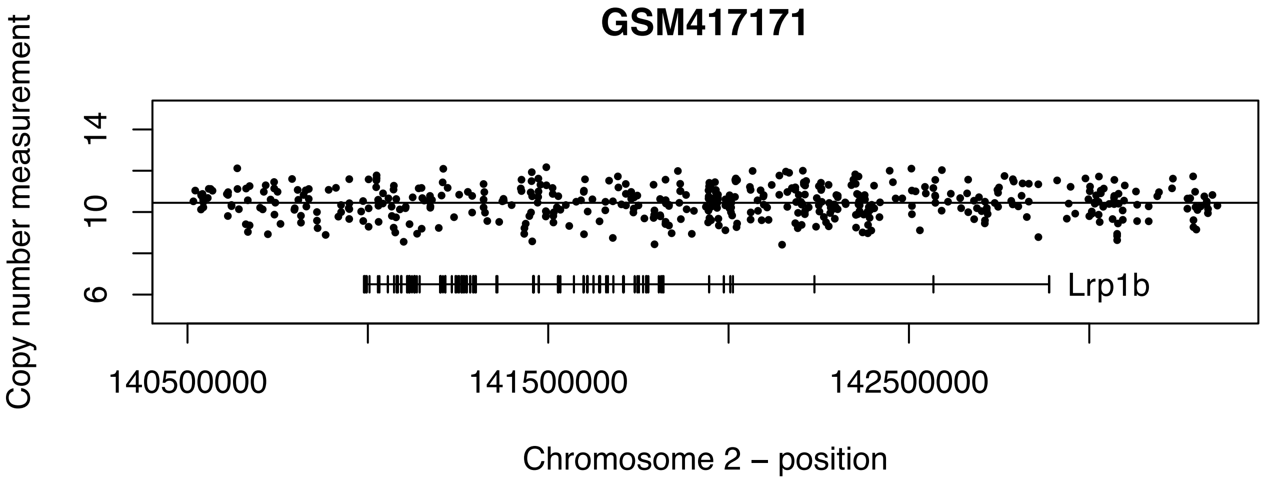
GSM417171
Copy number measurement
6
10
14
140500000
141500000
142500000
Chromosome 2 – position
Lrp1b

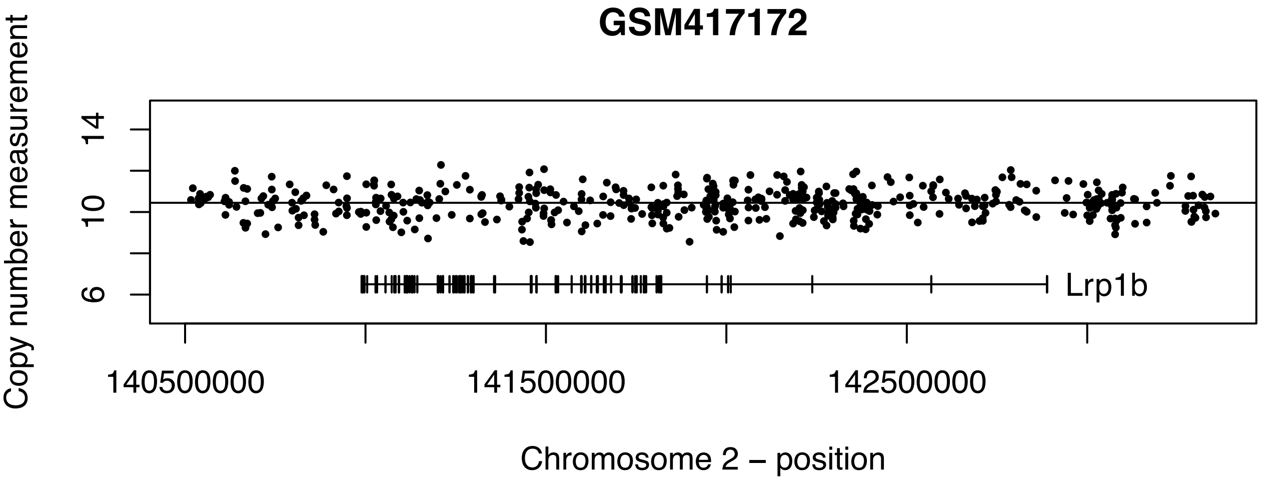
GSM417172
Copy number measurement
14
10
6
Lrp1b
140500000
141500000
142500000
Chromosome 2 – position

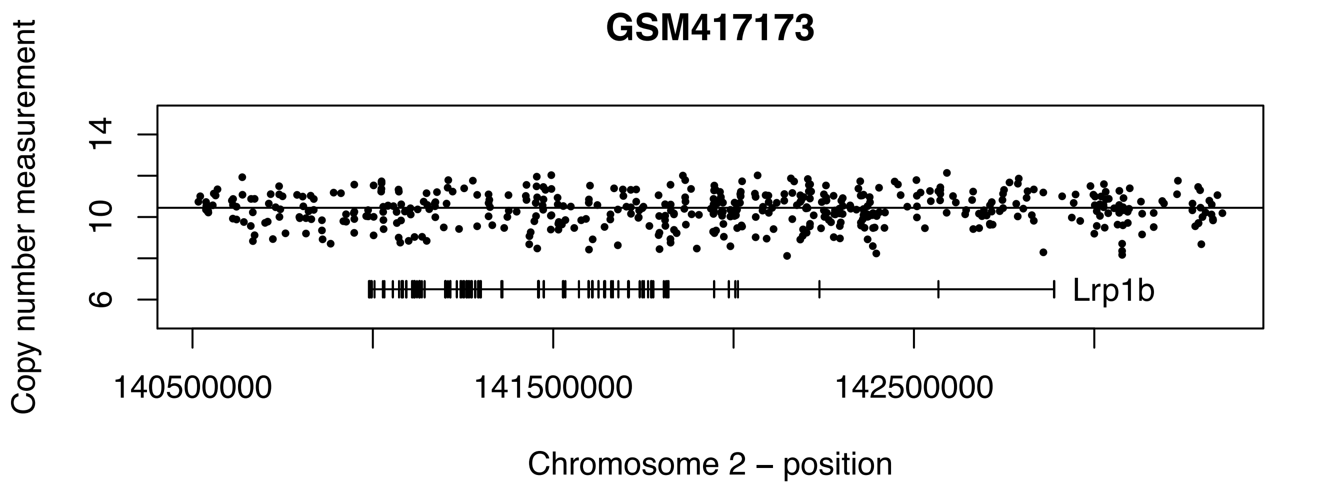

GSM417173
Copy number measurement
14
10
6
Lrp1b
140500000
141500000
142500000
Chromosome 2 – position

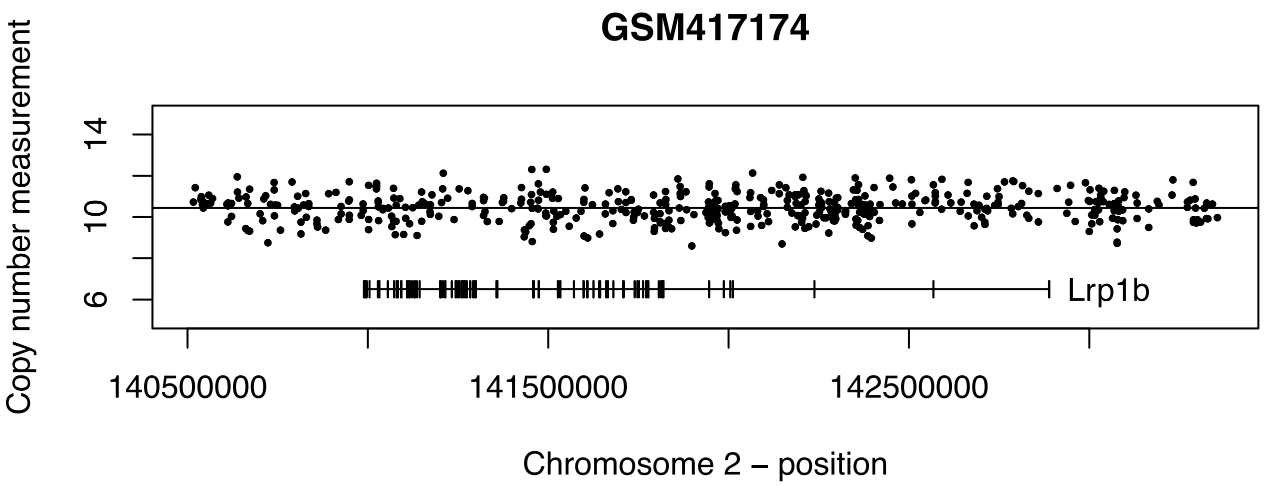
GSM417174
Copy number measurement
14
10
6
Lrp1b
140500000
141500000
142500000
Chromosome 2 – position

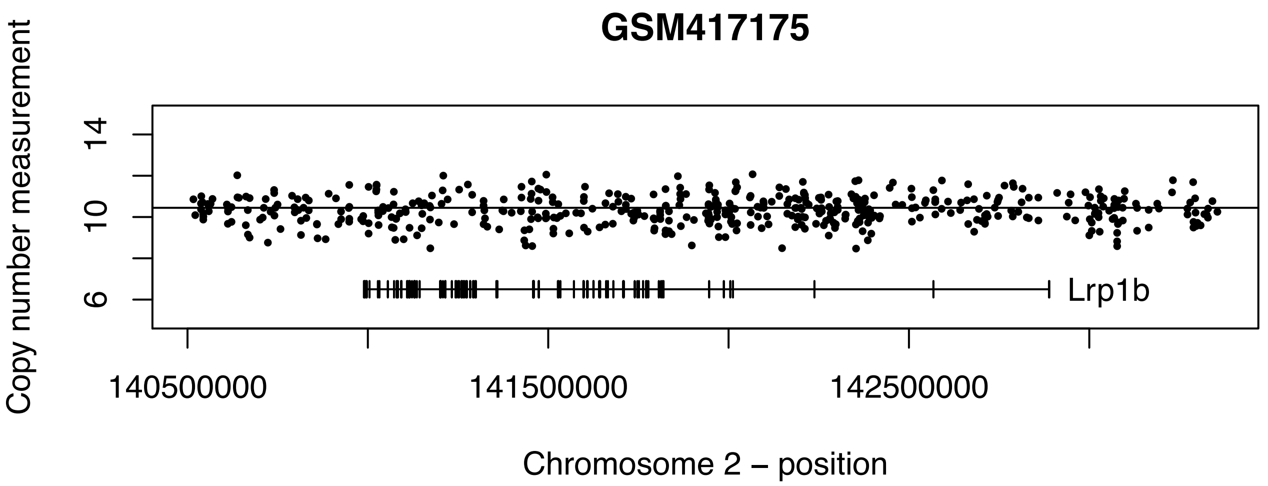
GSM417175
Copy number measurement
14
10
6
Lrp1b
140500000
141500000
142500000
Chromosome 2 – position

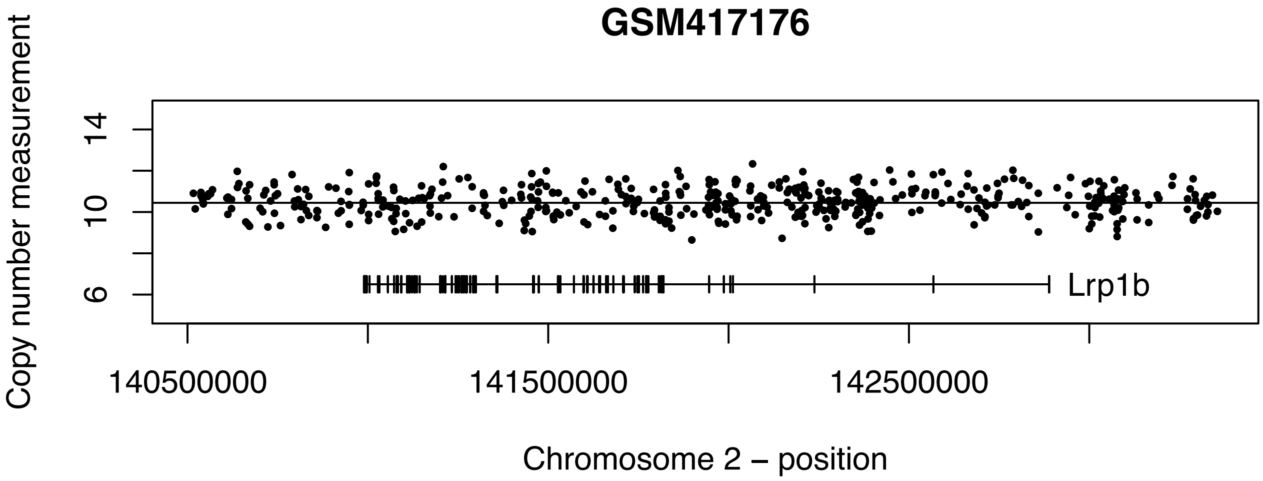
GSM417176
Copy number measurement
14
10
6
Lrp1b
140500000
141500000
142500000
Chromosome 2 – position

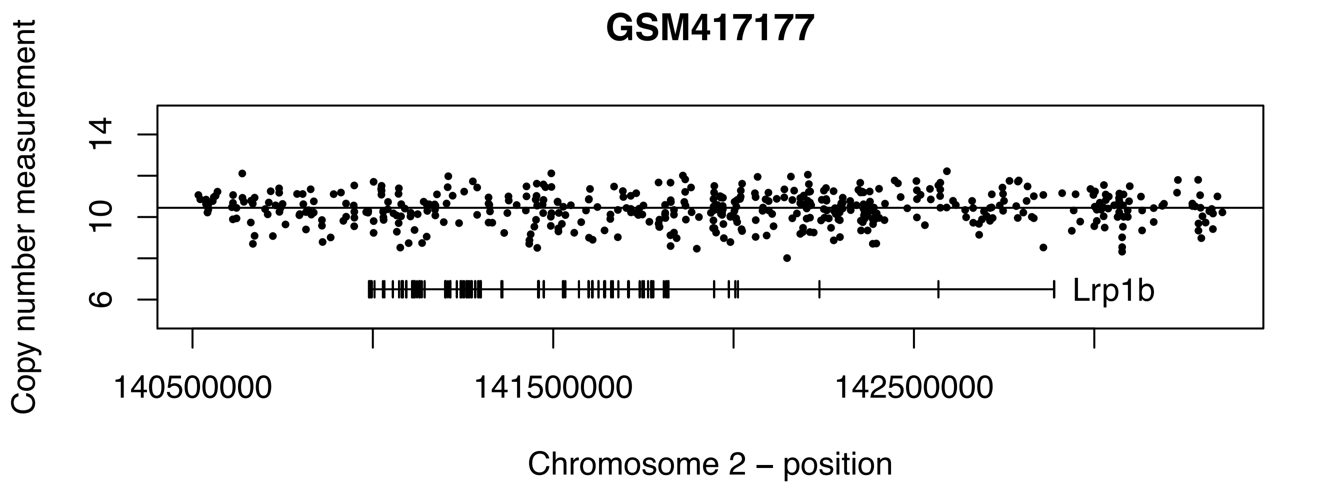
GSM417177
Copy number measurement
14
10
6
Lrp1b
140500000
141500000
142500000
Chromosome 2 – position

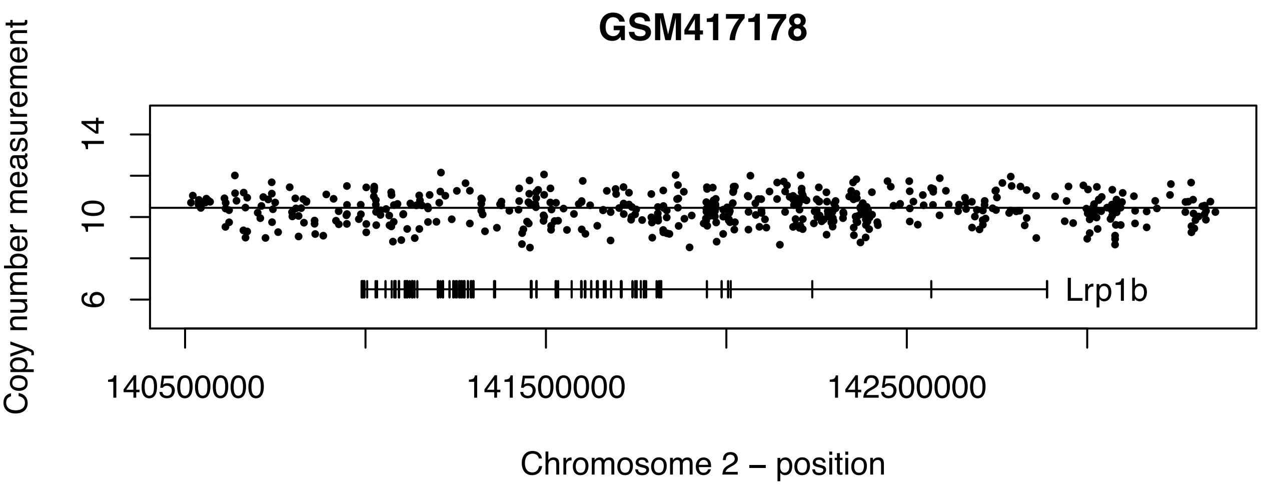
GSM417178
Copy number measurement
14
10
6
Lrp1b
140500000
141500000
142500000
Chromosome 2 – position

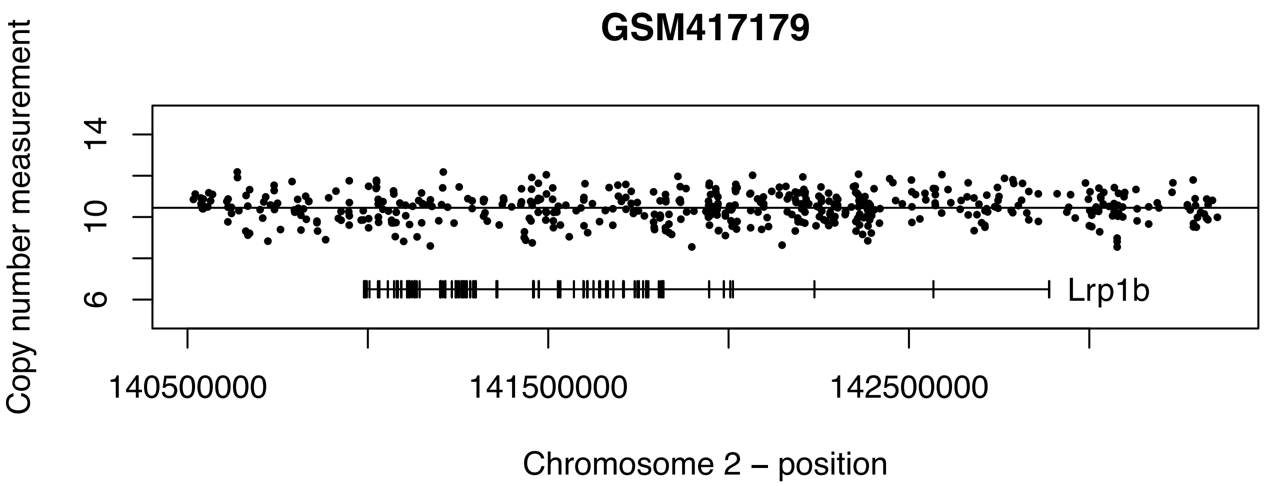
GSM417179
Copy number measurement
14
10
6
Lrp1b
140500000
141500000
142500000
Chromosome 2 – position

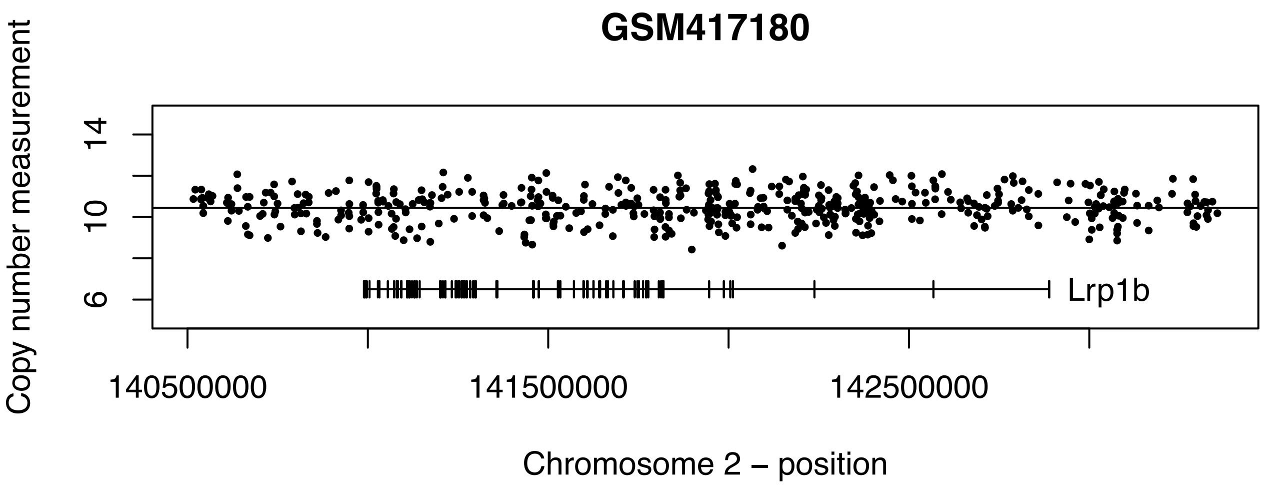
GSM417180
Copy number measurement
14
10
6
Lrp1b
140500000
141500000
142500000
Chromosome 2 – position

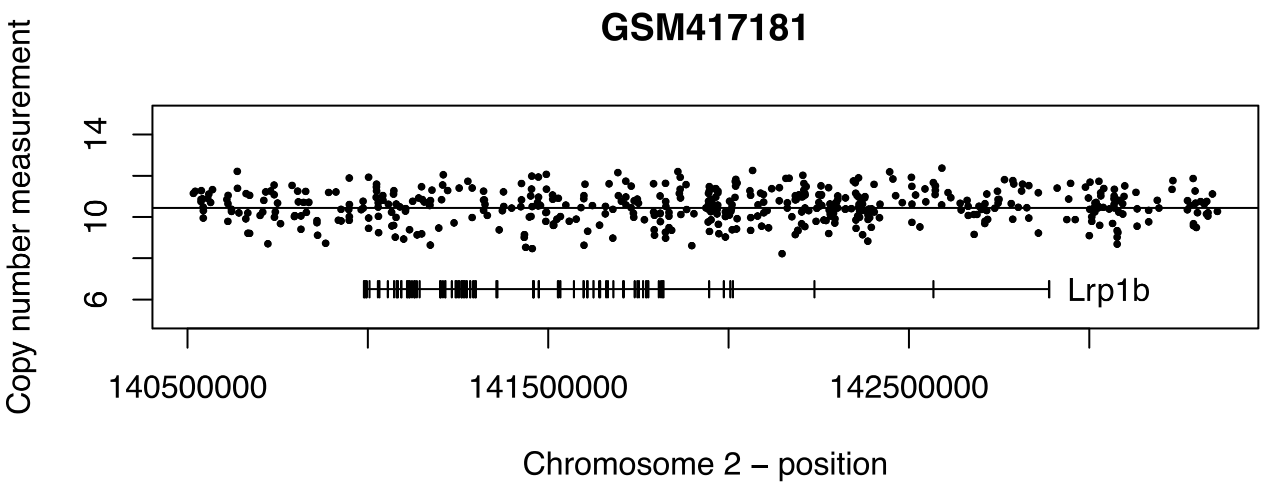
GSM417181
Copy number measurement
14
10
6
Lrp1b
140500000
141500000
142500000
Chromosome 2 – position

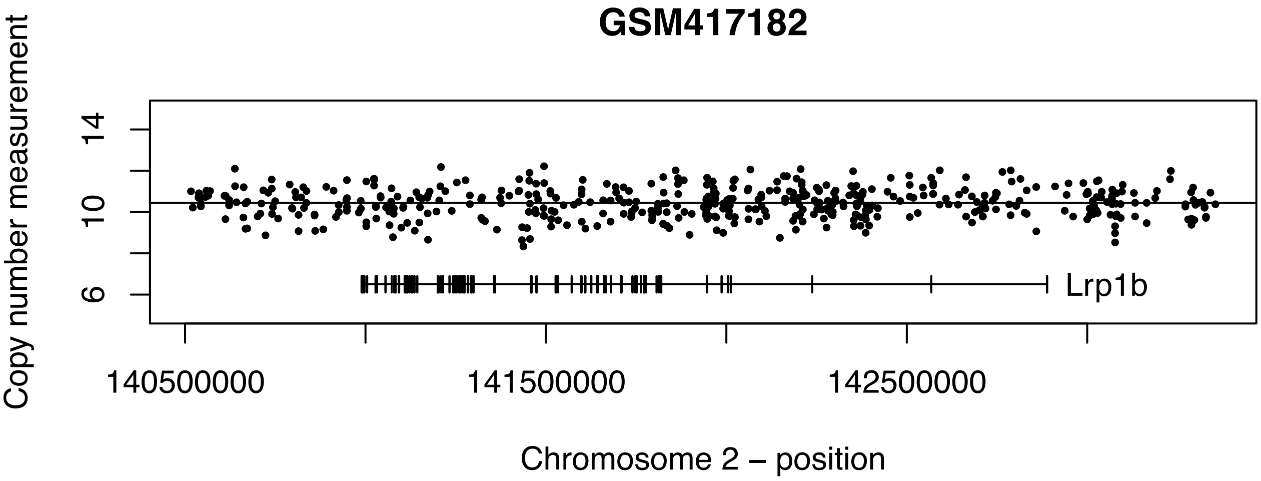
GSM417182
Copy number measurement
14
10
6
Lrp1b
140500000
141500000
142500000
Chromosome 2 – position

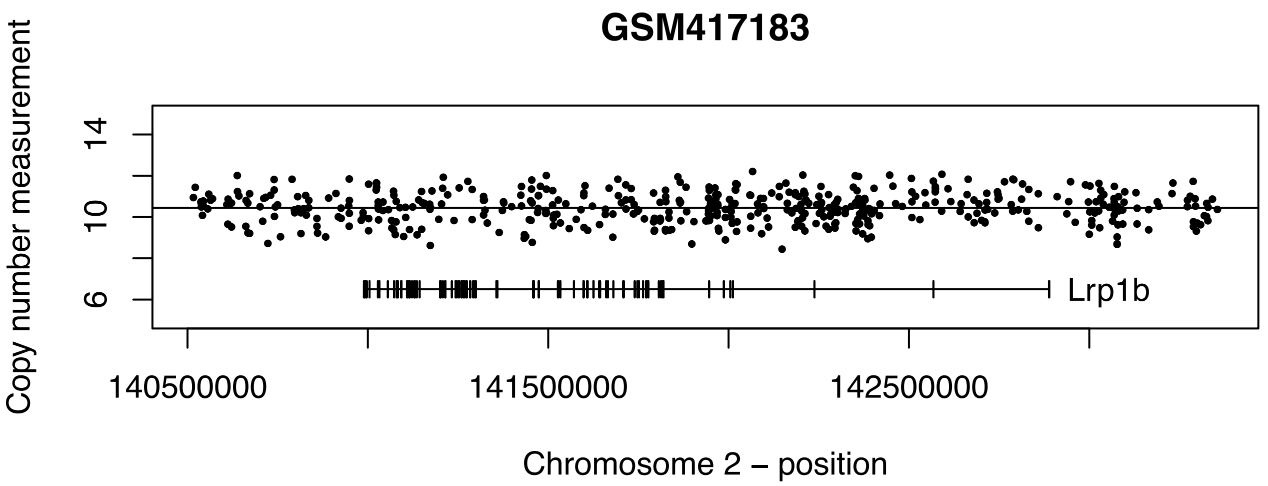
GSM417183
Copy number measurement
14
10
6
Lrp1b
140500000
141500000
142500000
Chromosome 2 – position

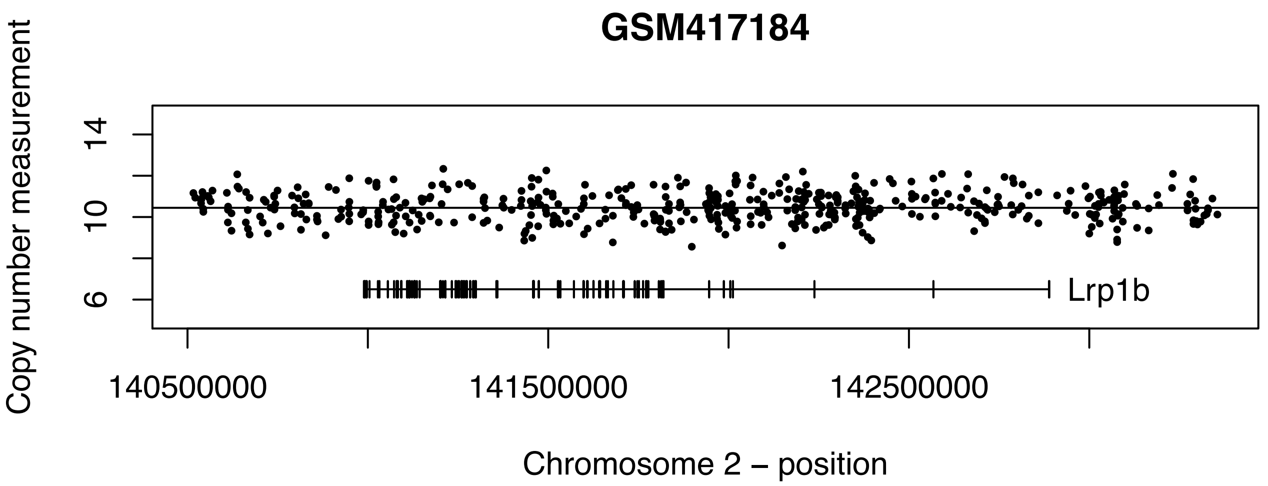

GSM417184
Copy number measurement
14
10
6
Lrp1b
140500000
141500000
142500000
Chromosome 2 – position

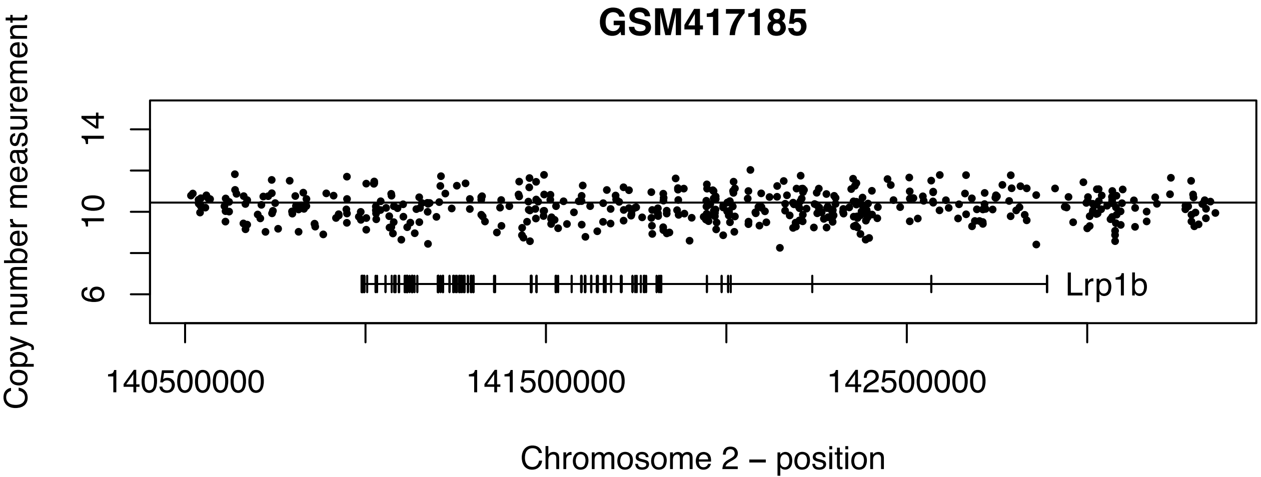

GSM417185
Copy number measurement
6
10
14
Lrp1b
140500000
141500000
142500000
Chromosome 2 – position

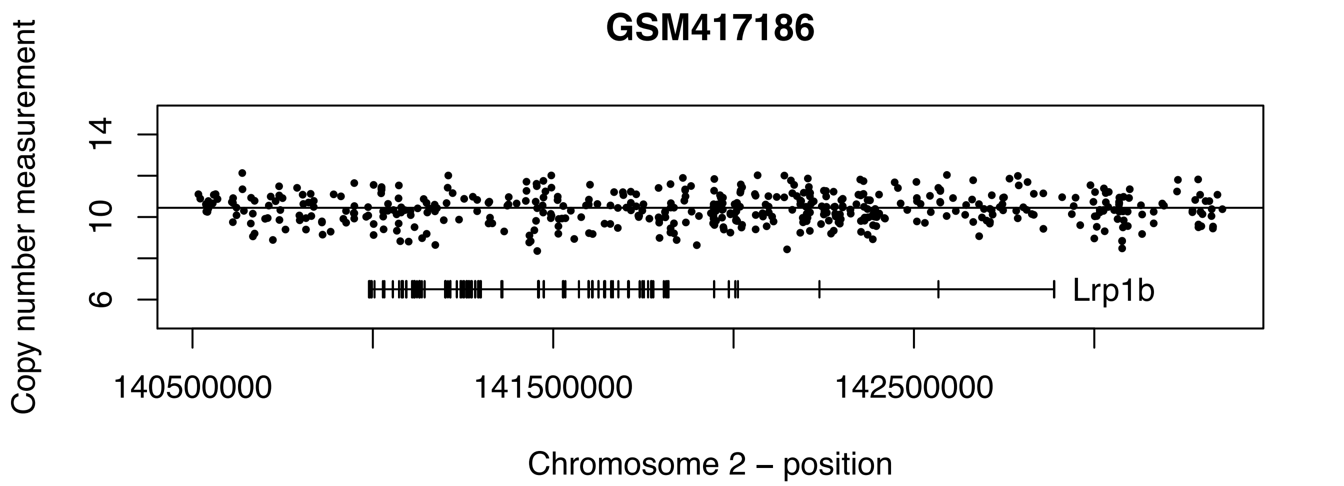

GSM417186
Copy number measurement
14
10
6
Lrp1b
140500000
141500000
142500000
Chromosome 2 – position

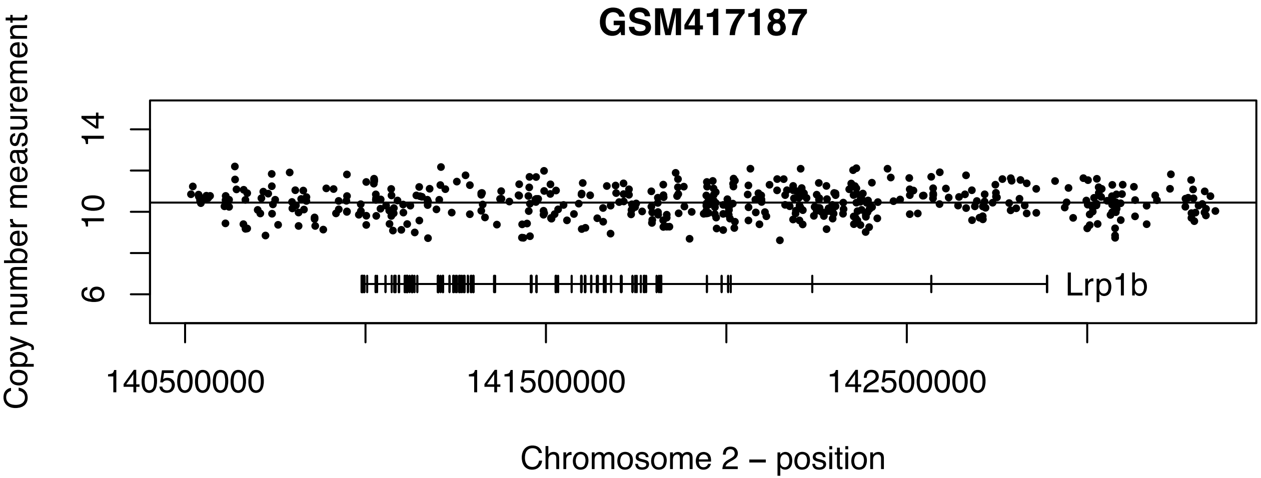
GSM417187
Copy number measurement
14
10
6
Lrp1b
140500000
141500000
142500000
Chromosome 2 – position

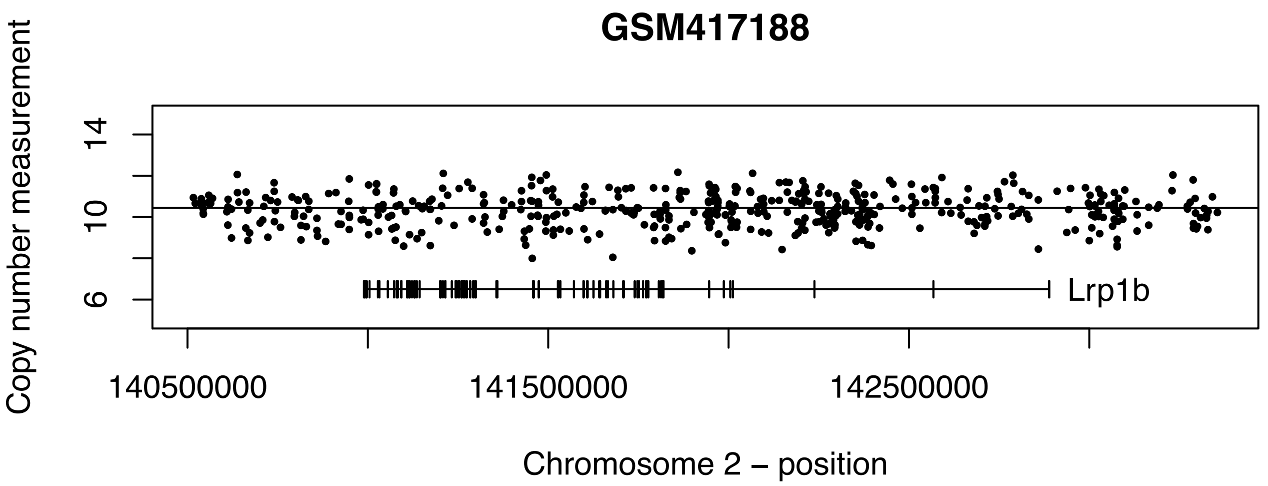
GSM417188
Copy number measurement
14
10
6
Lrp1b
140500000
141500000
142500000
Chromosome 2 – position

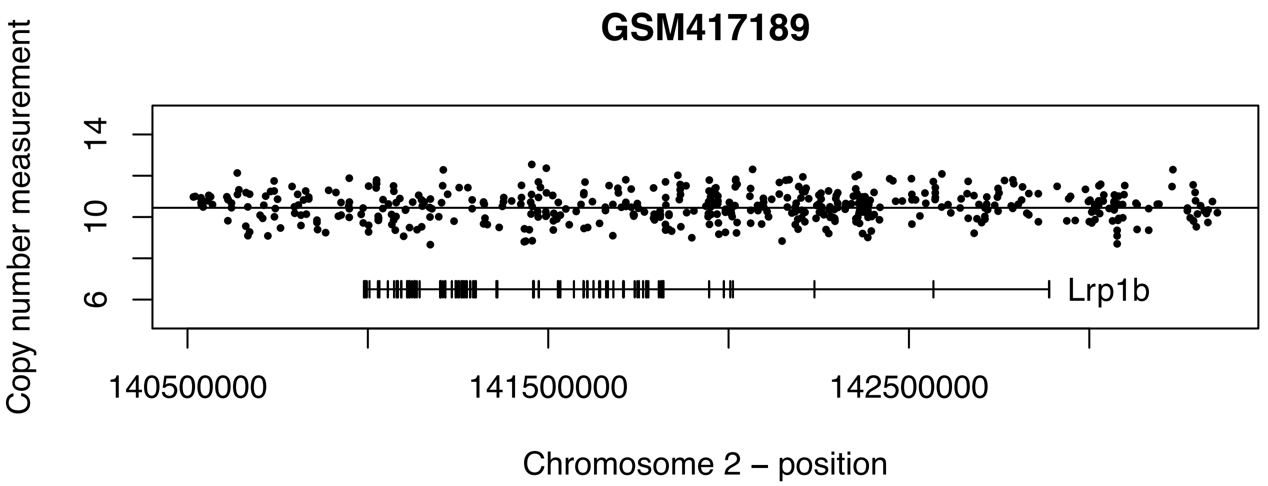
GSM417189
Copy number measurement
14
10
6
Lrp1b
140500000
141500000
142500000
Chromosome 2 – position

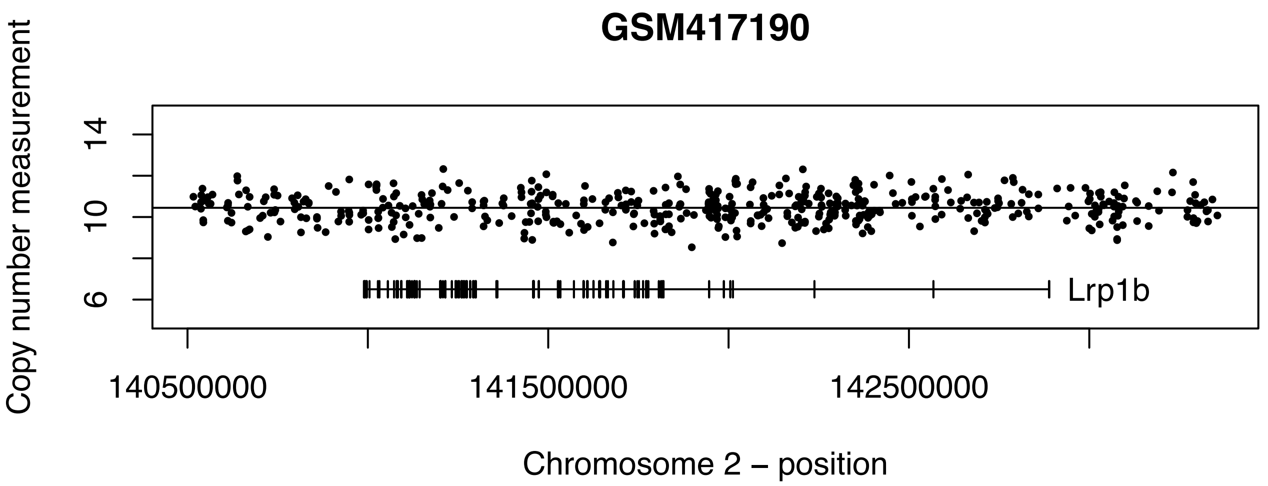
GSM417190
Copy number measurement
14
10
6
Lrp1b
140500000
141500000
142500000
Chromosome 2 – position

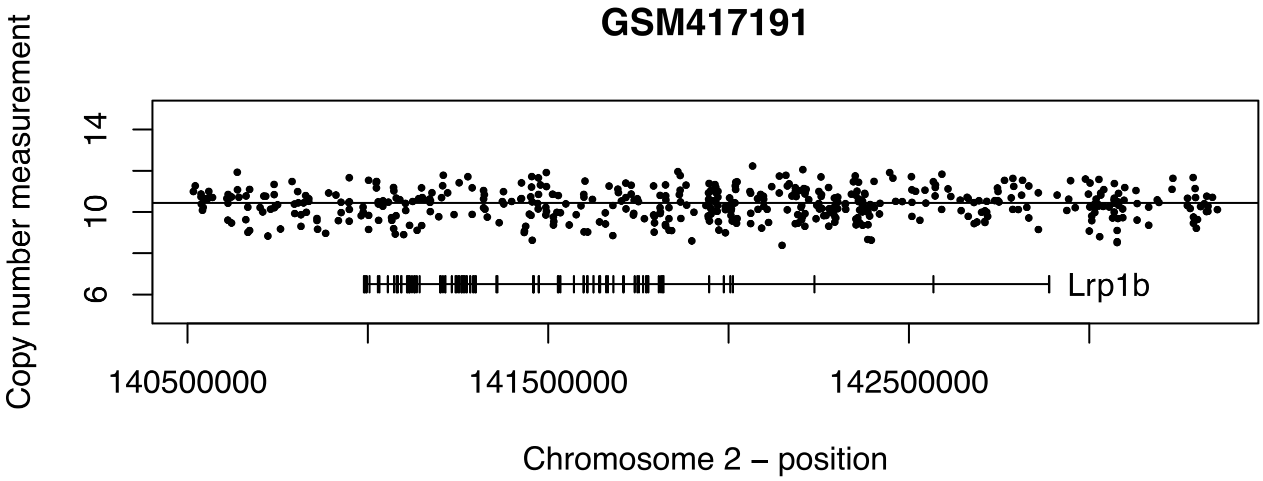
GSM417191
Copy number measurement
14
10
6
Lrp1b
140500000
141500000
142500000
Chromosome 2 – position

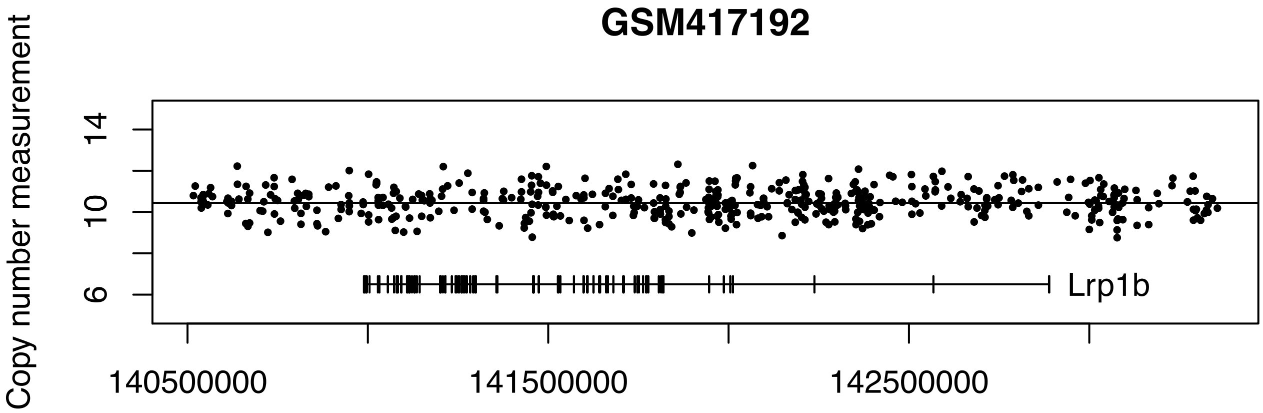

GSM417192
Copy number measurement
6
10
14
Lrp1b
140500000
141500000
142500000
Chromosome 2 – position

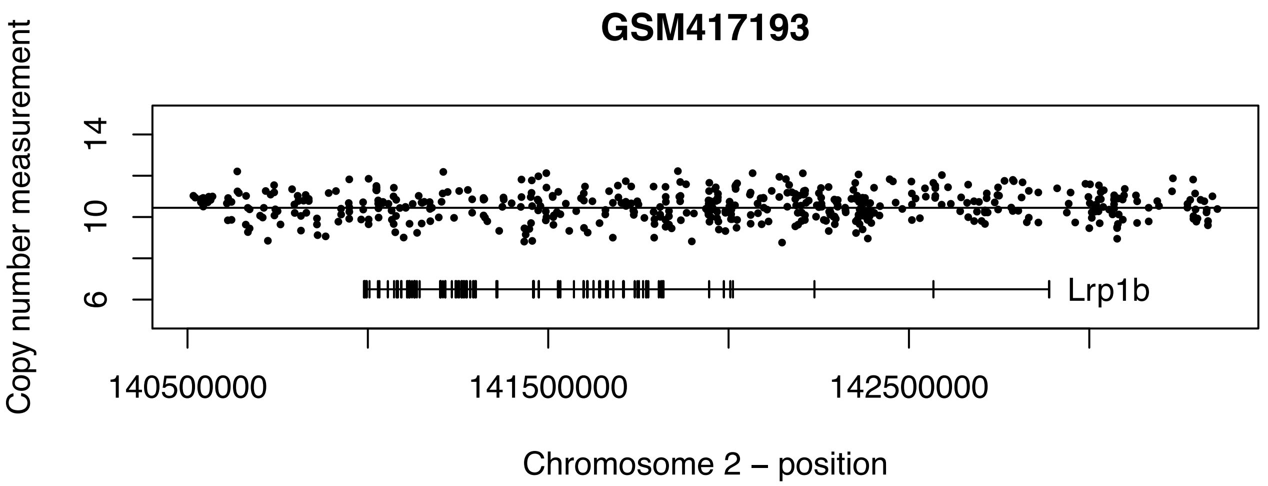
GSM417193
Copy number measurement
14
10
6
Lrp1b
140500000
141500000
142500000
Chromosome 2 – position

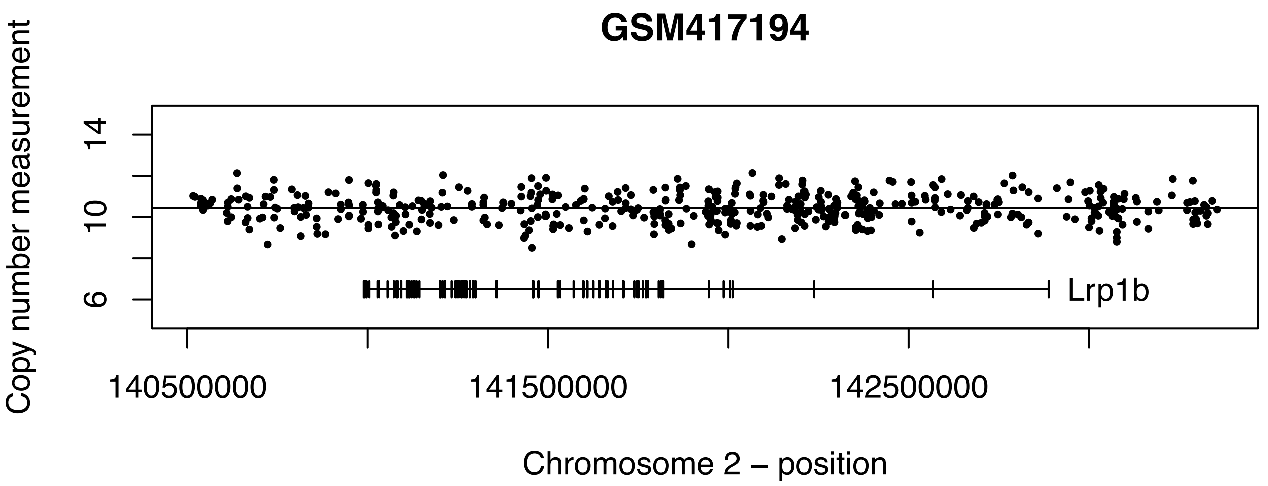

GSM417194
Copy number measurement
14
10
6
Lrp1b
140500000
141500000
142500000
Chromosome 2 – position

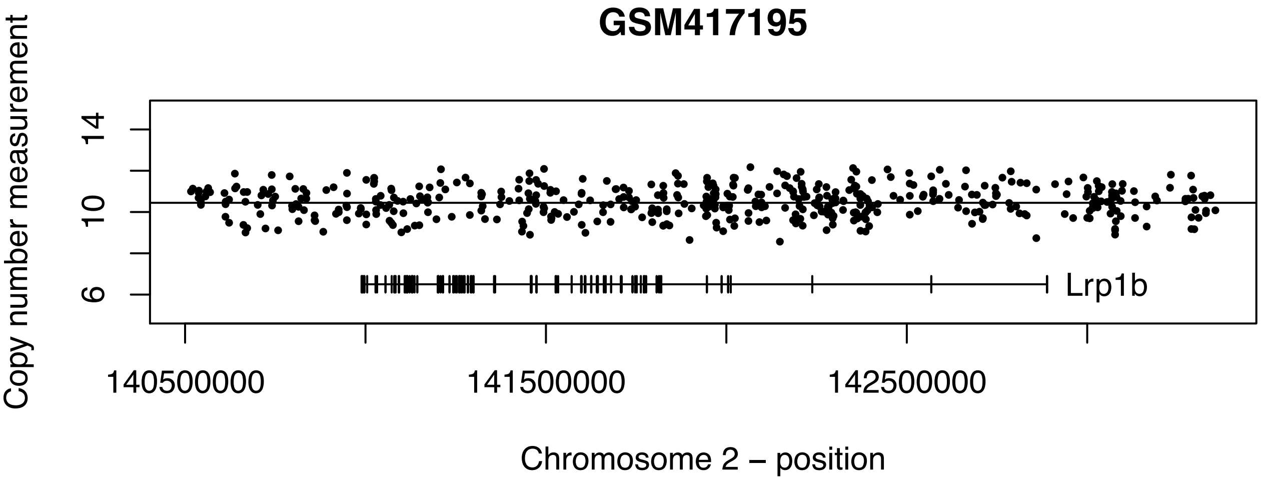

GSM417195
Copy number measurement
14
10
6
Lrp1b
140500000
141500000
142500000
Chromosome 2 – position

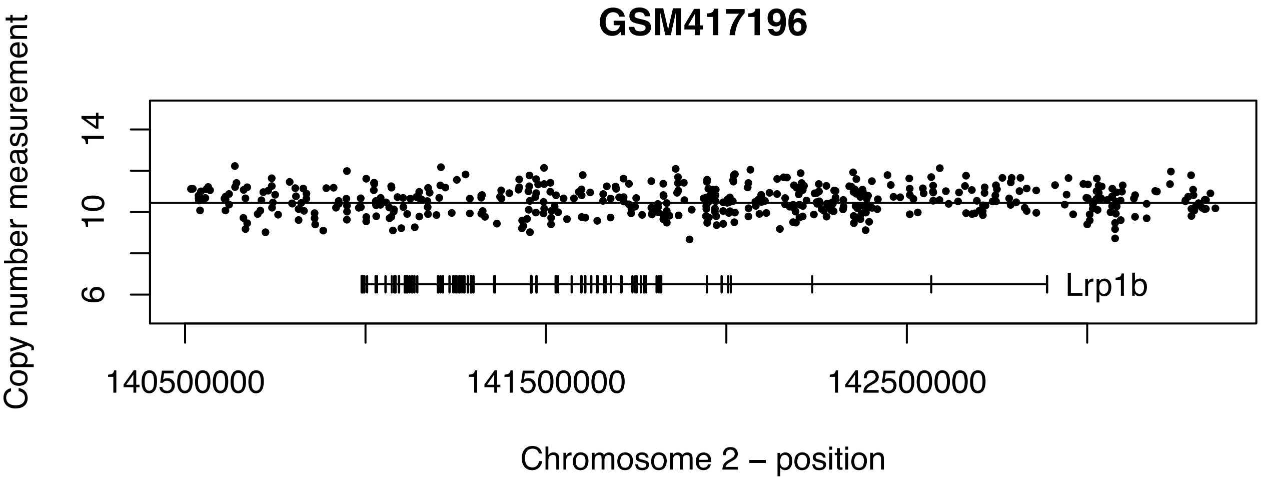

GSM417196
Copy number measurement
14
10
6
Lrp1b
140500000
141500000
142500000
Chromosome 2 – position

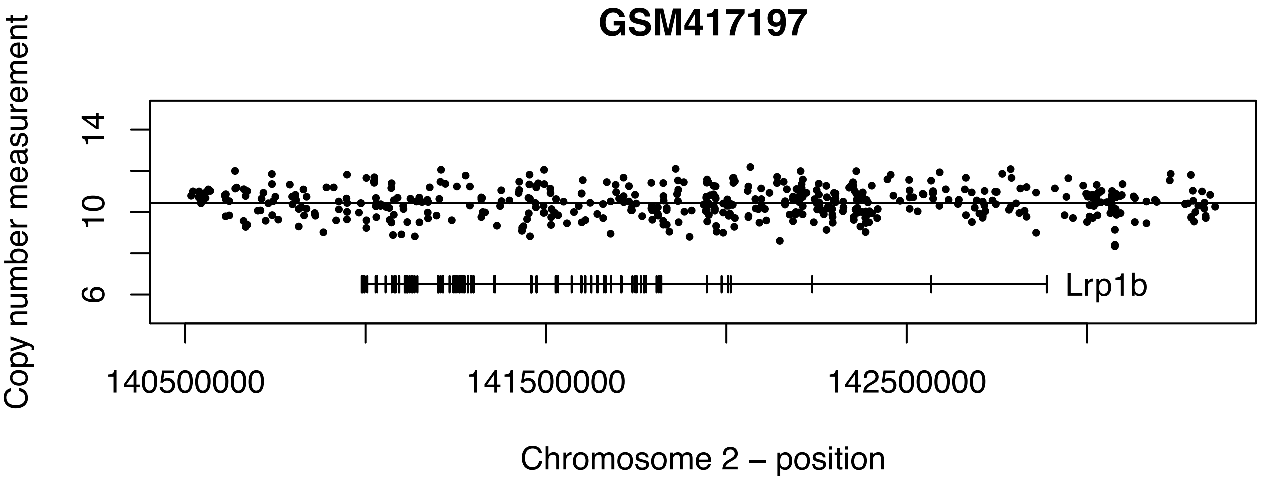

GSM417197
Copy number measurement
14
10
6
Lrp1b
140500000
141500000
142500000
Chromosome 2 – position

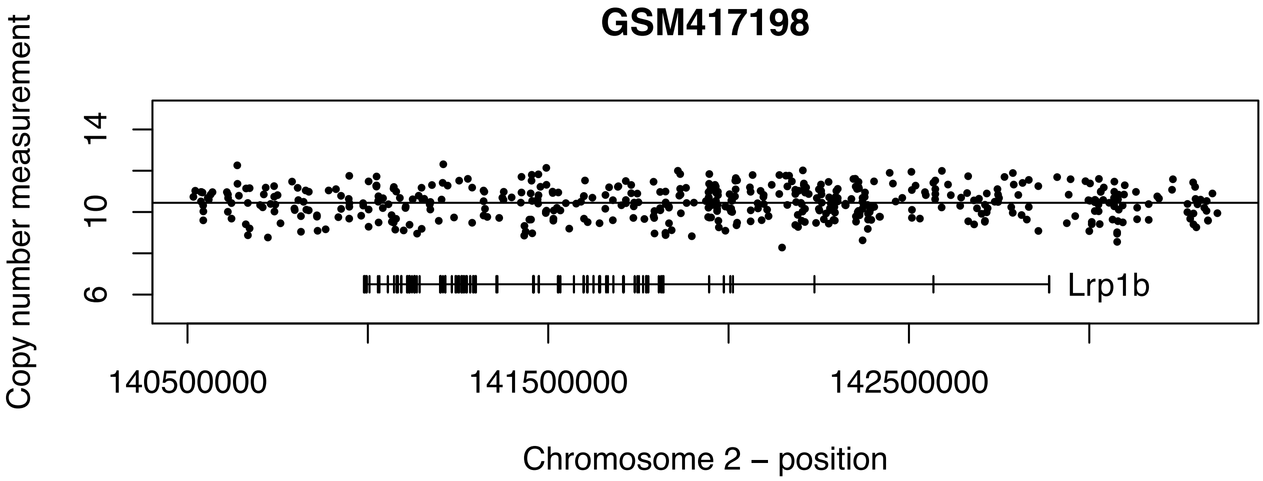

GSM417198
Copy number measurement
14
10
6
Lrp1b
140500000
141500000
142500000
Chromosome 2 – position

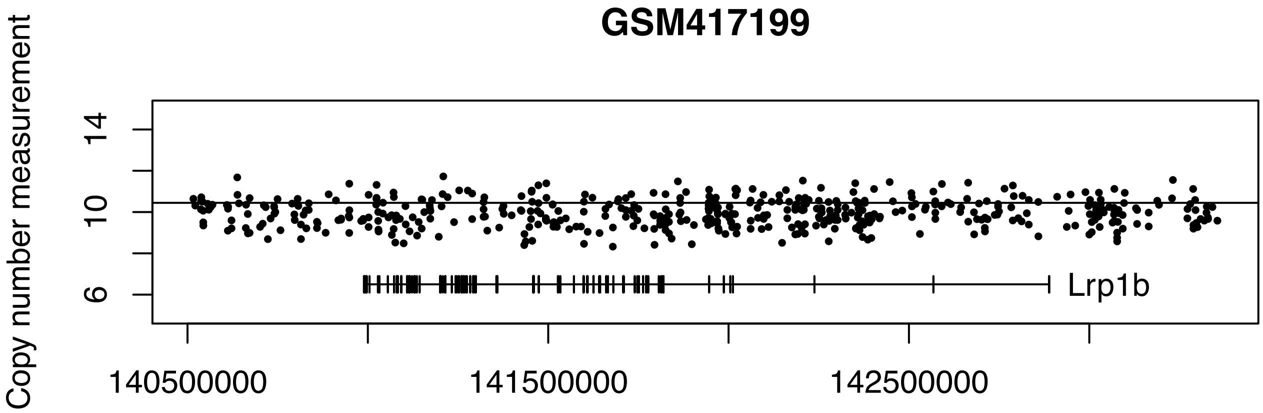
GSM417199
Copy number measurement
14
10
6
Lrp1b
140500000
141500000
142500000
Chromosome 2 – position

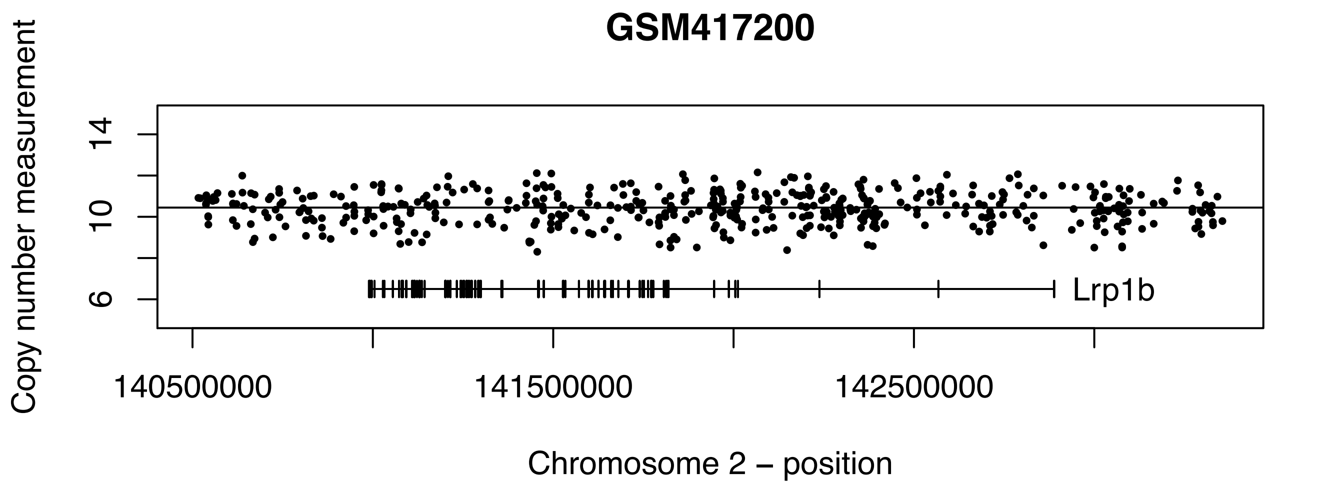
GSM417200
Copy number measurement
14
10
6
Lrp1b
140500000
141500000
142500000
Chromosome 2 – position

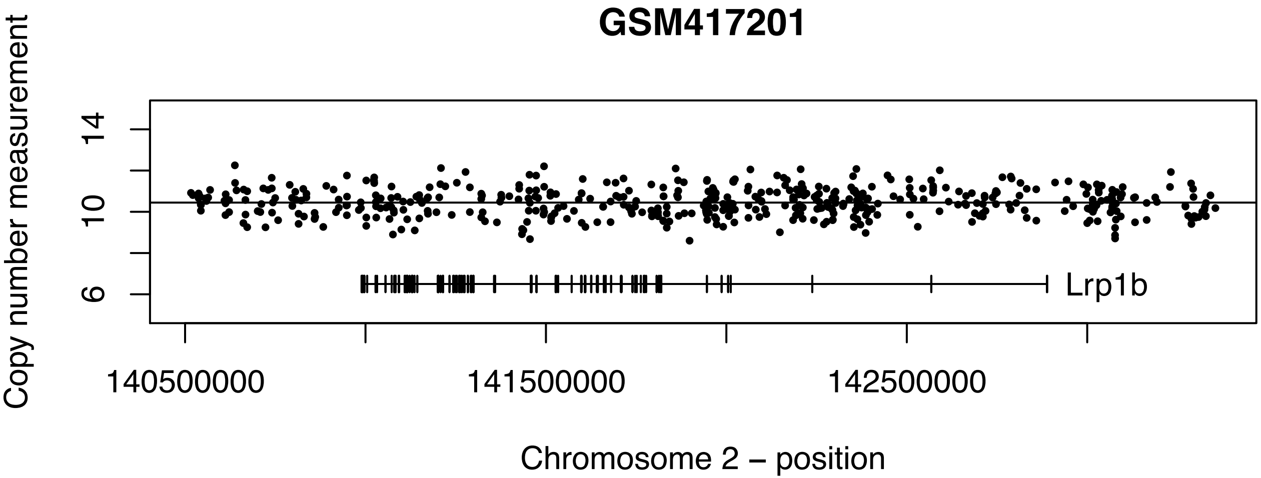

GSM417201
Copy number measurement
14
10
6
Lrp1b
140500000
141500000
142500000
Chromosome 2 – position

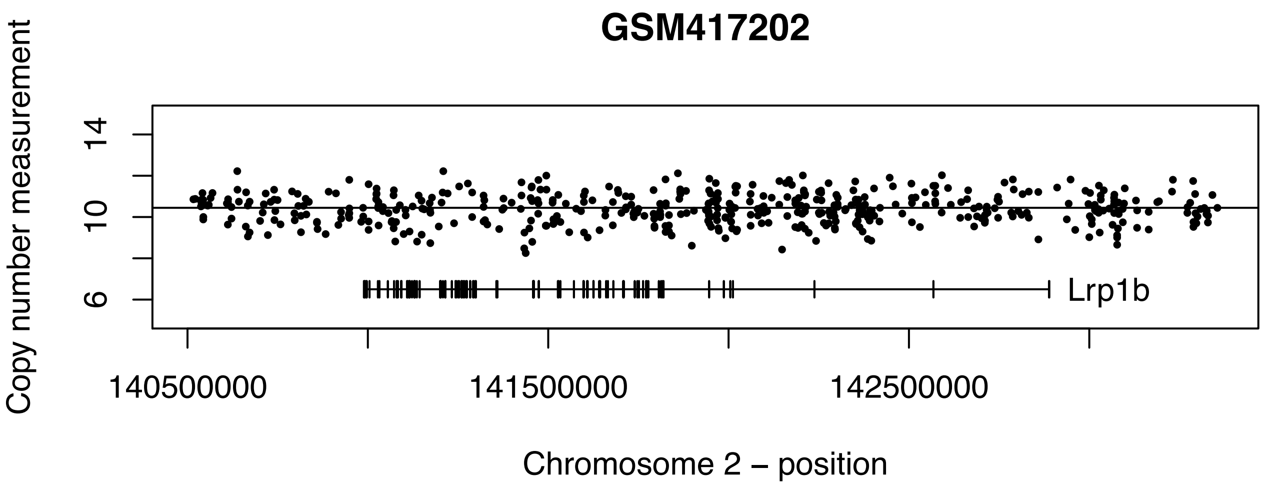
GSM417202
Copy number measurement
14
10
6
Lrp1b
140500000
141500000
142500000
Chromosome 2 – position

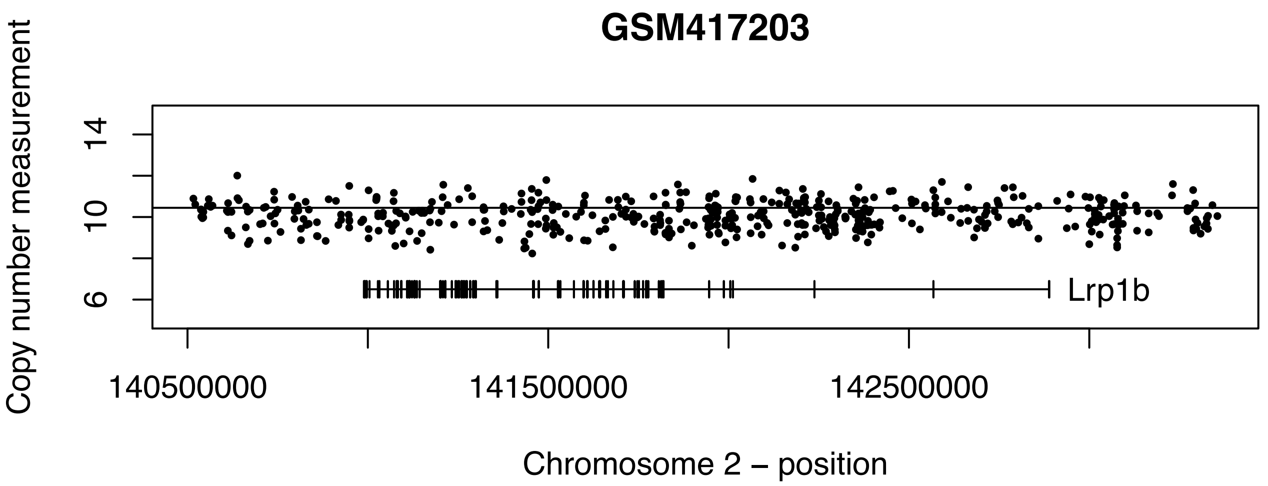
GSM417203
Copy number measurement
14
10
6
Lrp1b
140500000
141500000
142500000
Chromosome 2 – position

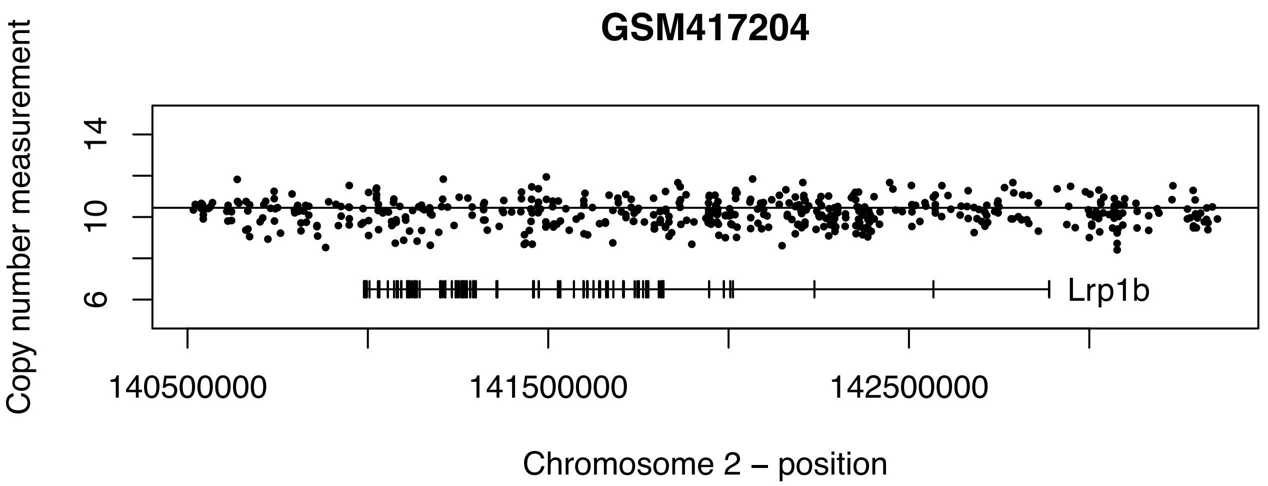
GSM417204
Copy number measurement
6
10
14
Lrp1b
140500000
141500000
142500000
Chromosome 2 – position

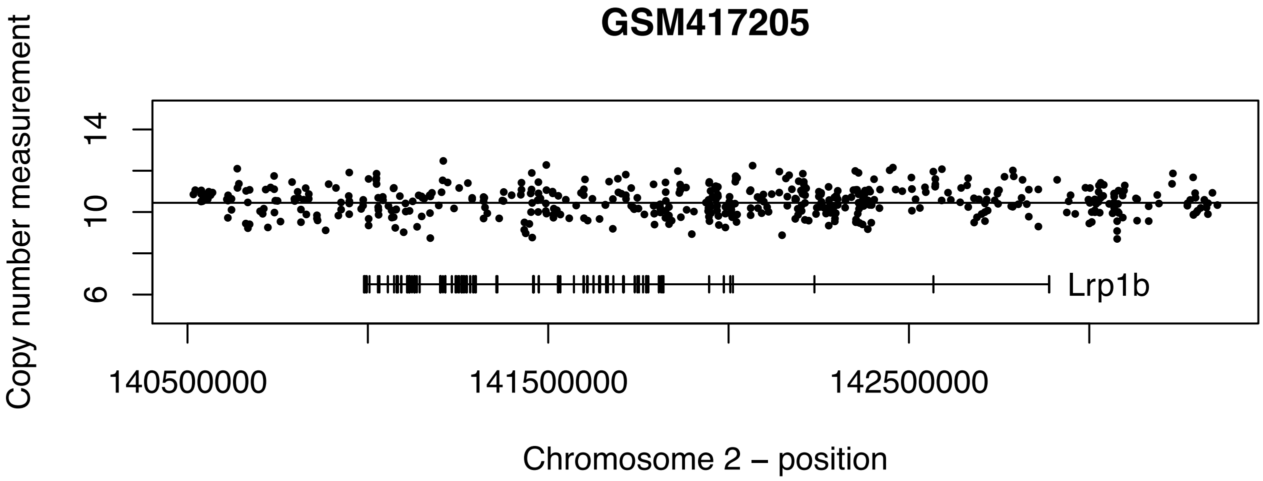
GSM417205
Copy number measurement
14
10
6
Lrp1b
140500000
141500000
142500000
Chromosome 2 – position

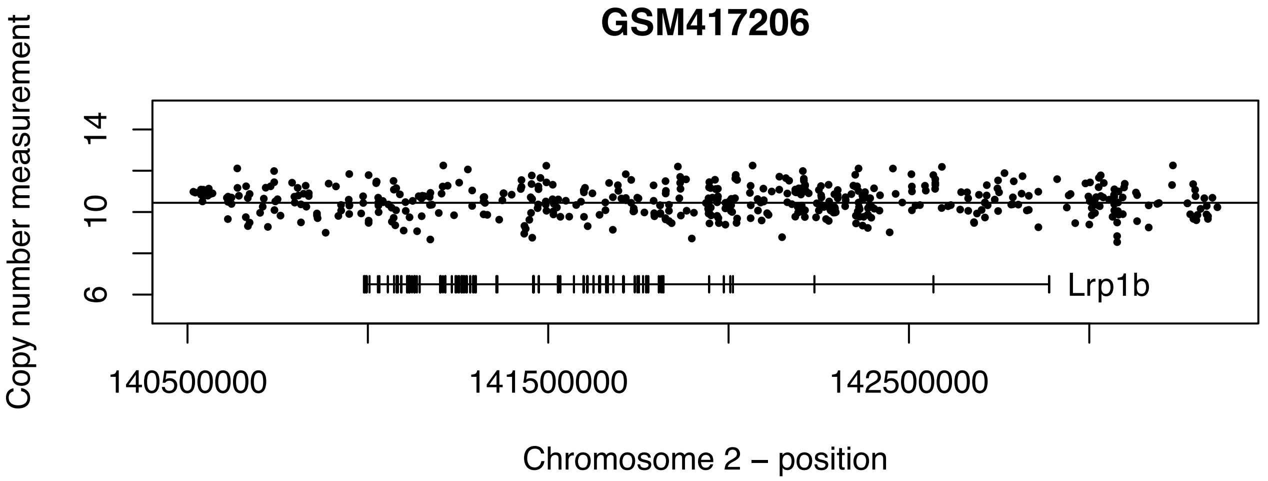
GSM417206
Copy number measurement
14
10
6
Lrp1b
140500000
141500000
142500000
Chromosome 2 – position

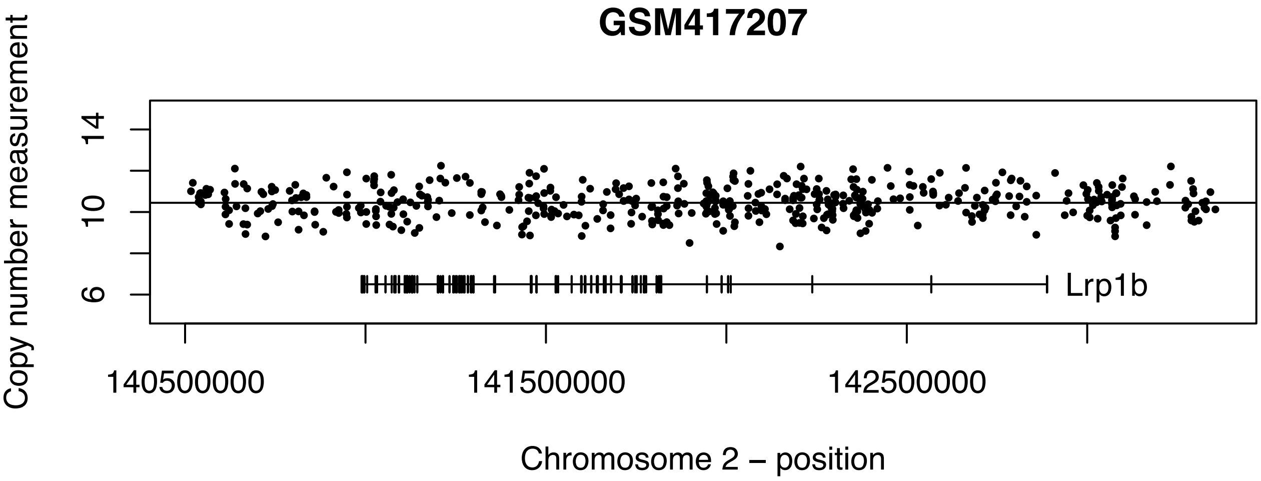
GSM417207
Copy number measurement
14
10
6
Lrp1b
140500000
141500000
142500000
Chromosome 2 – position

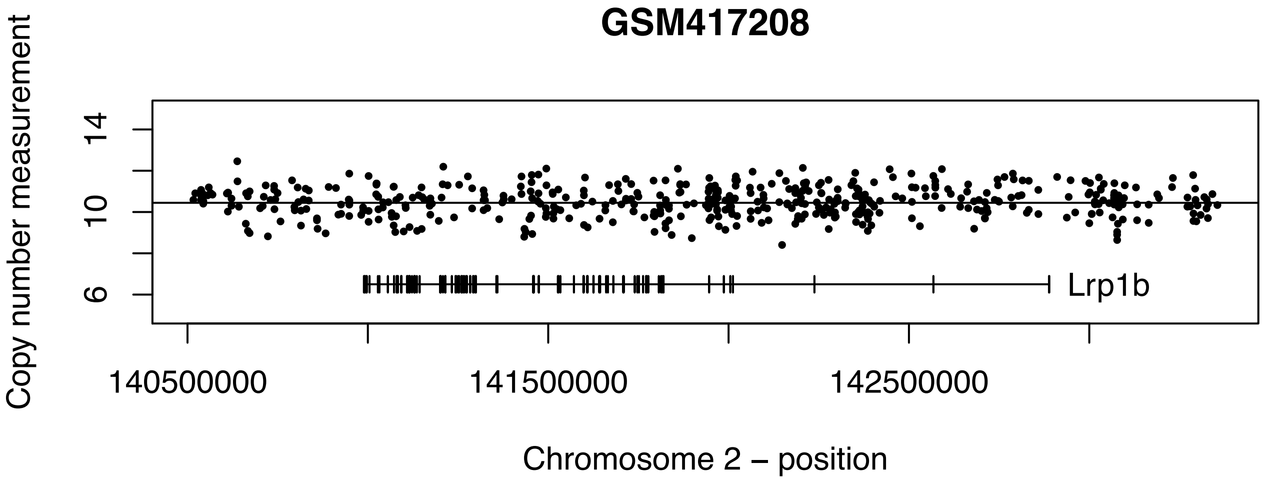
GSM417208
Copy number measurement
14
10
6
Lrp1b
140500000
141500000
142500000
Chromosome 2 – position

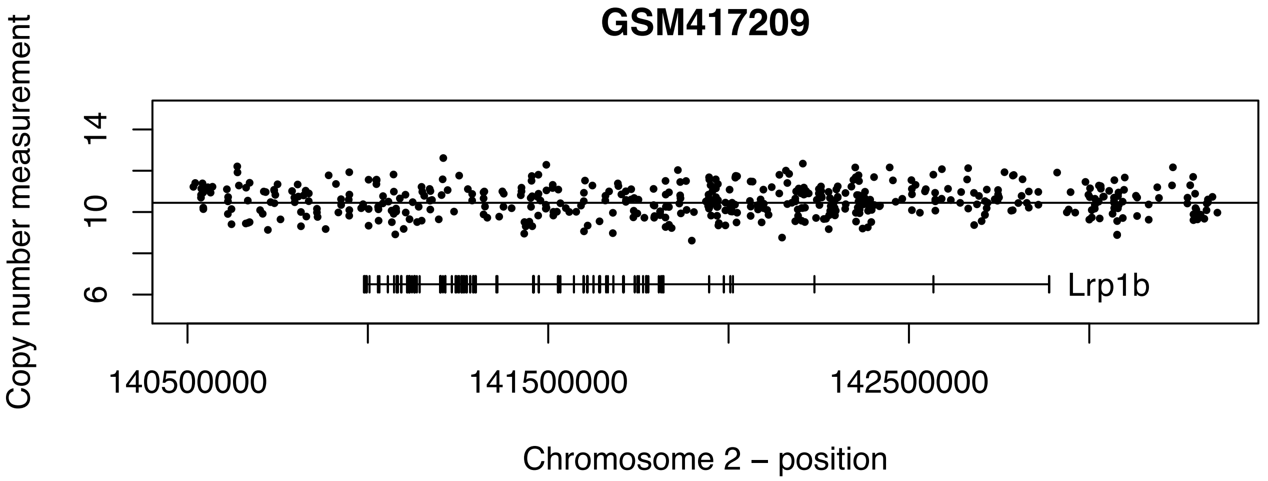
GSM417209
Copy number measurement
14
10
6
Lrp1b
140500000
141500000
142500000
Chromosome 2 – position

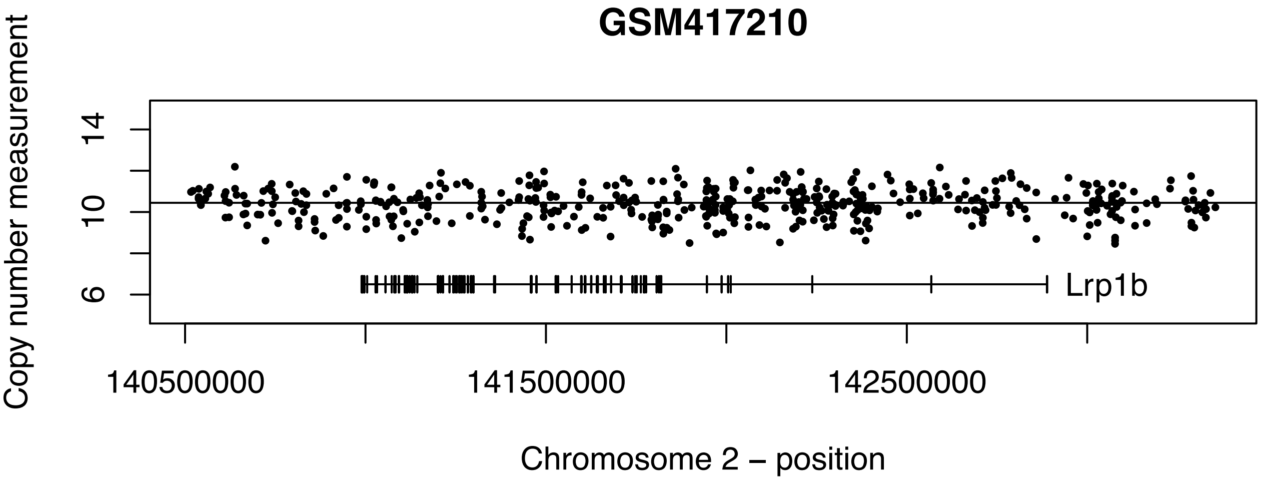

GSM417210
Copy number measurement
14
10
6
Lrp1b
140500000
141500000
142500000
Chromosome 2 – position

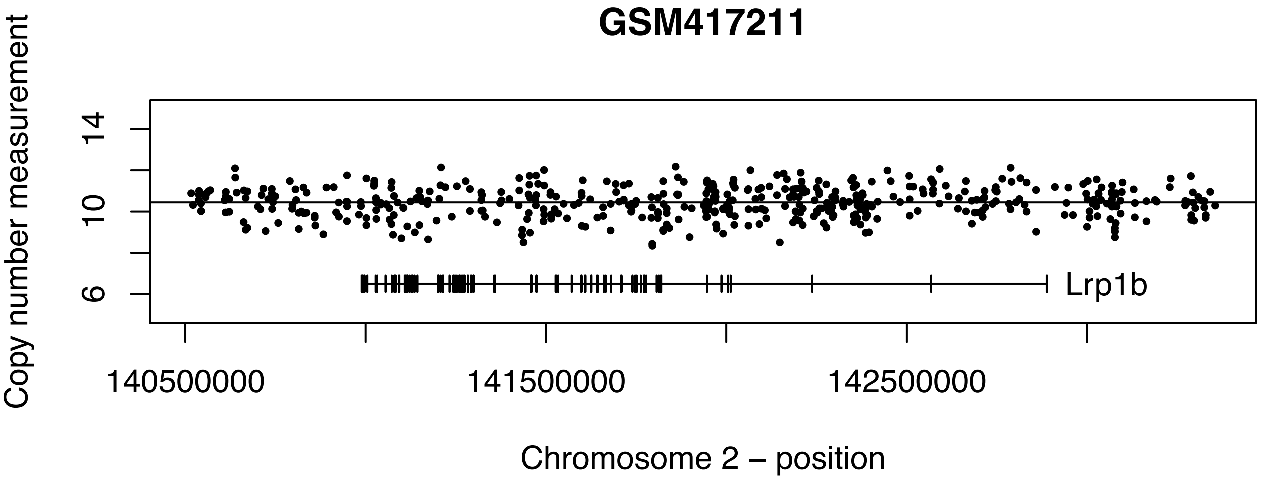
GSM417211
Copy number measurement
14
10
6
Lrp1b
140500000
141500000
142500000
Chromosome 2 – position

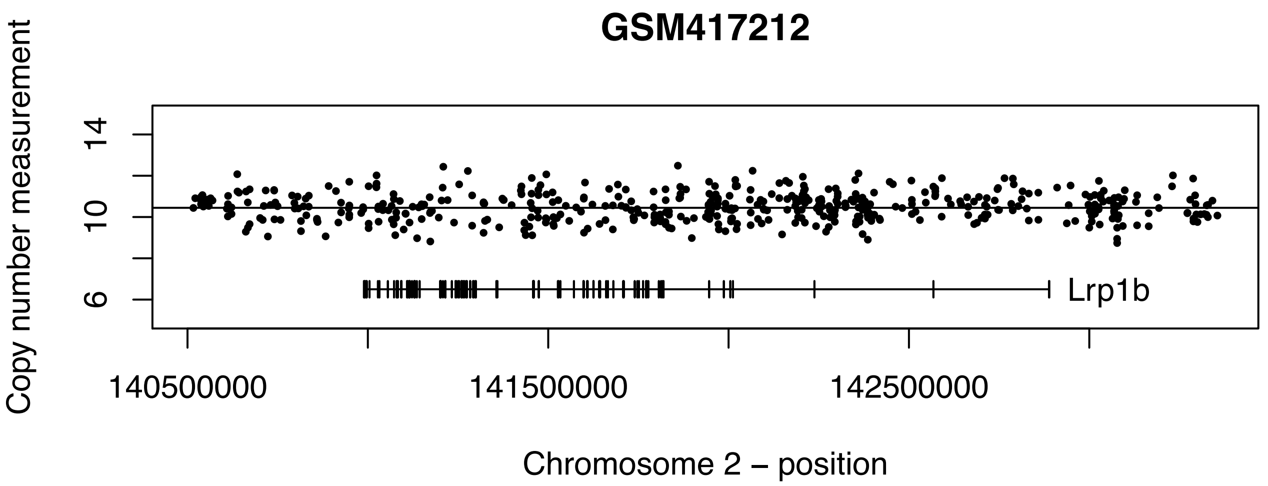
GSM417212
Copy number measurement
6
10
14
Lrp1b
140500000
141500000
142500000
Chromosome 2 – position

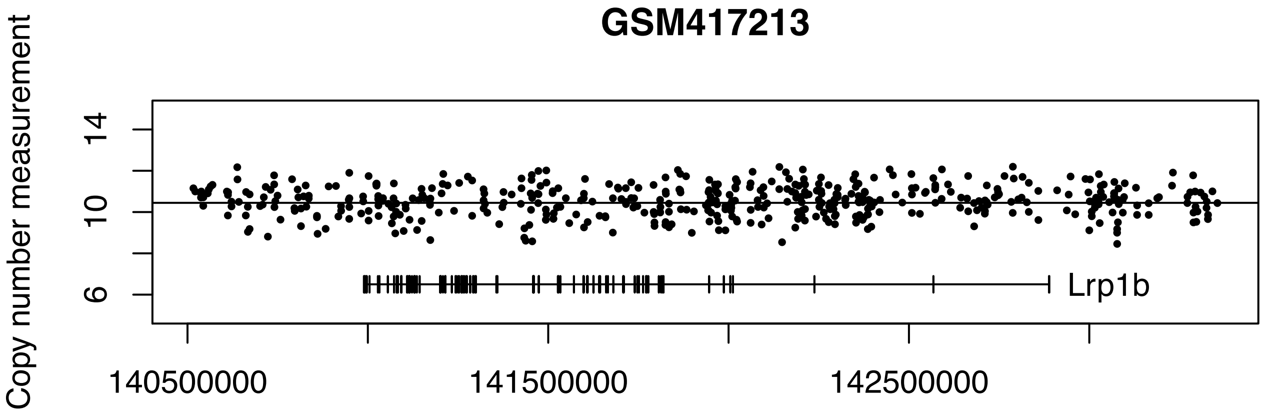
GSM417213
Copy number measurement
14
10
6
Lrp1b
140500000
141500000
142500000
Chromosome 2 – position

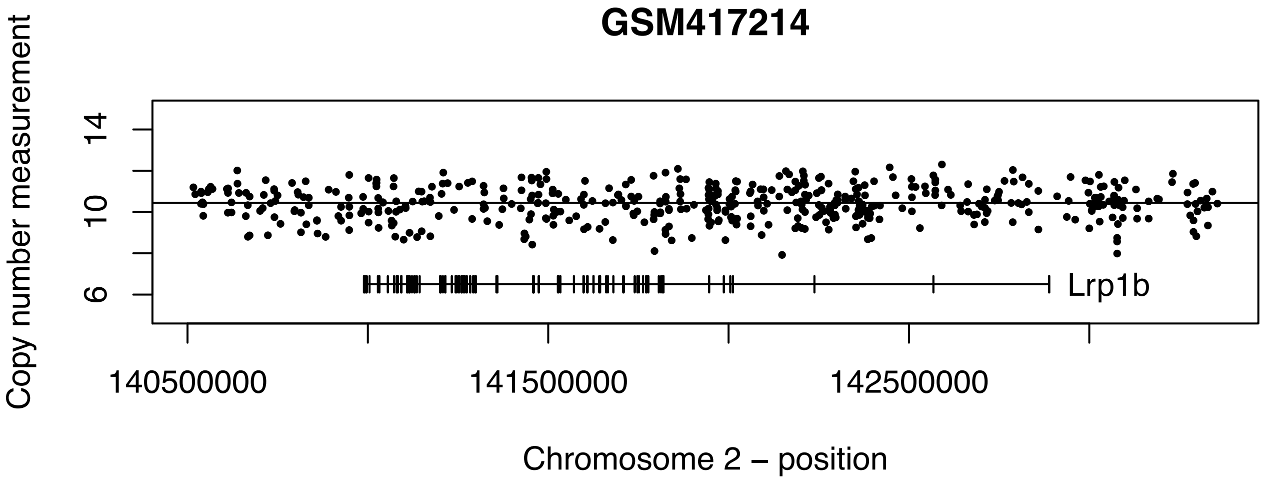
GSM417214
Copy number measurement
14
10
6
Lrp1b
140500000
141500000
142500000
Chromosome 2 – position

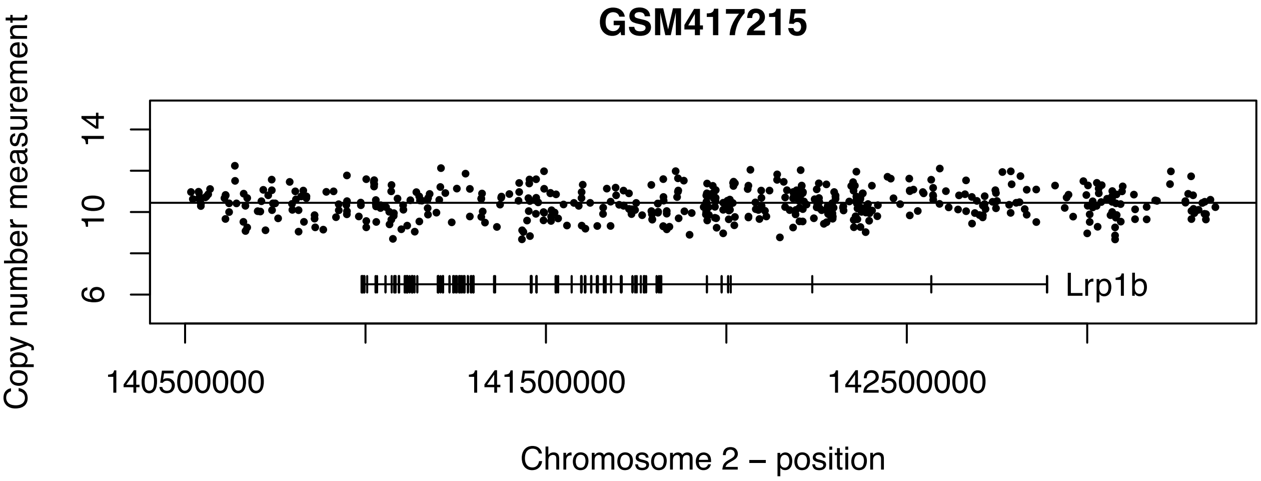

GSM417215
Copy number measurement
14
10
6
Lrp1b
140500000
141500000
142500000
Chromosome 2 – position

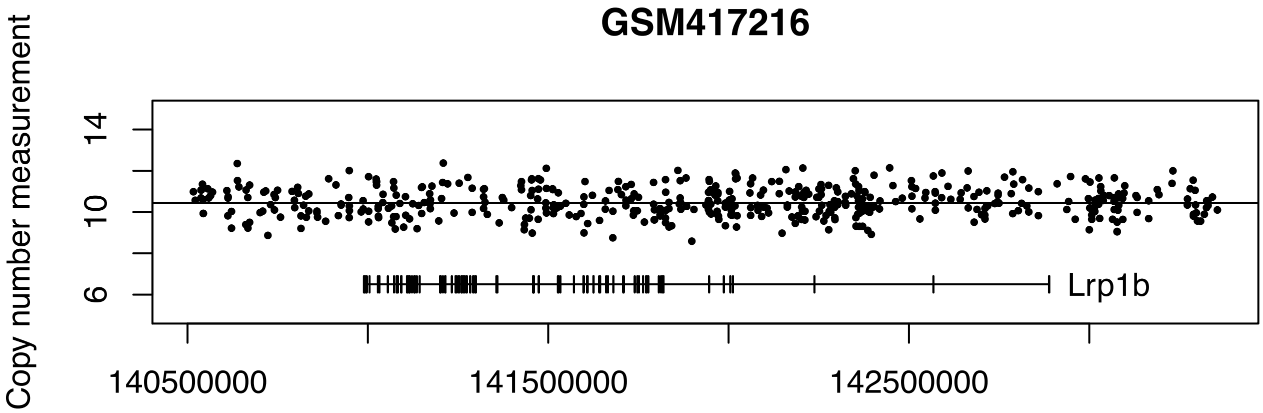

GSM417216
Copy number measurement
14
10
6
Lrp1b
140500000
141500000
142500000
Chromosome 2 – position

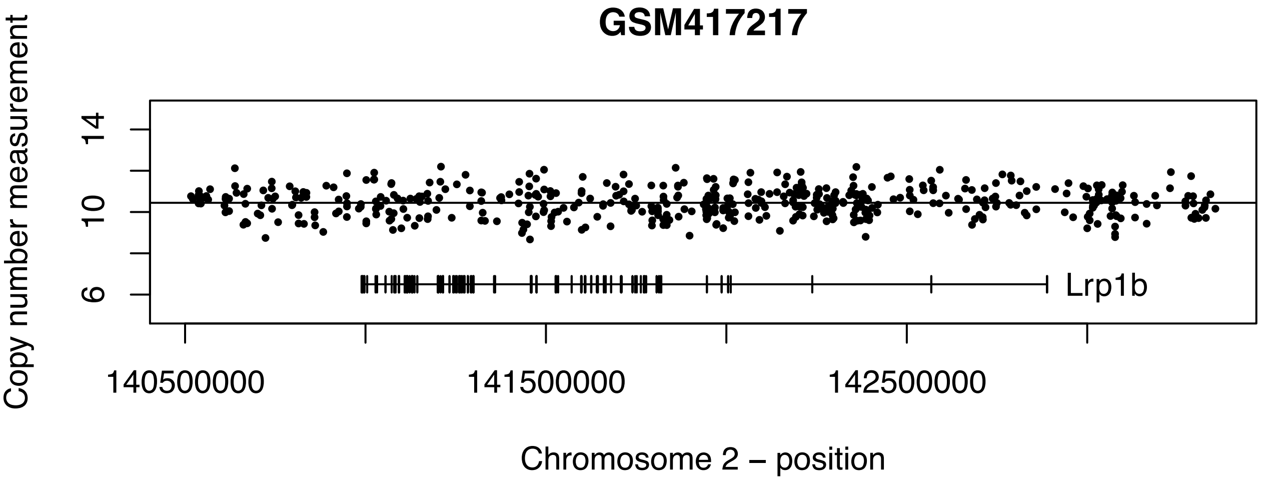

GSM417217
Copy number measurement
14
10
6
Lrp1b
140500000
141500000
142500000
Chromosome 2 – position

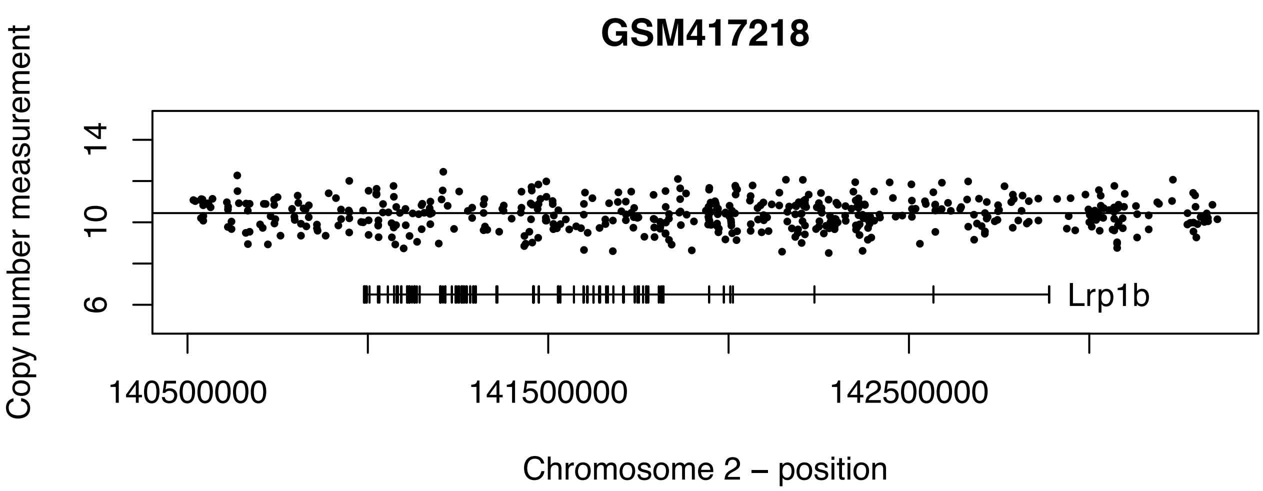
GSM417218
Copy number measurement
14
10
6
Lrp1b
140500000
141500000
142500000
Chromosome 2 – position

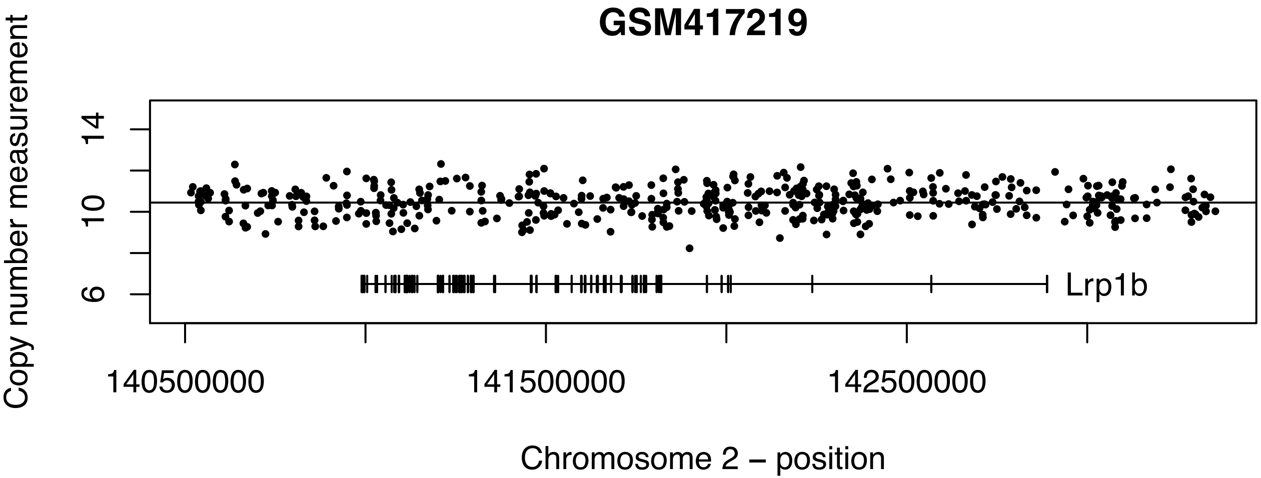
GSM417219
Copy number measurement
14
10
6
Lrp1b
140500000
141500000
142500000
Chromosome 2 – position

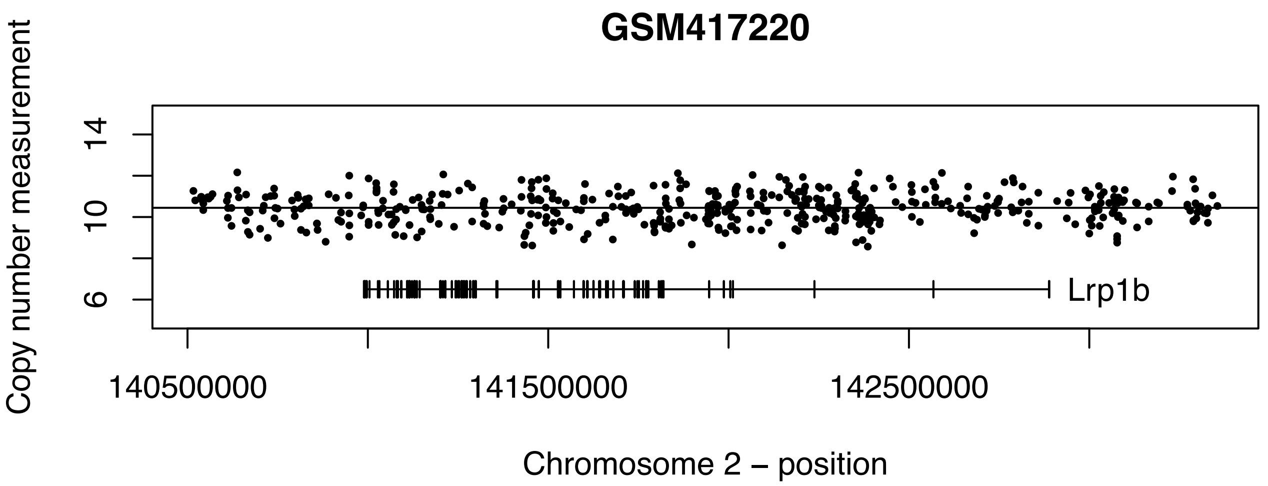

GSM417220
Copy number measurement
14
10
6
Lrp1b
140500000
141500000
142500000
Chromosome 2 – position

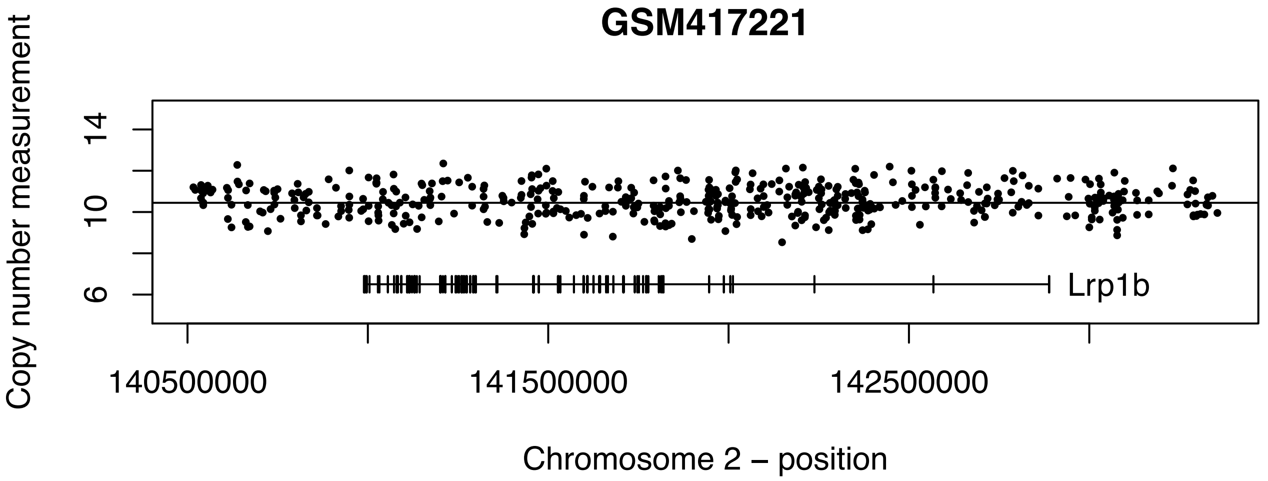
GSM417221
Copy number measurement
14
10
6
Lrp1b
140500000
141500000
142500000
Chromosome 2 – position

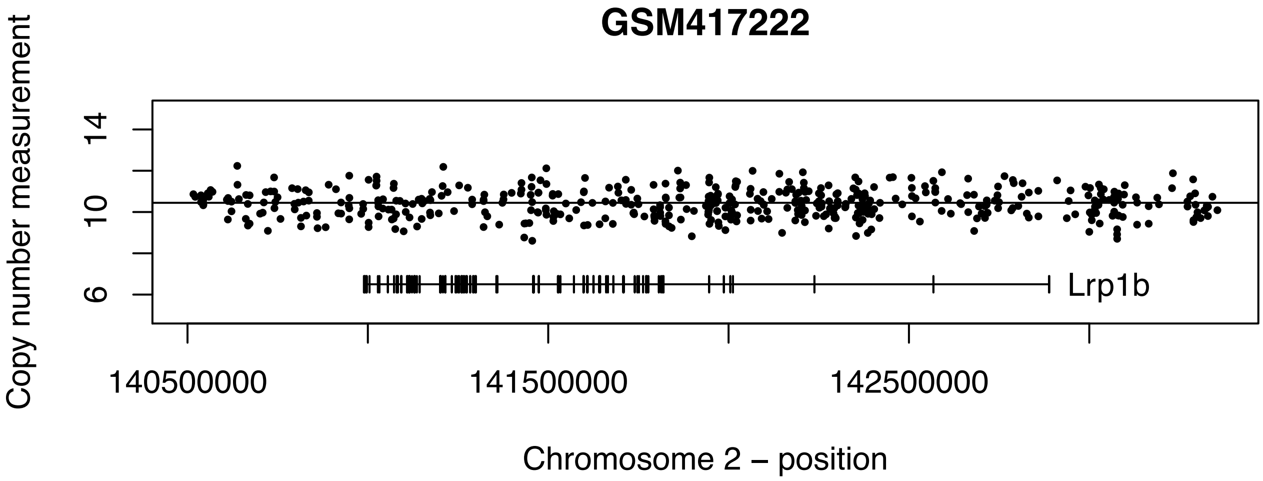
GSM417222
Copy number measurement
14
10
6
Lrp1b
140500000
141500000
142500000
Chromosome 2 – position

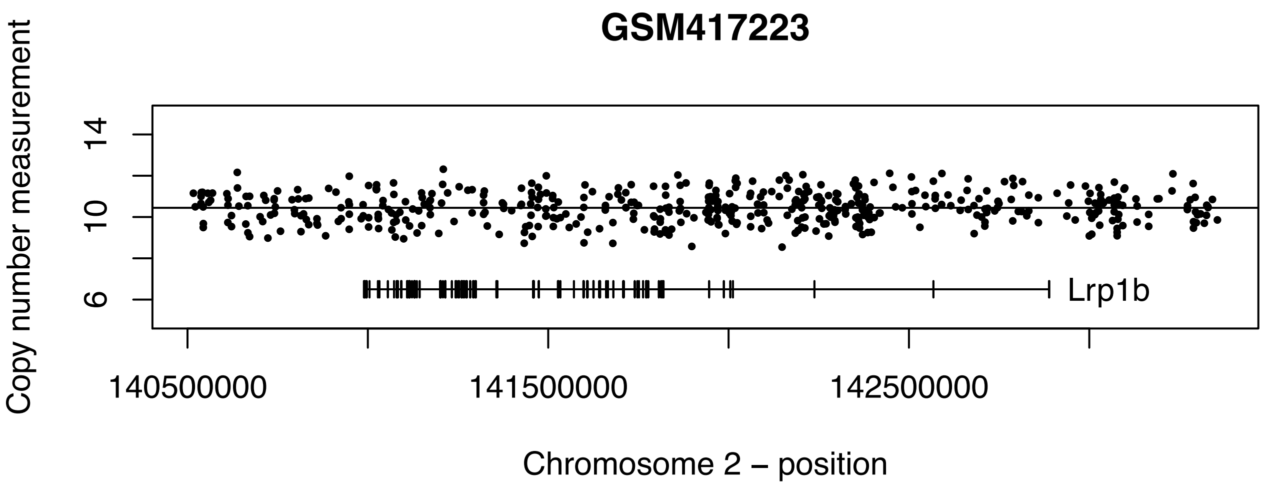
GSM417223
Copy number measurement
6
10
14
Lrp1b
140500000
141500000
142500000
Chromosome 2 – position

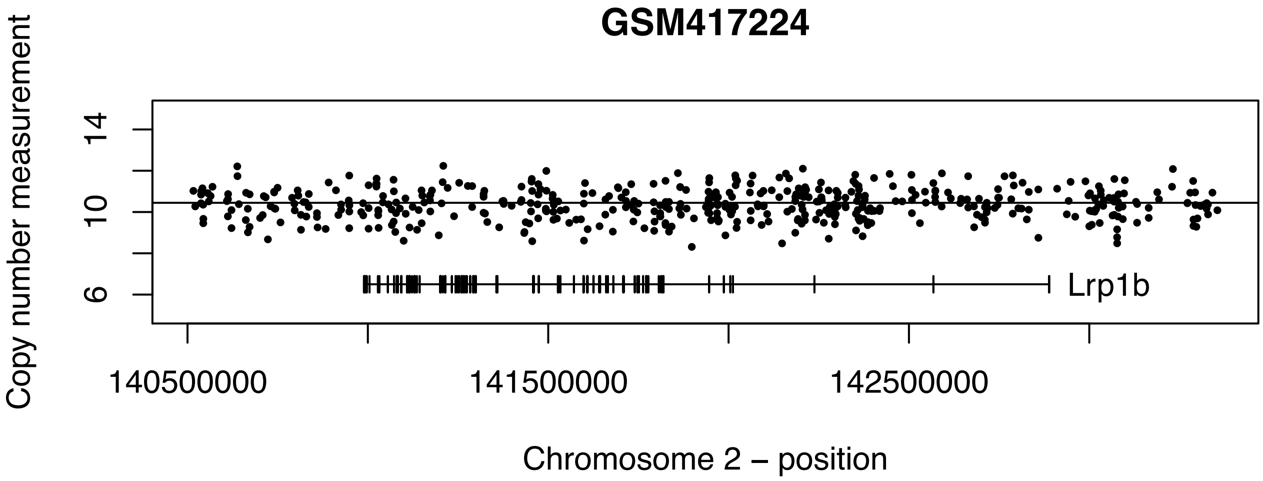

GSM417224
Copy number measurement
14
10
6
Lrp1b
140500000
141500000
142500000
Chromosome 2 – position

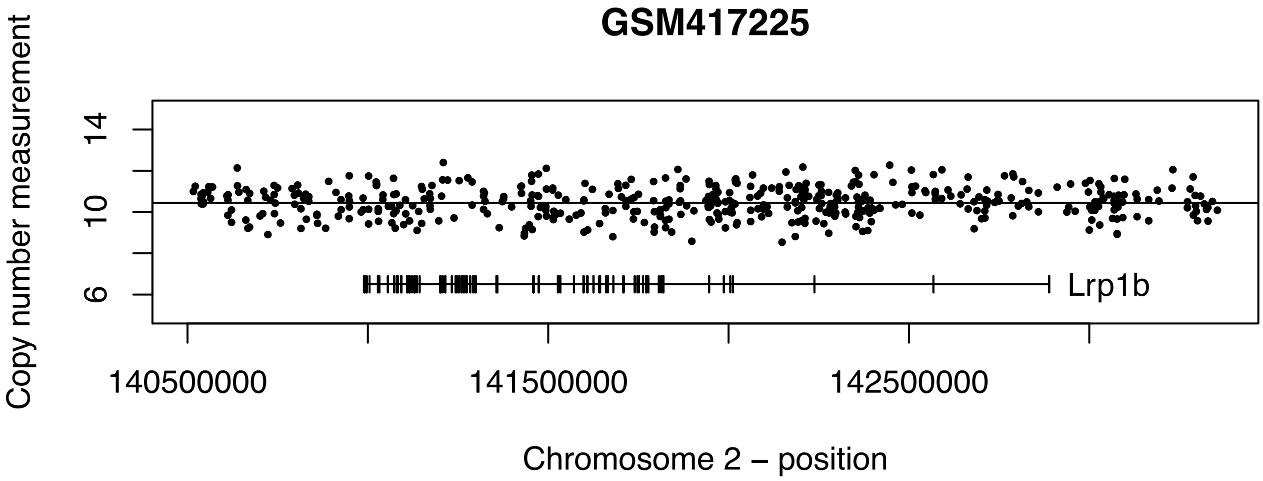
GSM417225
Copy number measurement
14
10
6
Lrp1b
140500000
141500000
142500000
Chromosome 2 – position

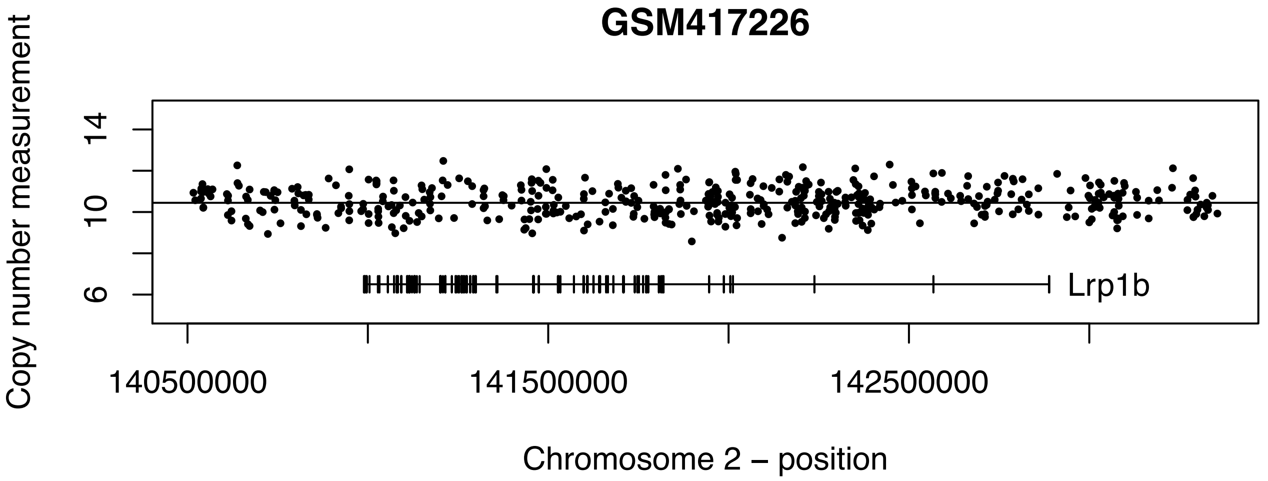

GSM417226
Copy number measurement
14
10
6
Lrp1b
140500000
141500000
142500000
Chromosome 2 – position

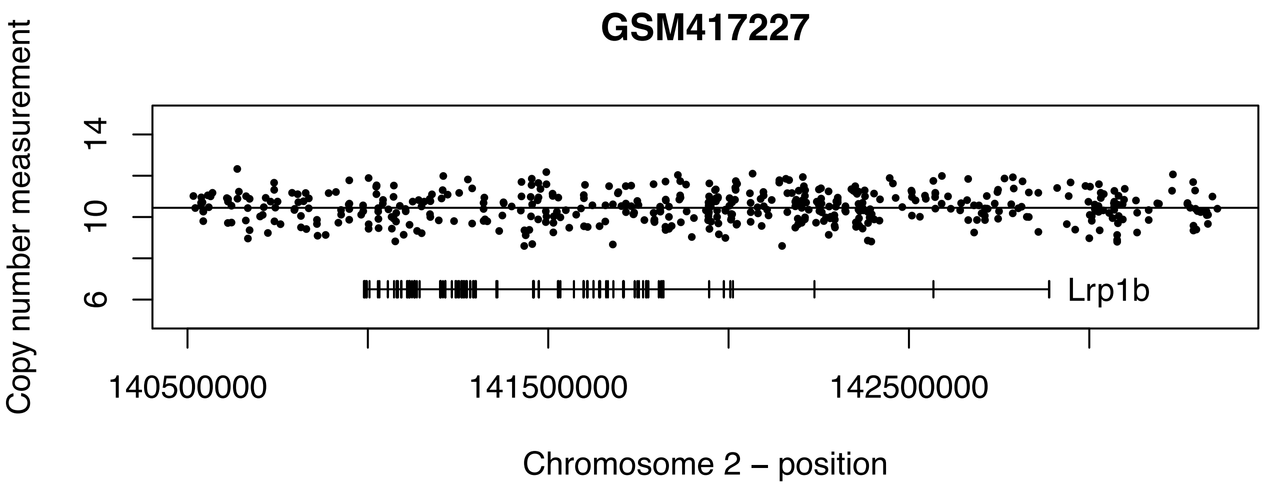
GSM417227
Copy number measurement
14
10
6
Lrp1b
140500000
141500000
142500000
Chromosome 2 – position

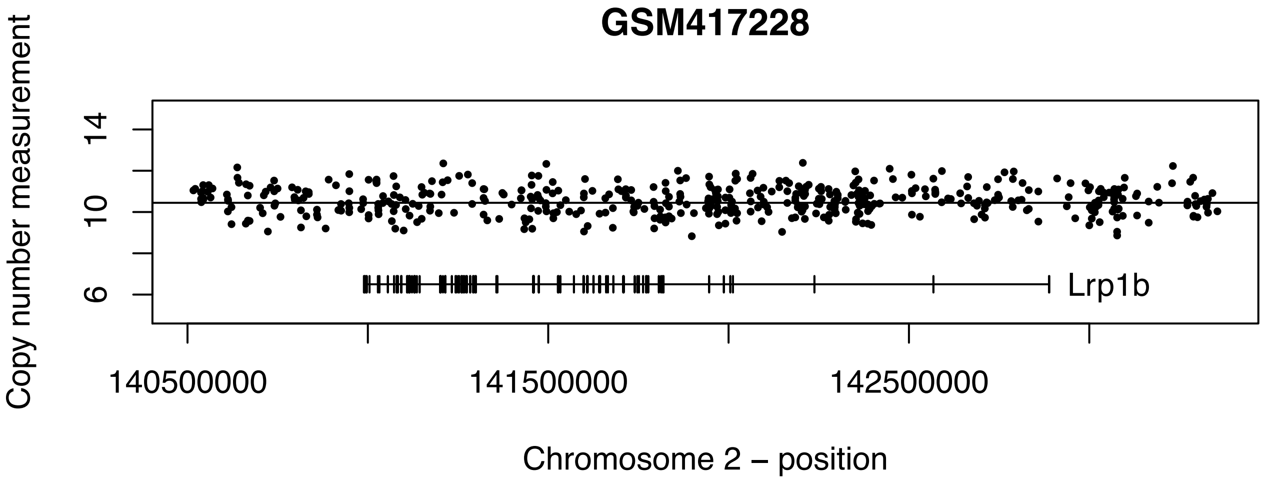

GSM417228
Copy number measurement
14
10
6
Lrp1b
140500000
141500000
142500000
Chromosome 2 – position

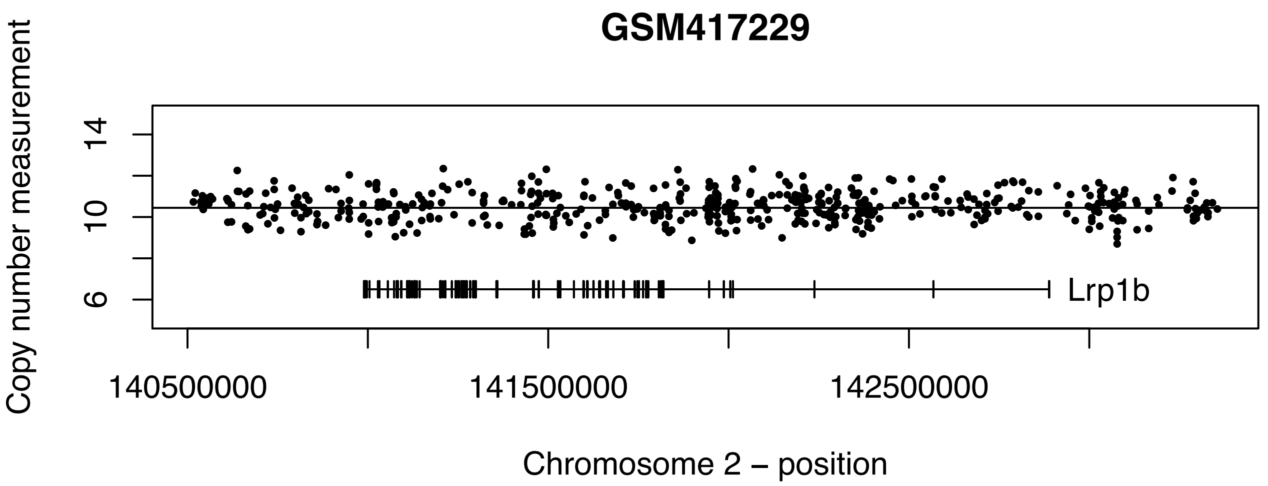
GSM417229
Copy number measurement
14
10
6
Lrp1b
140500000
141500000
142500000
Chromosome 2 – position

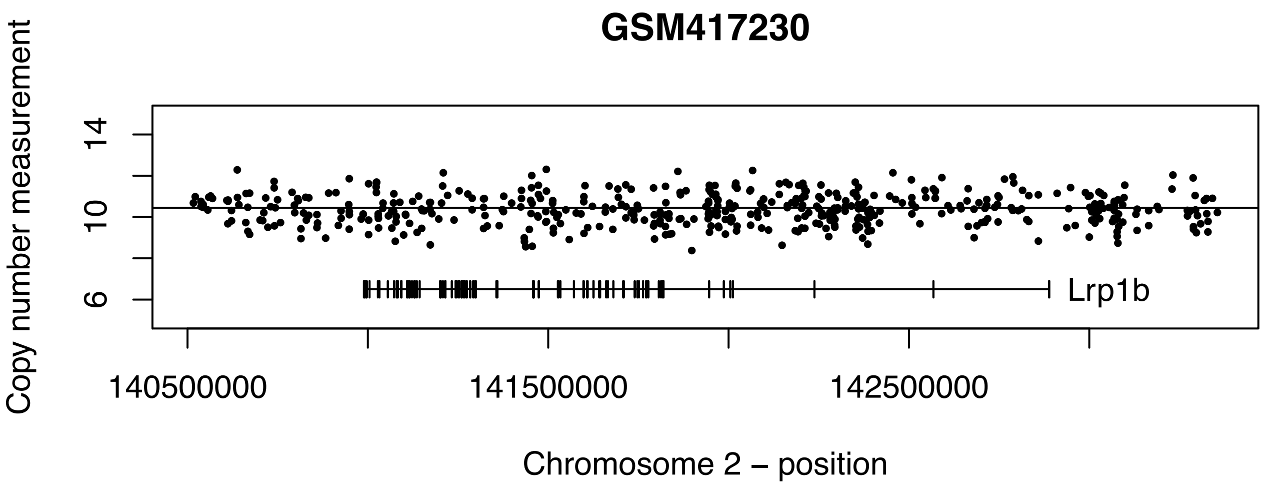

GSM417230
Copy number measurement
14
10
6
Lrp1b
140500000
141500000
142500000
Chromosome 2 – position

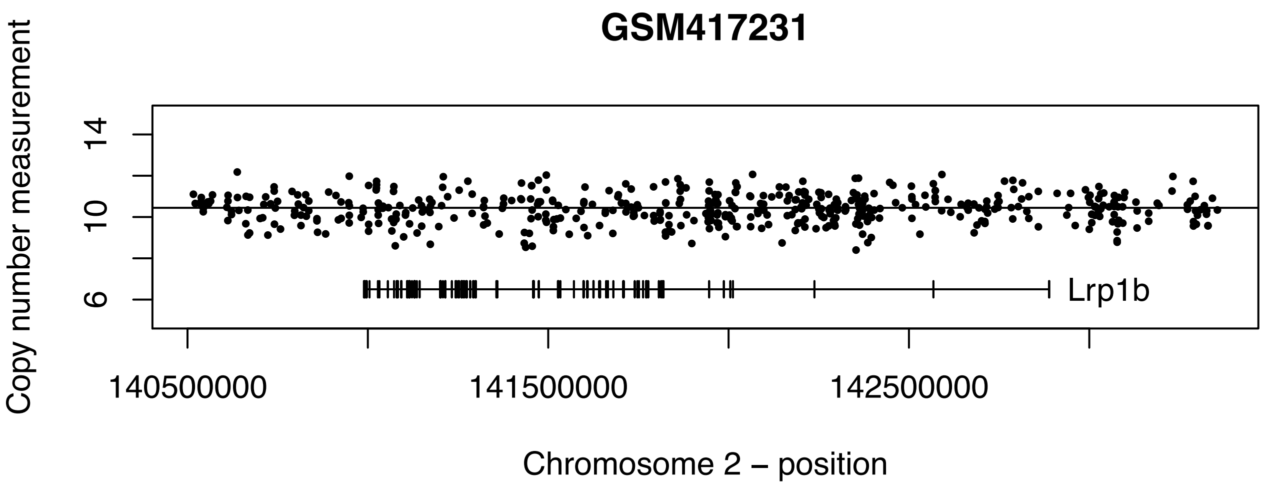
GSM417231
Copy number measurement
14
10
6
Lrp1b
140500000
141500000
142500000
Chromosome 2 – position

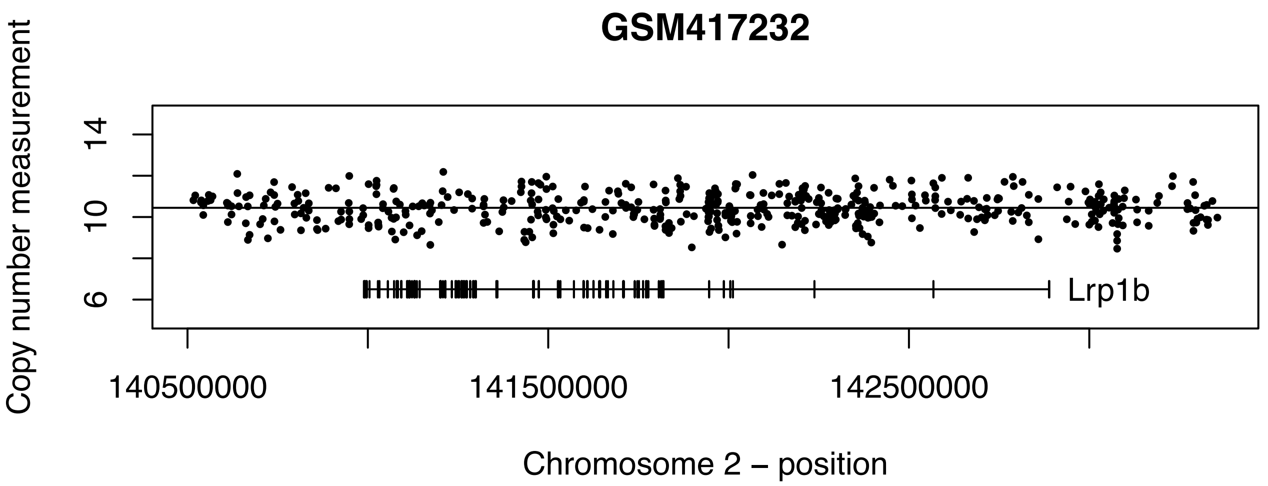
GSM417232
Copy number measurement
14
10
6
Lrp1b
140500000
141500000
142500000
Chromosome 2 – position

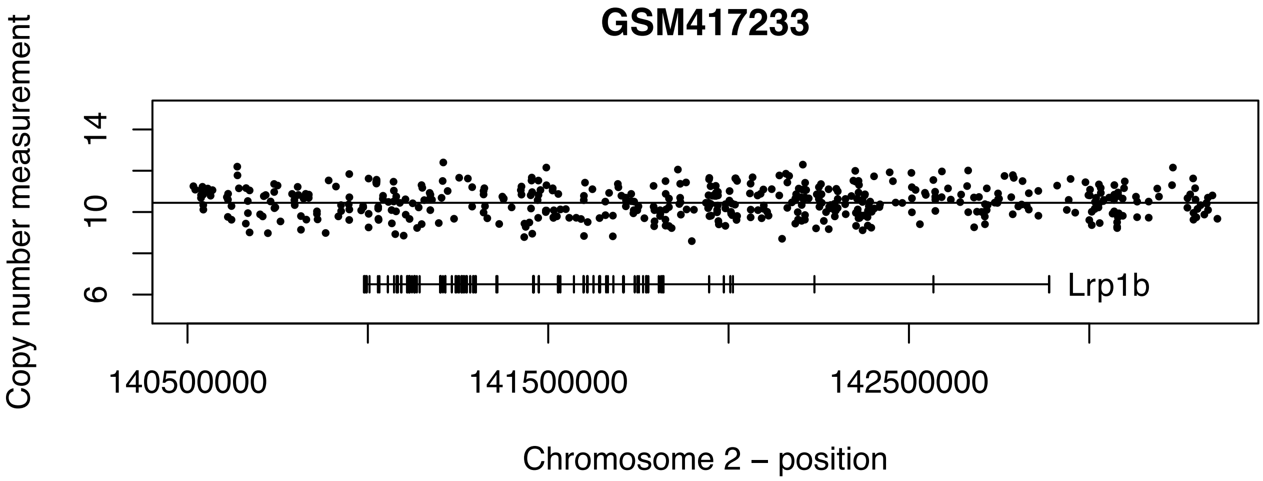

GSM417233
Copy number measurement
14
10
6
Lrp1b
140500000
141500000
142500000
Chromosome 2 – position

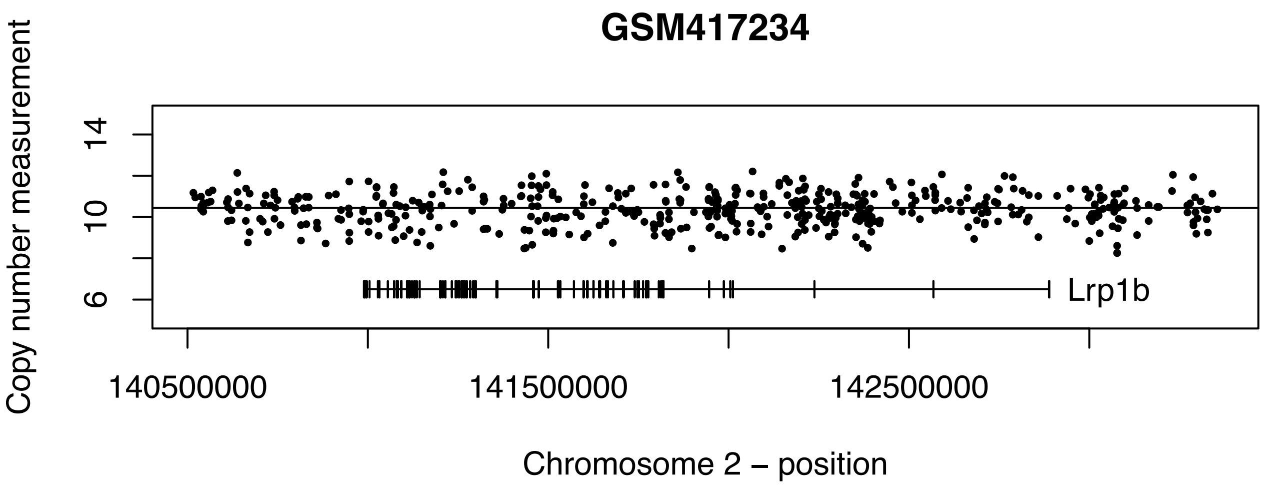
GSM417234
Copy number measurement
6
10
14
Lrp1b
140500000
141500000
142500000
Chromosome 2 – position

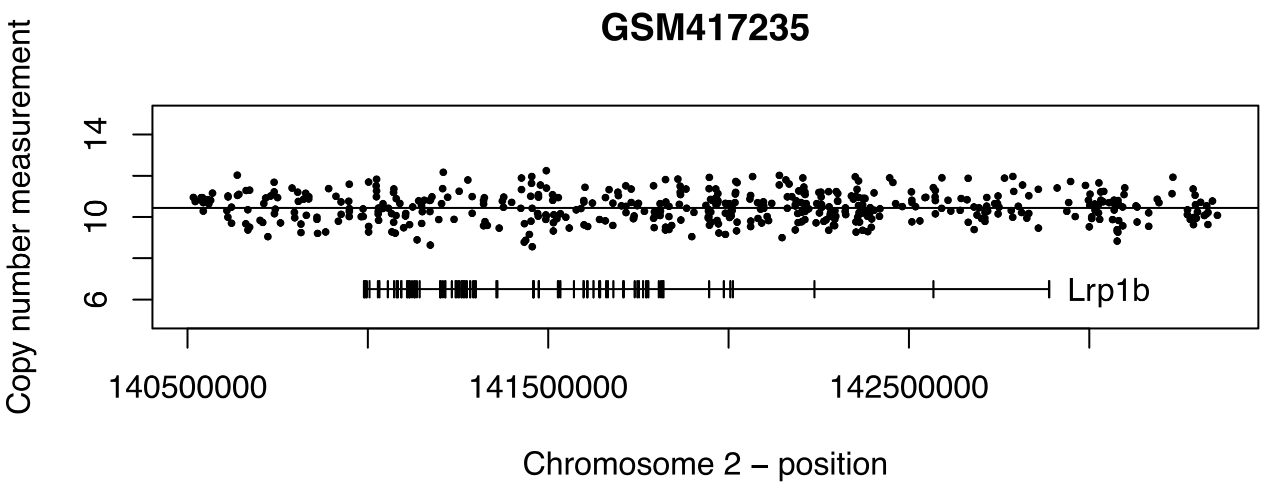

GSM417235
Copy number measurement
14
10
6
Lrp1b
140500000
141500000
142500000
Chromosome 2 – position

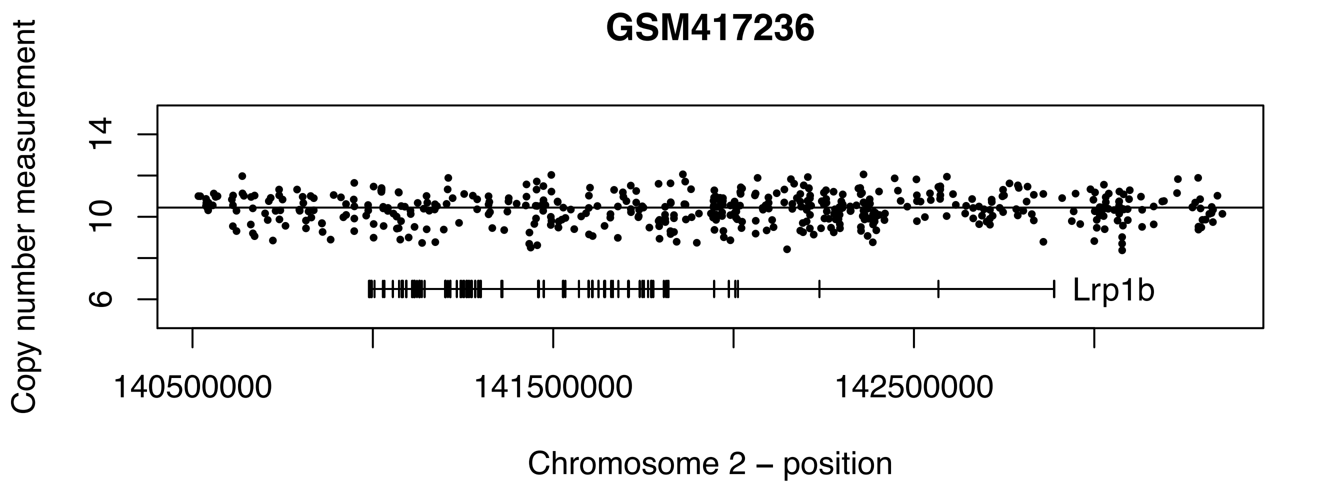
GSM417236
Copy number measurement
14
10
6
Lrp1b
140500000
141500000
142500000
Chromosome 2 – position

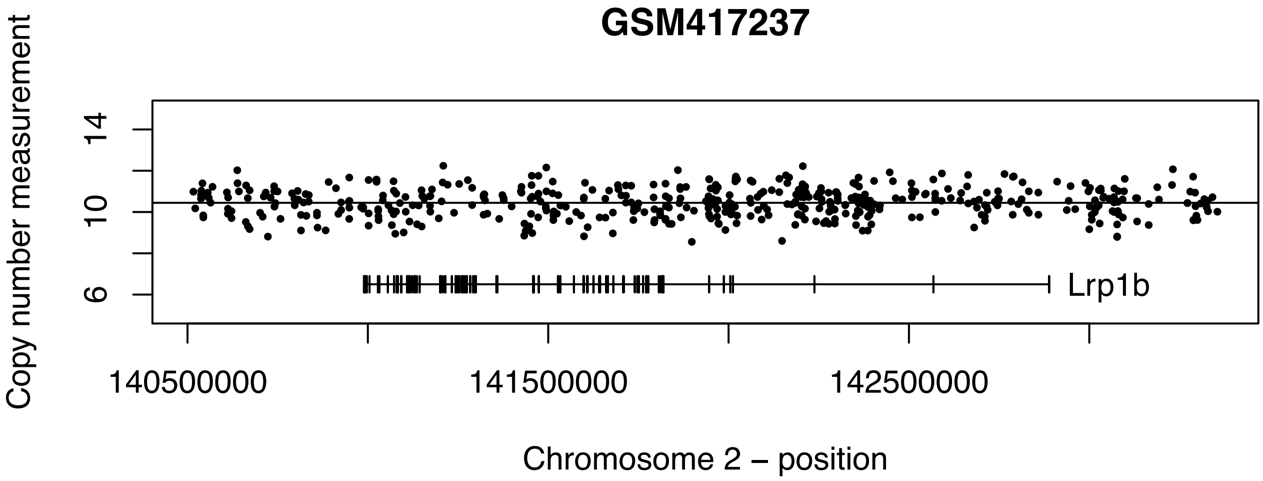
GSM417237
Copy number measurement
14
10
6
Lrp1b
140500000
141500000
142500000
Chromosome 2 – position

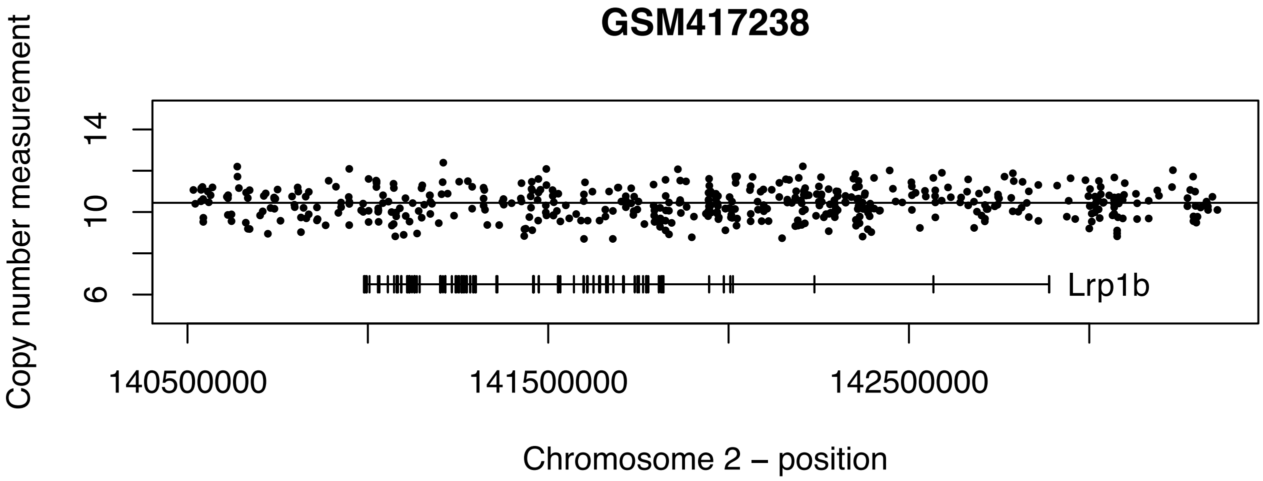
GSM417238
Copy number measurement
14
10
6
Lrp1b
140500000
141500000
142500000
Chromosome 2 – position

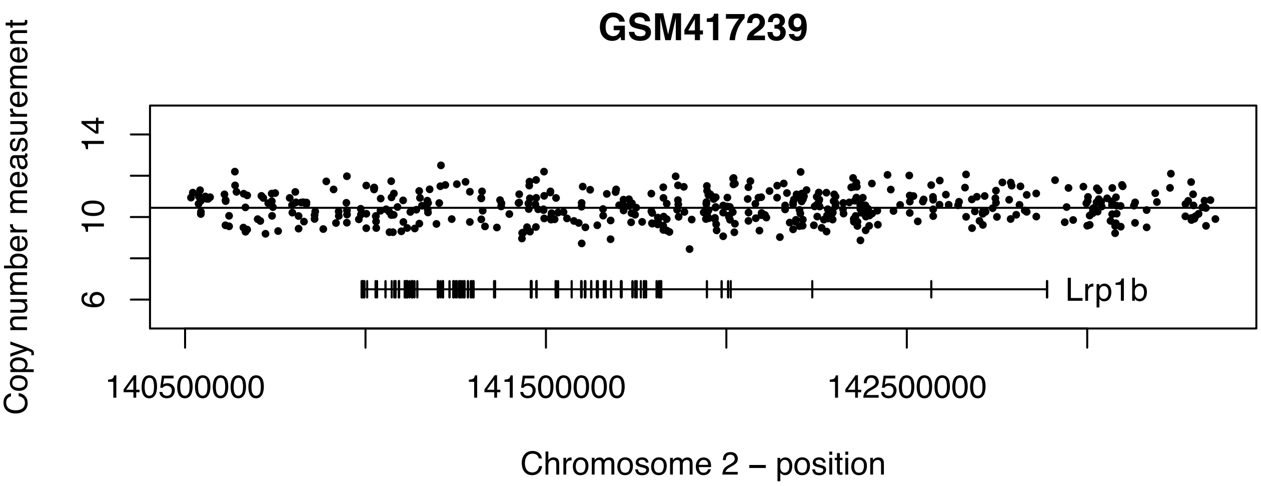

GSM417239
Copy number measurement
14
10
6
Lrp1b
140500000
141500000
142500000
Chromosome 2 – position

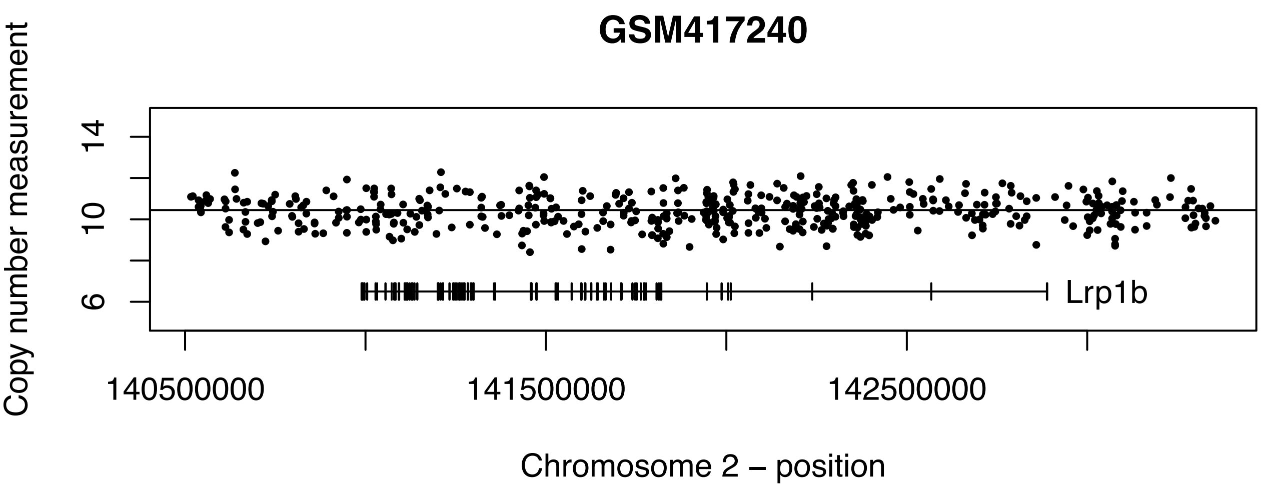
GSM417240
Copy number measurement
14
10
6
Lrp1b
140500000
141500000
142500000
Chromosome 2 – position

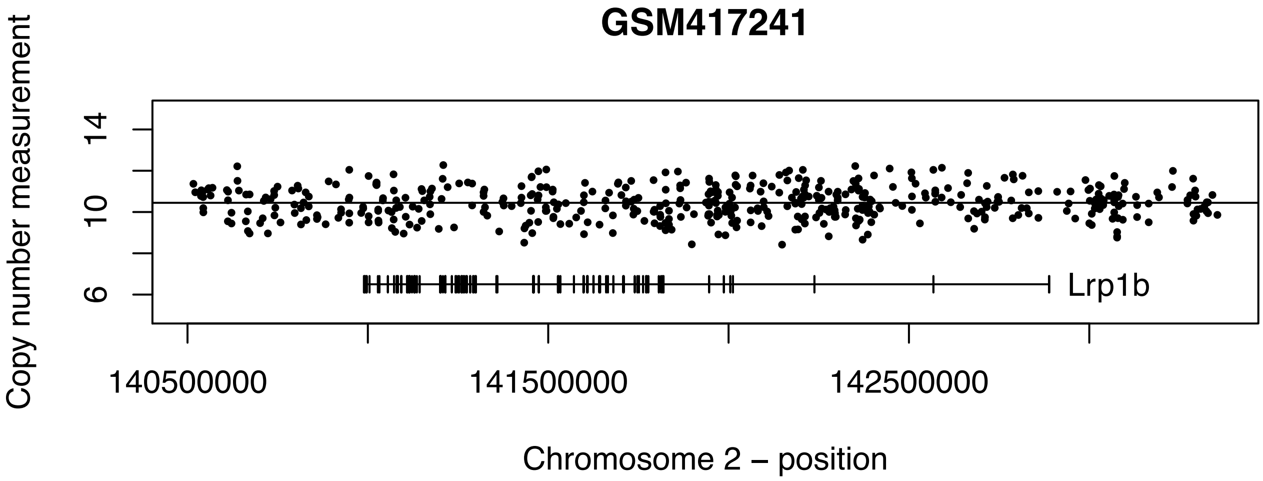

GSM417241
Copy number measurement
14
10
6
Lrp1b
140500000
141500000
142500000
Chromosome 2 – position

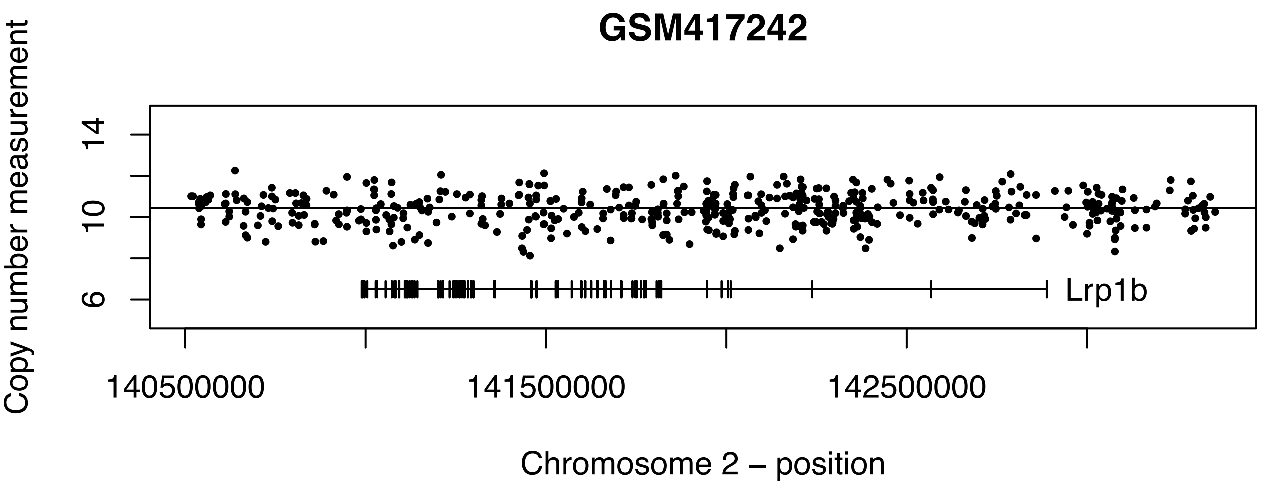

GSM417242
Copy number measurement
14
10
6
Lrp1b
140500000
141500000
142500000
Chromosome 2 – position

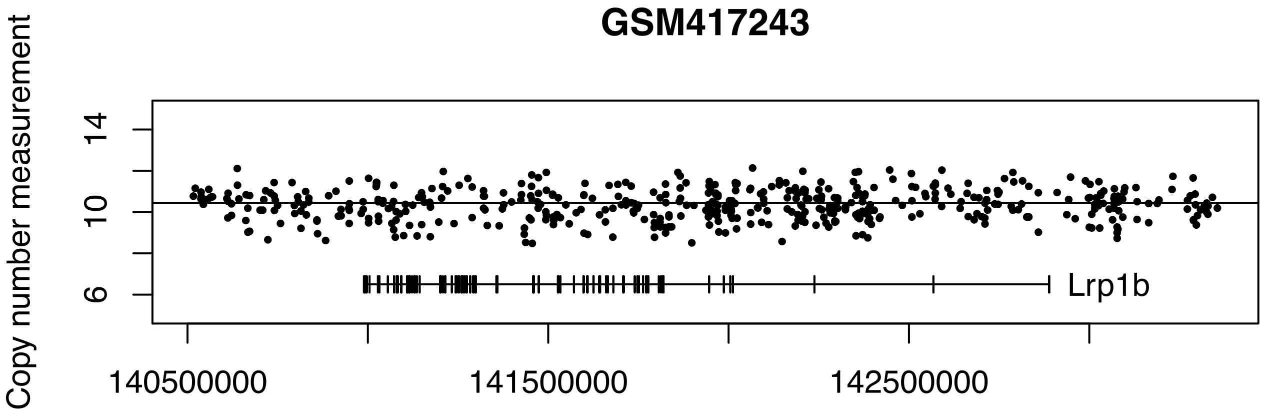

GSM417243
Copy number measurement
14
10
6
Lrp1b
140500000
141500000
142500000
Chromosome 2 – position

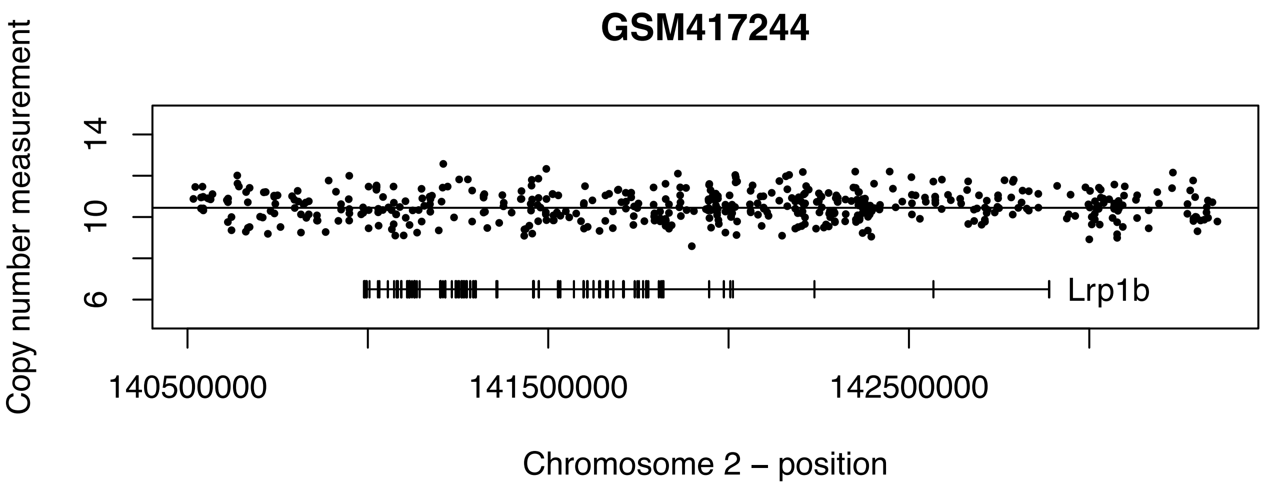
GSM417244
Copy number measurement
14
10
6
Lrp1b
140500000
141500000
142500000
Chromosome 2 – position

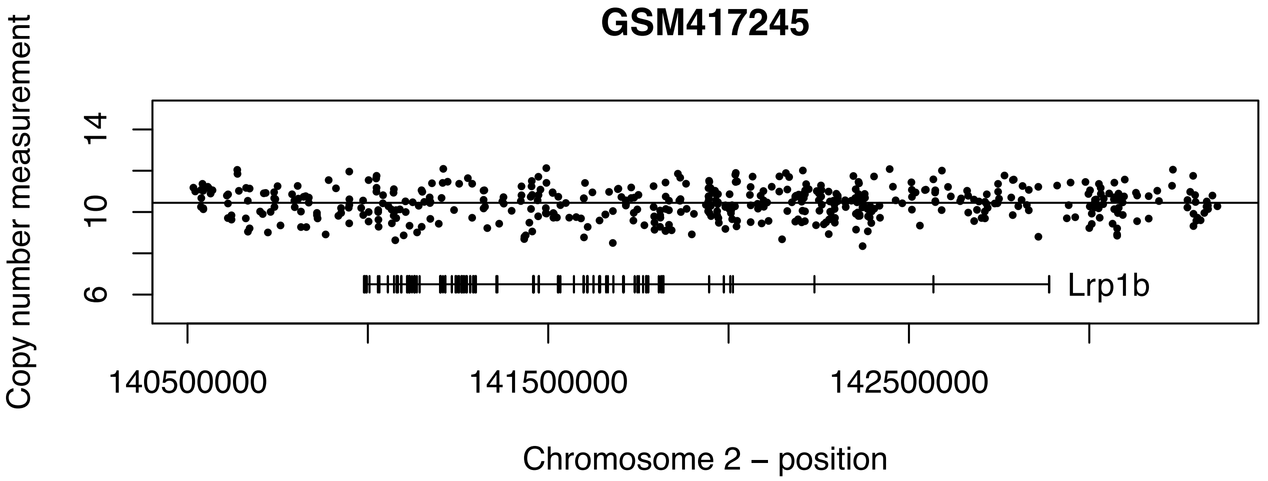
GSM417245
Copy number measurement
14
10
6
Lrp1b
140500000
141500000
142500000
Chromosome 2 – position

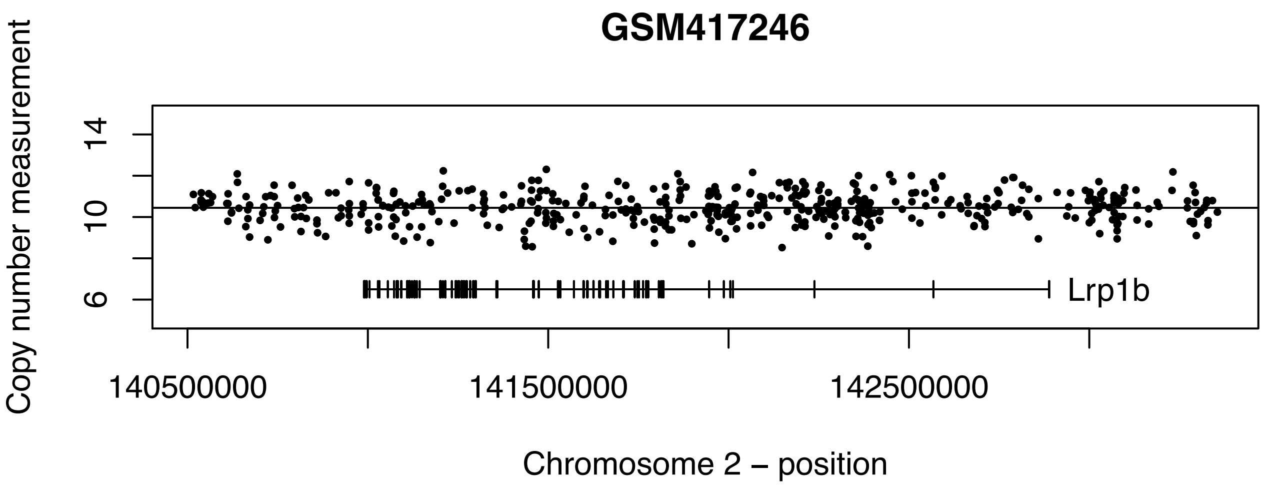

GSM417246
Copy number measurement
6
10
14
Lrp1b
140500000
141500000
142500000
Chromosome 2 – position

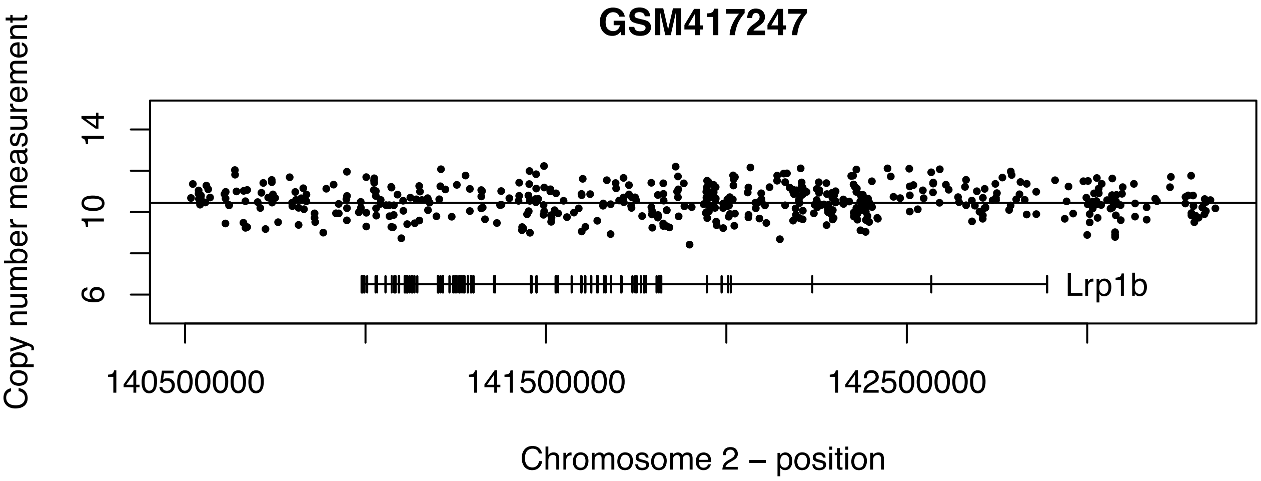
GSM417247
Copy number measurement
14
10
6
Lrp1b
140500000
141500000
142500000
Chromosome 2 – position

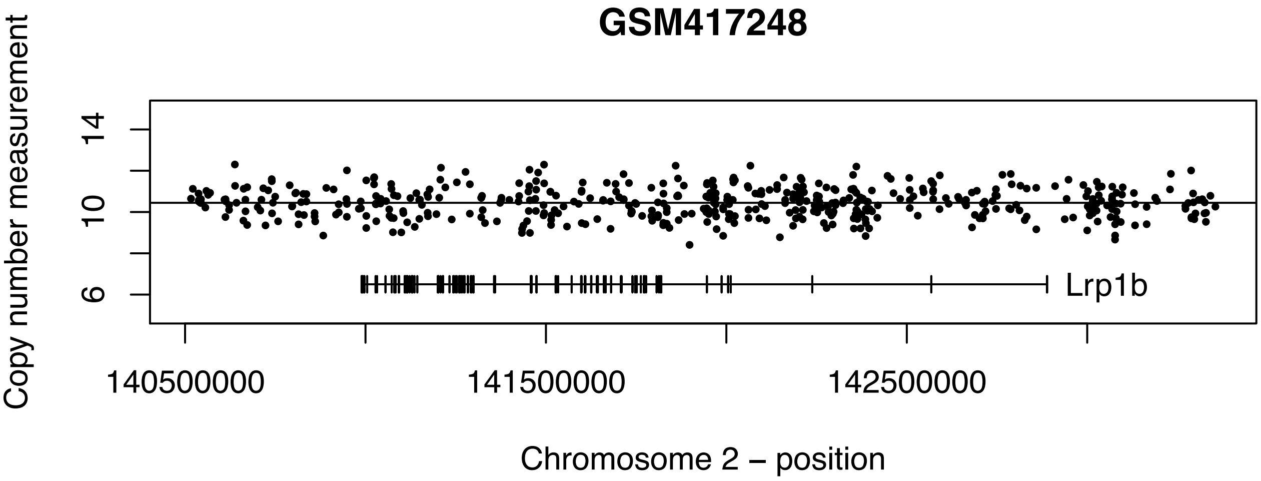
GSM417248
Copy number measurement
14
10
6
Lrp1b
140500000
141500000
142500000
Chromosome 2 – position

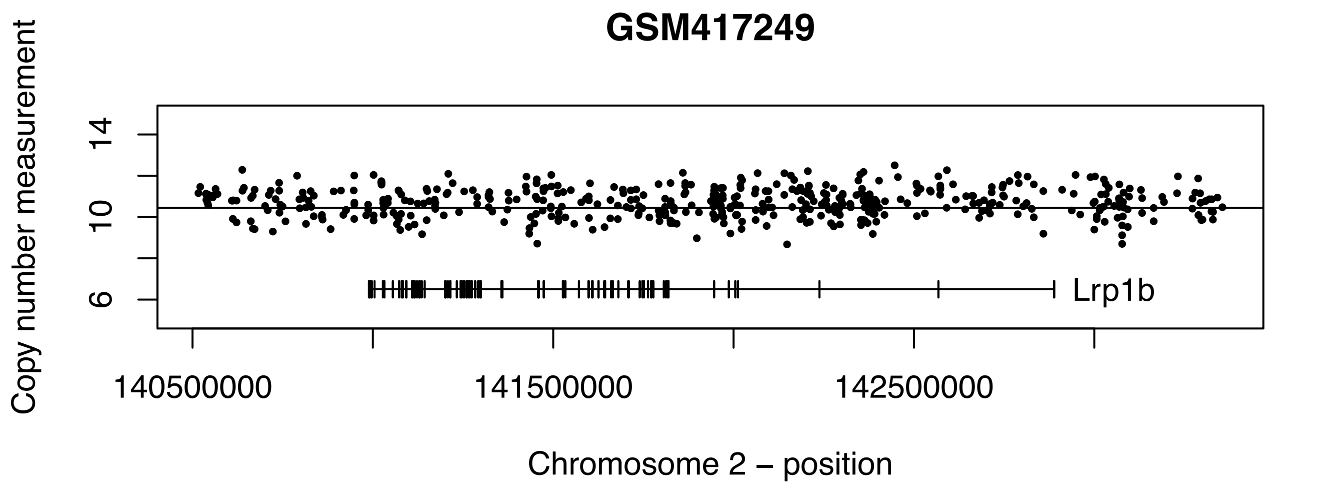

GSM417249
Copy number measurement
14
10
6
Lrp1b
140500000
141500000
142500000
Chromosome 2 – position

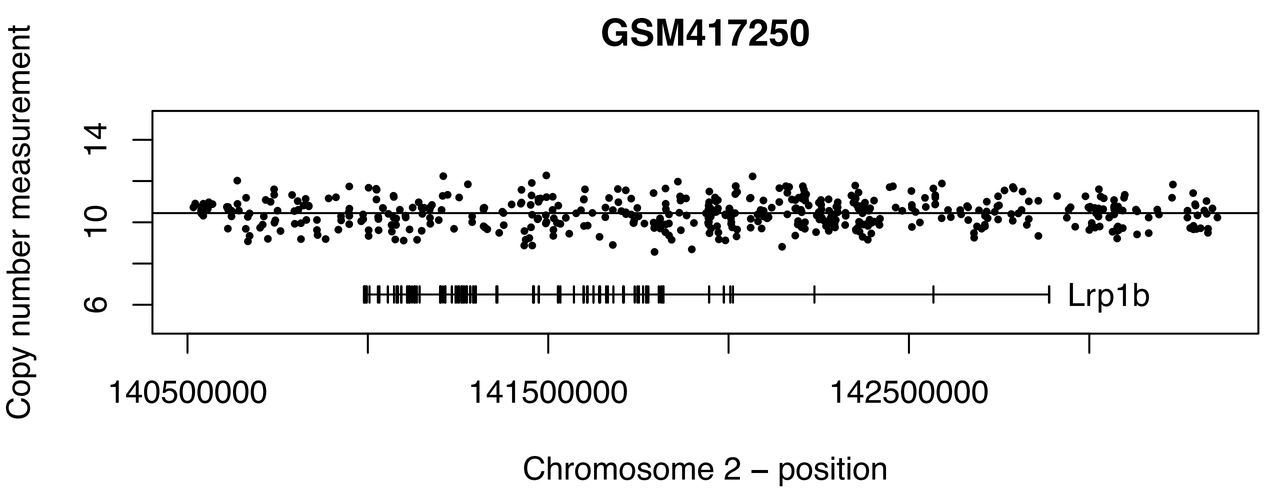
GSM417250
Copy number measurement
14
10
6
Lrp1b
140500000
141500000
142500000
Chromosome 2 – position

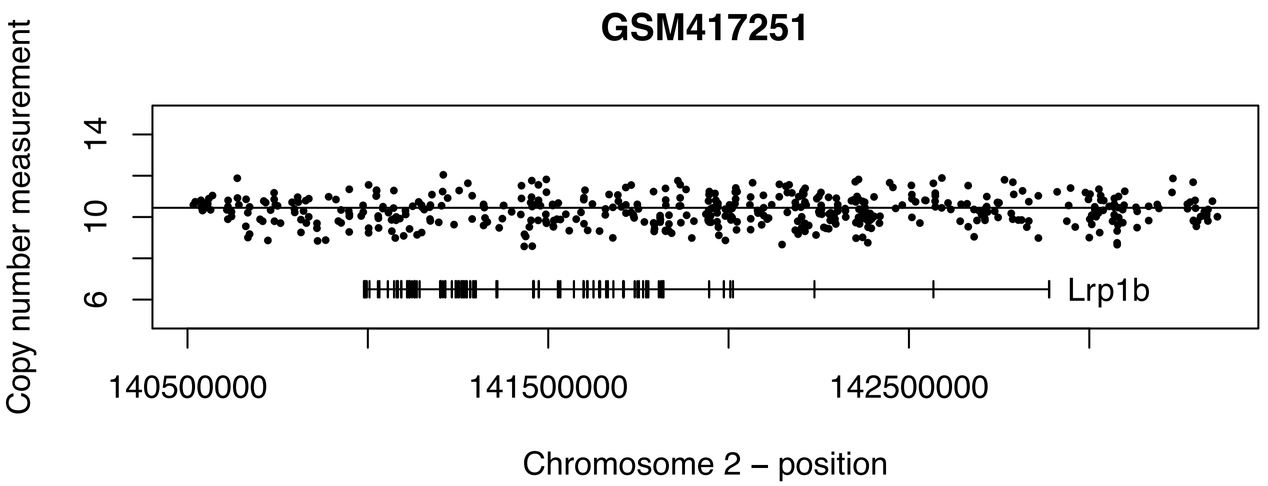
GSM417251
Copy number measurement
14
10
6
Lrp1b
140500000
141500000
142500000
Chromosome 2 – position

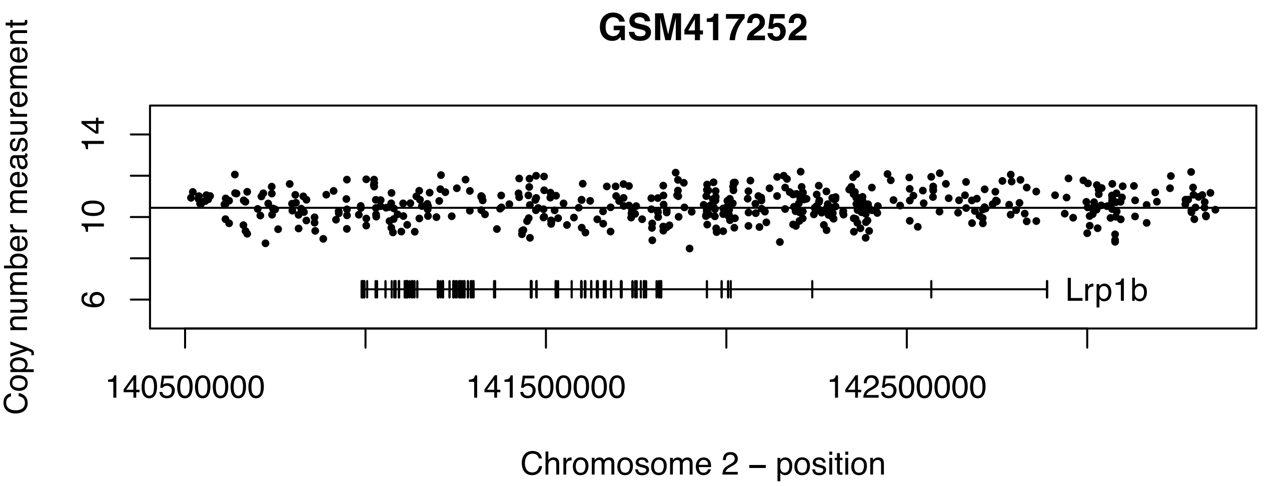

GSM417252
Copy number measurement
14
10
6
Lrp1b
140500000
141500000
142500000
Chromosome 2 – position

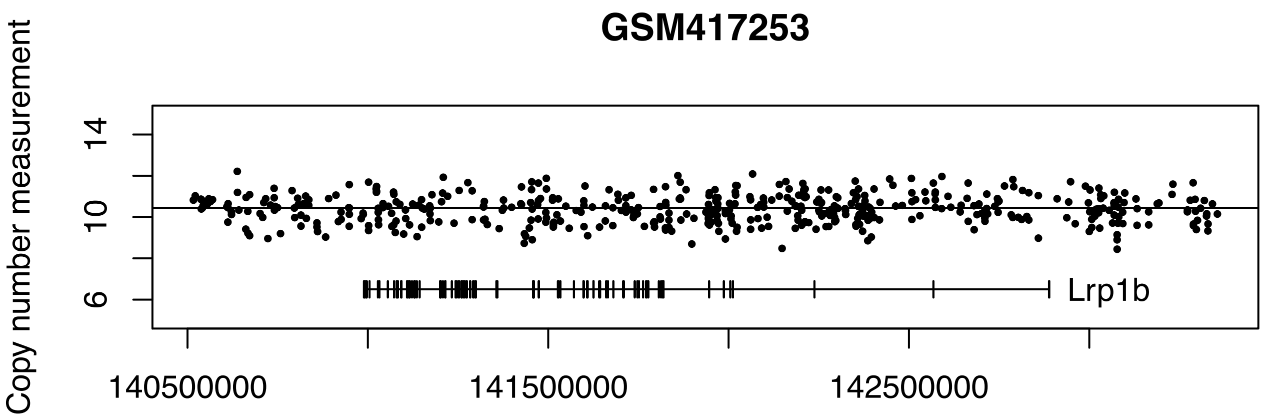
GSM417253
Copy number measurement
14
10
6
Lrp1b
140500000
141500000
142500000
Chromosome 2 – position

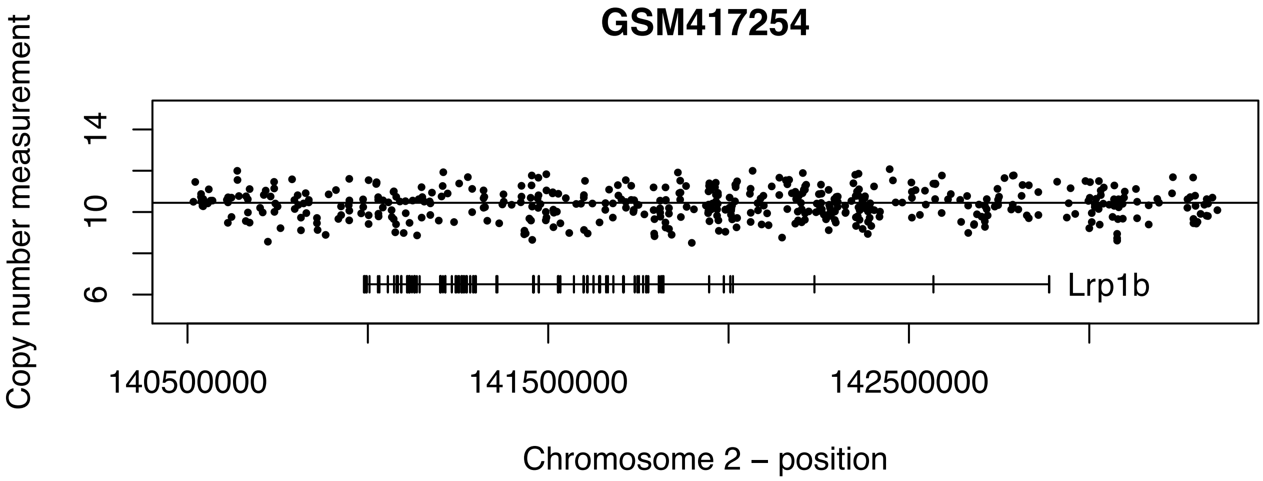

GSM417254
Copy number measurement
14
10
6
Lrp1b
140500000
141500000
142500000
Chromosome 2 – position

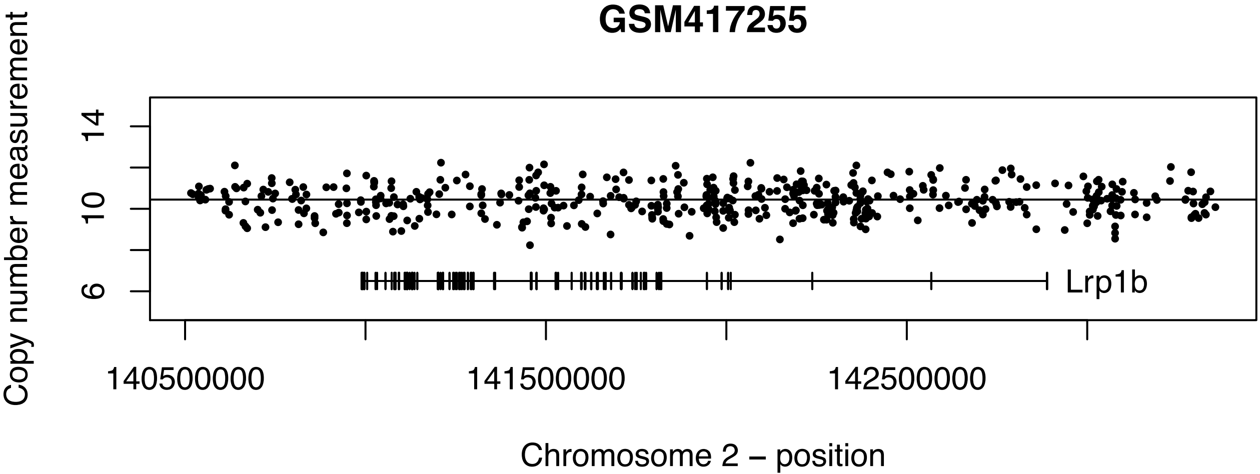
GSM417255
Copy number measurement
14
10
6
Lrp1b
140500000
141500000
142500000
Chromosome 2 – position

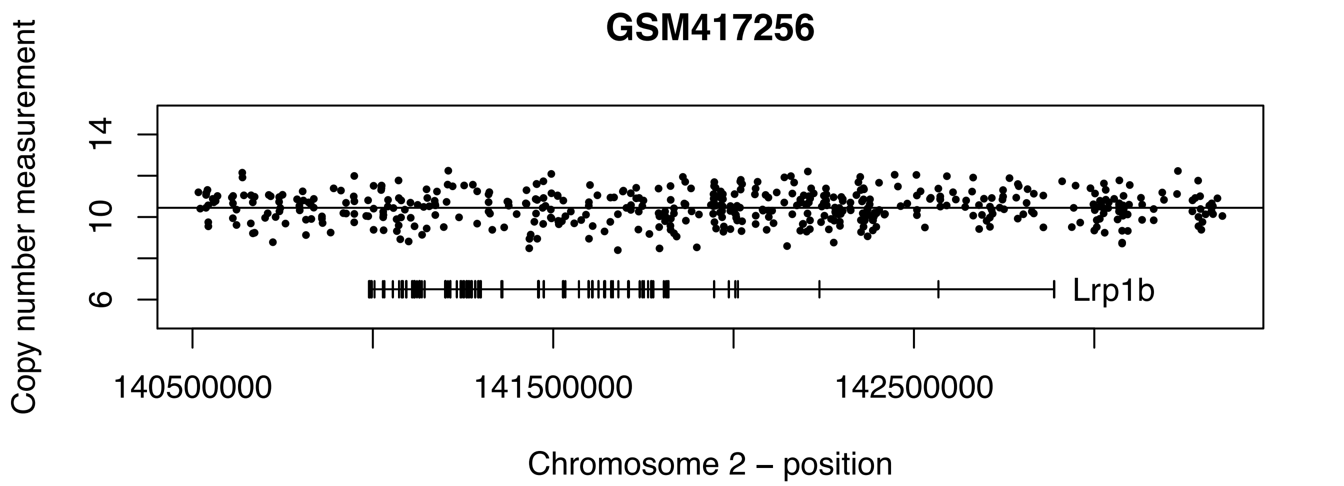
GSM417256
Copy number measurement
14
10
6
Lrp1b
140500000
141500000
142500000
Chromosome 2 – position

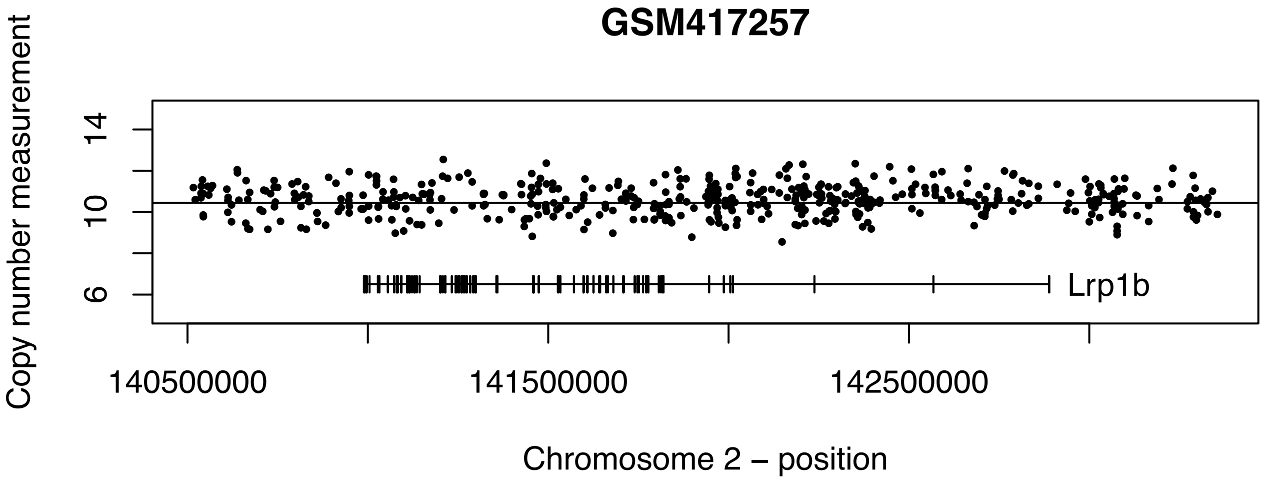

GSM417257
Copy number measurement
14
10
6
Lrp1b
140500000
141500000
142500000
Chromosome 2 – position

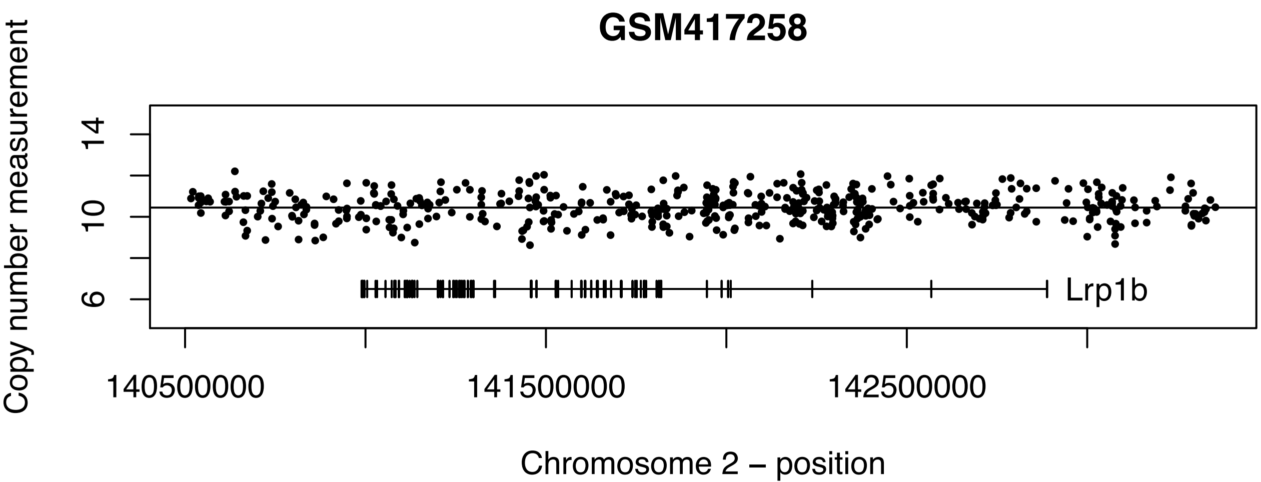

GSM417258
Copy number measurement
14
10
6
Lrp1b
140500000
141500000
142500000
Chromosome 2 – position

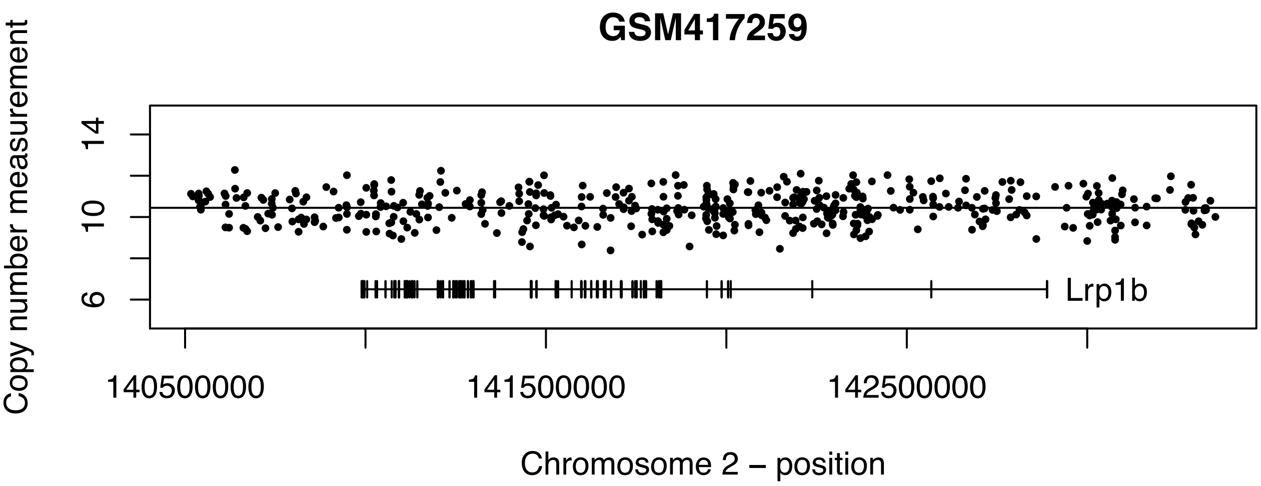

GSM417259
Copy number measurement
14
10
6
Lrp1b
140500000
141500000
142500000
Chromosome 2 – position

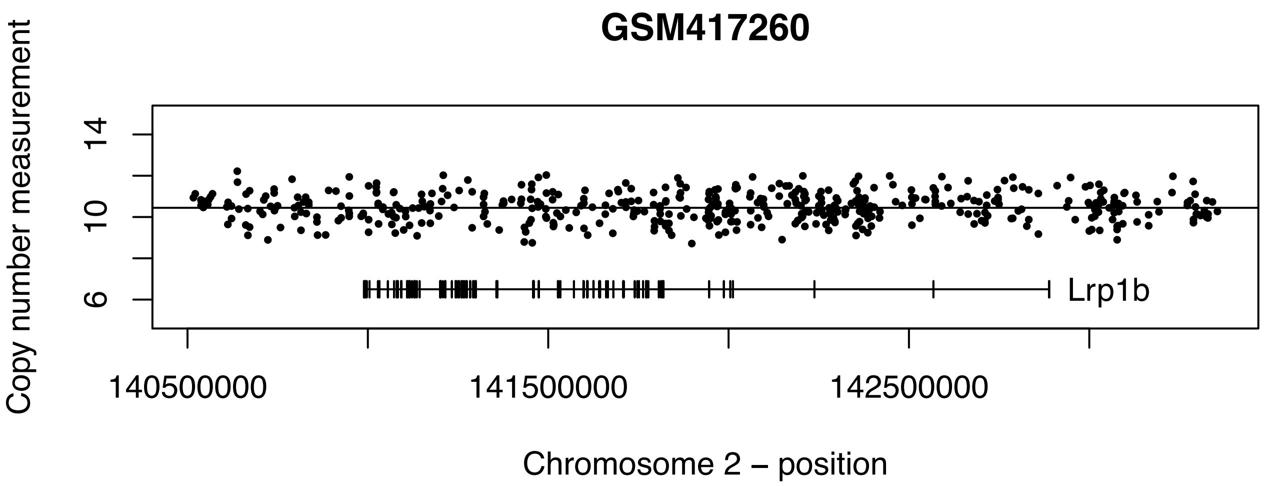
GSM417260
Copy number measurement
6
10
14
Lrp1b
140500000
141500000
142500000
Chromosome 2 – position

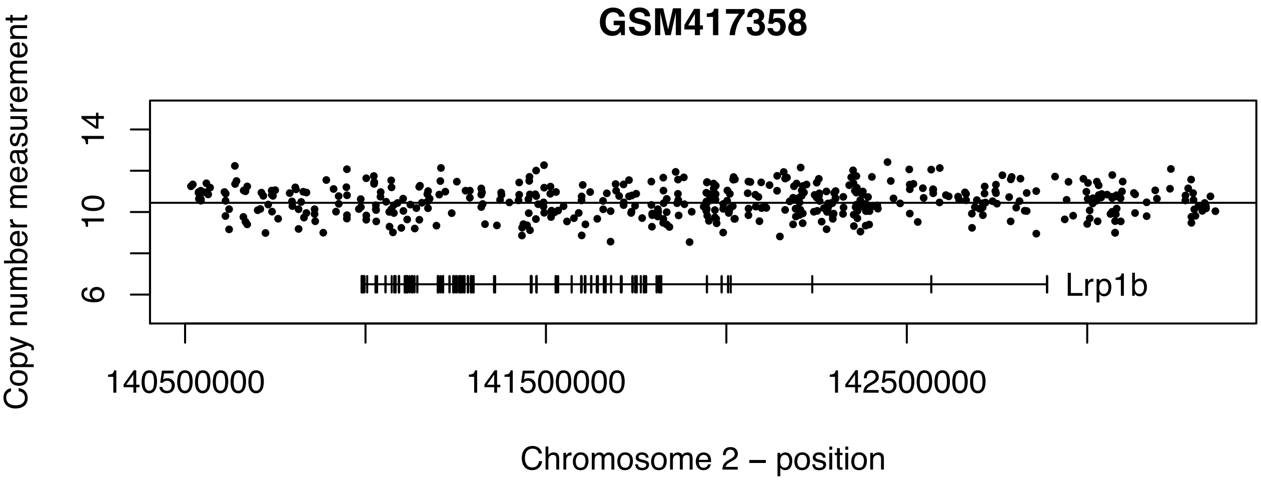

GSM417358
Copy number measurement
6
10
14
Lrp1b
140500000
141500000
142500000
Chromosome 2 – position

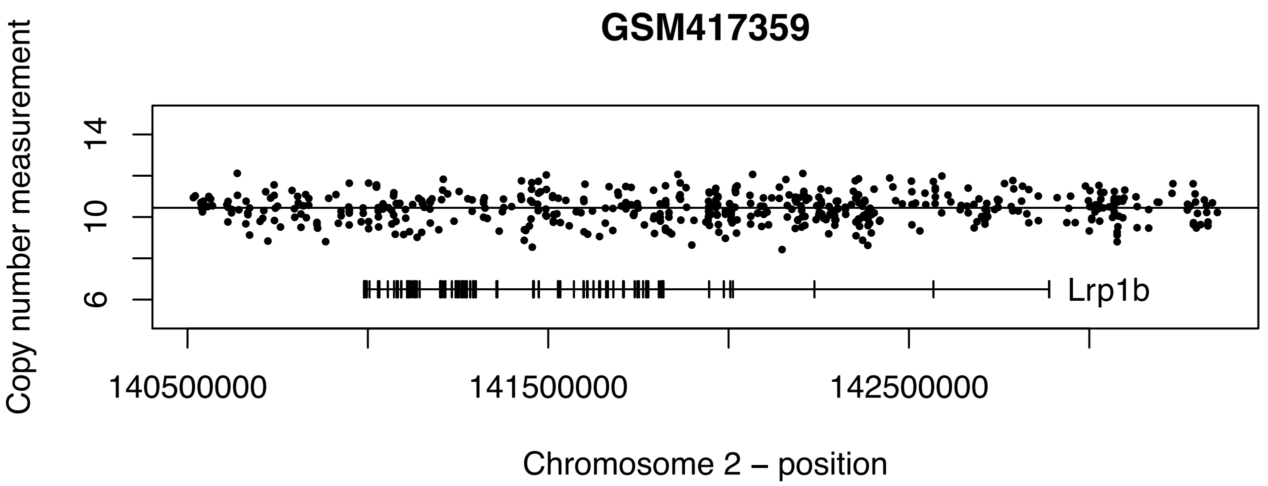
GSM417359
Copy number measurement
14
10
6
Lrp1b
140500000
141500000
142500000
Chromosome 2 – position

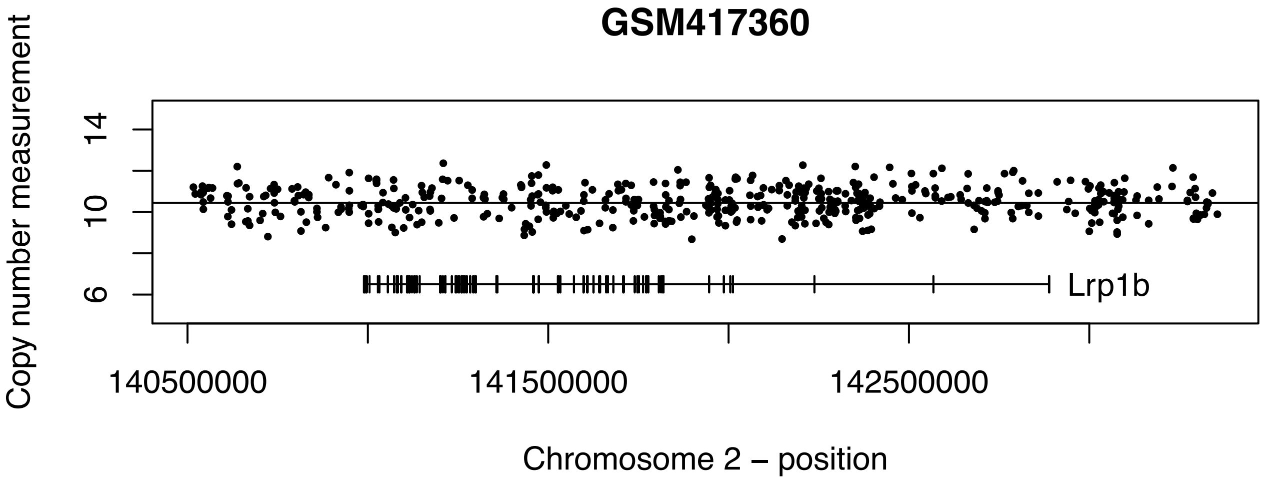
GSM417360
Copy number measurement
14
10
6
Lrp1b
140500000
141500000
142500000
Chromosome 2 – position

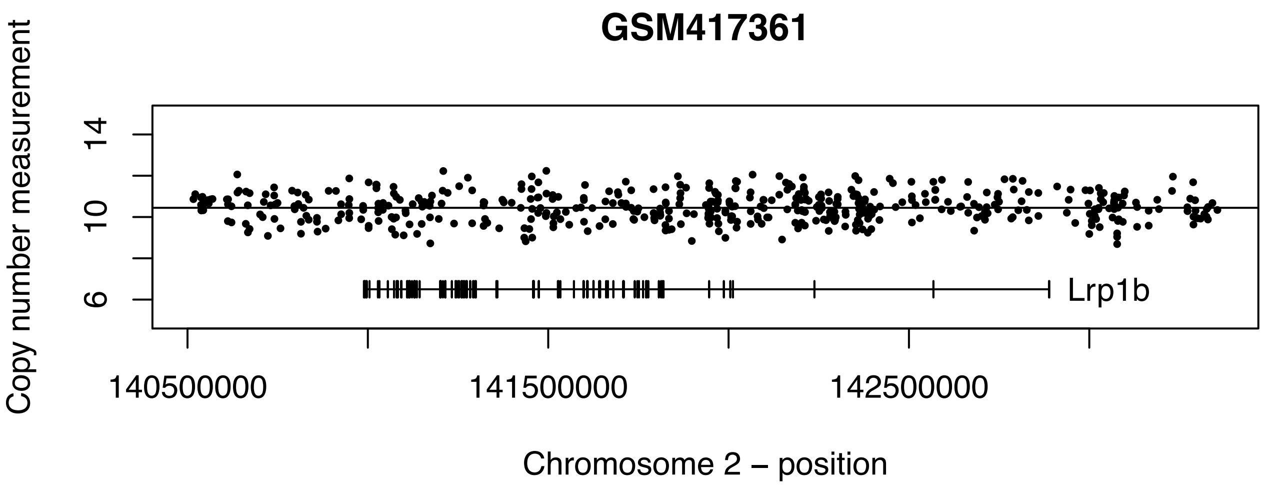
GSM417361
Copy number measurement
14
10
6
Lrp1b
140500000
141500000
142500000
Chromosome 2 – position

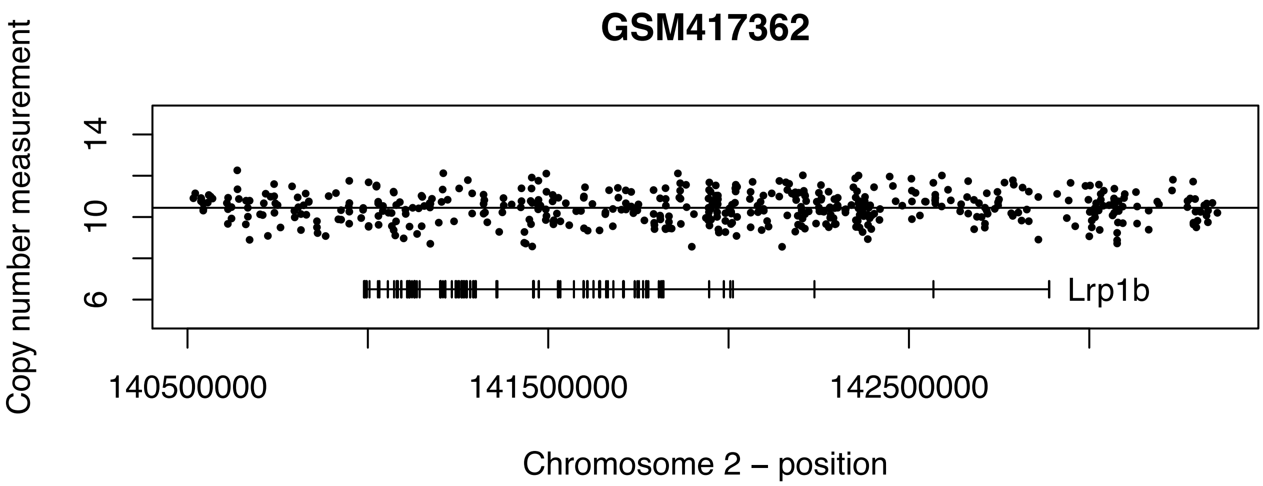

GSM417362
Copy number measurement
14
10
6
Lrp1b
140500000
141500000
142500000
Chromosome 2 – position

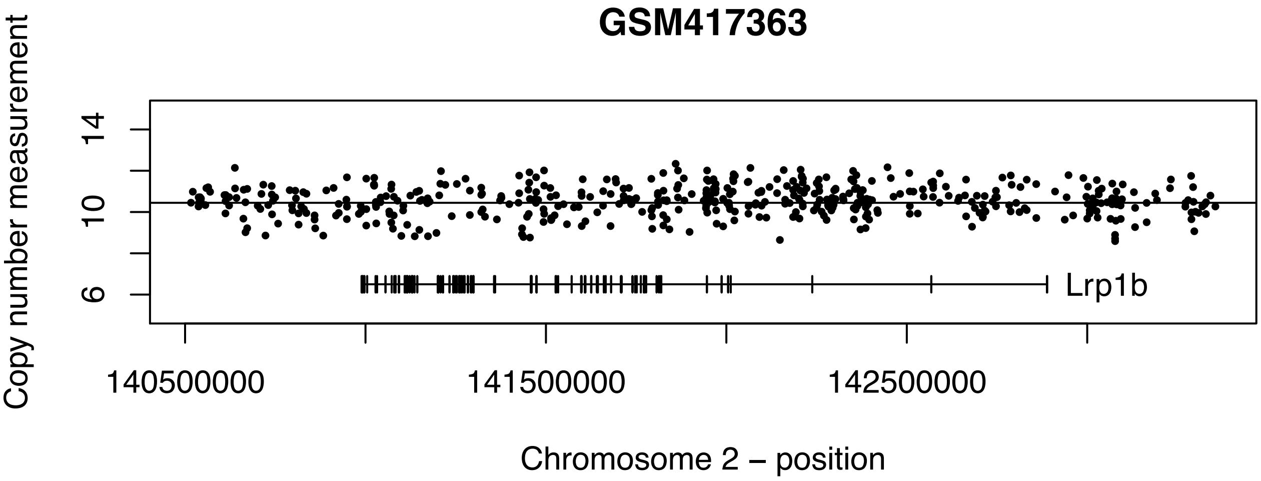
GSM417363
Copy number measurement
14
10
6
Lrp1b
140500000
141500000
142500000
Chromosome 2 – position

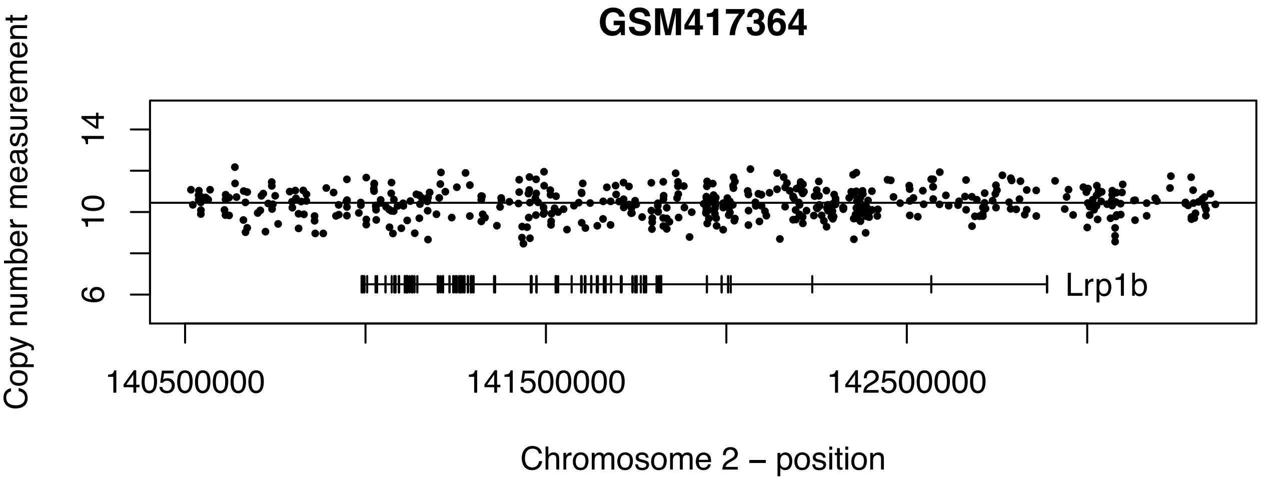
GSM417364
Copy number measurement
14
10
6
Lrp1b
140500000
141500000
142500000
Chromosome 2 – position

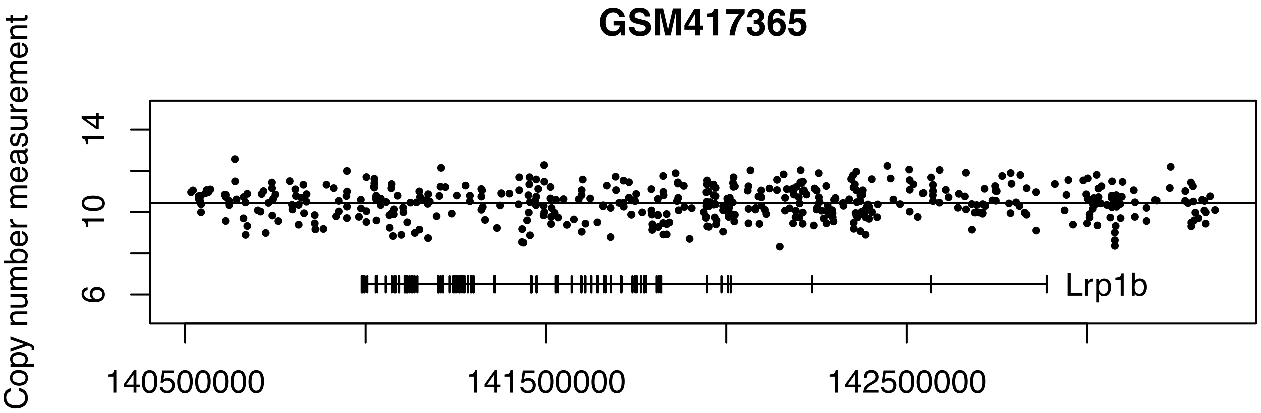

GSM417365
Copy number measurement
14
10
6
Lrp1b
140500000
141500000
142500000
Chromosome 2 – position

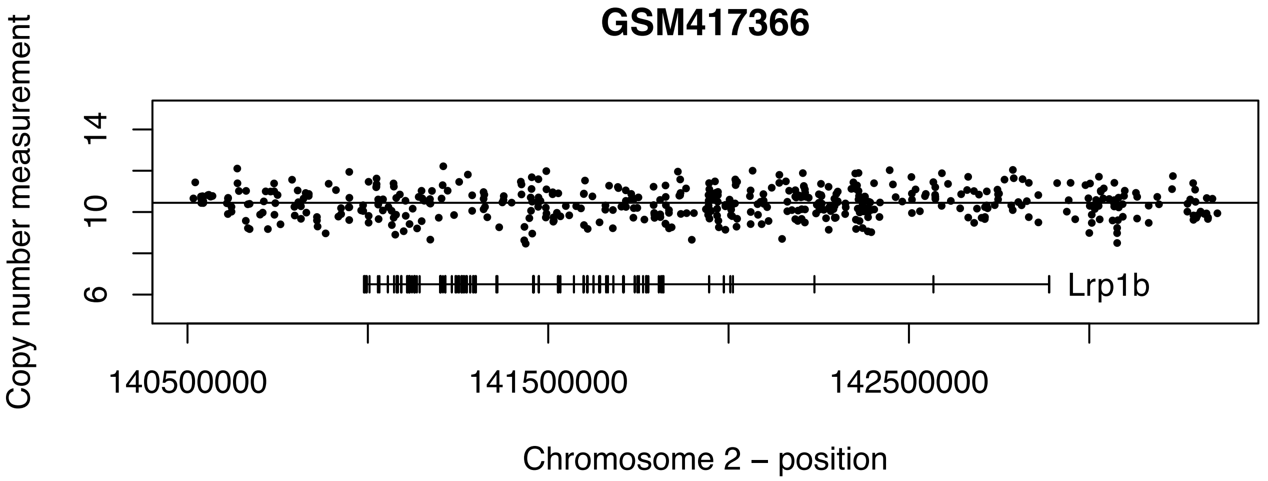

GSM417366
Copy number measurement
14
10
6
Lrp1b
140500000
141500000
142500000
Chromosome 2 – position

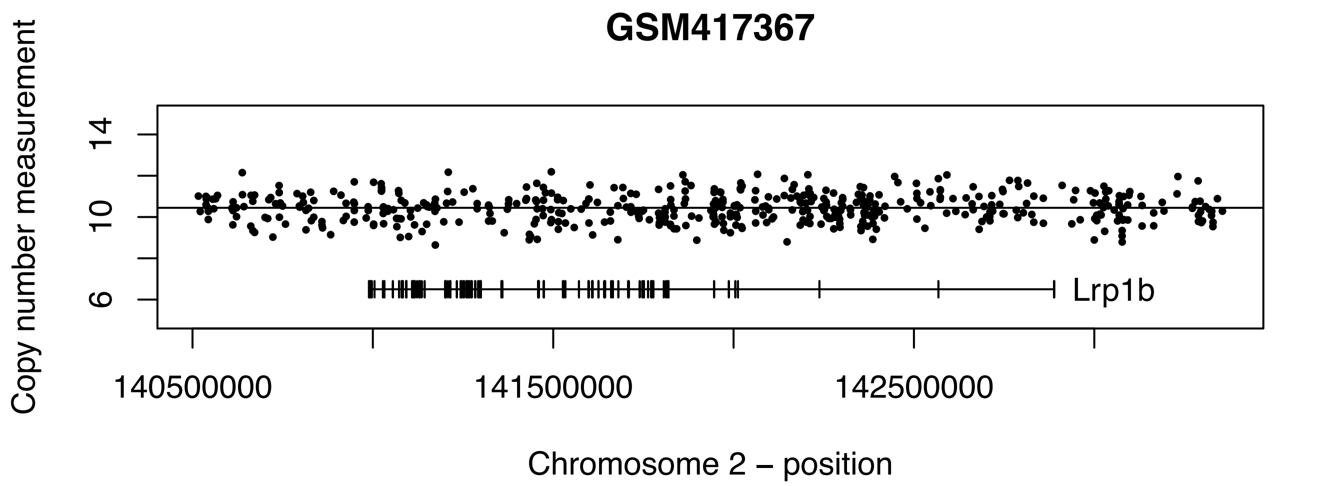

GSM417367
Copy number measurement
14
10
6
Lrp1b
140500000
141500000
142500000
Chromosome 2 – position

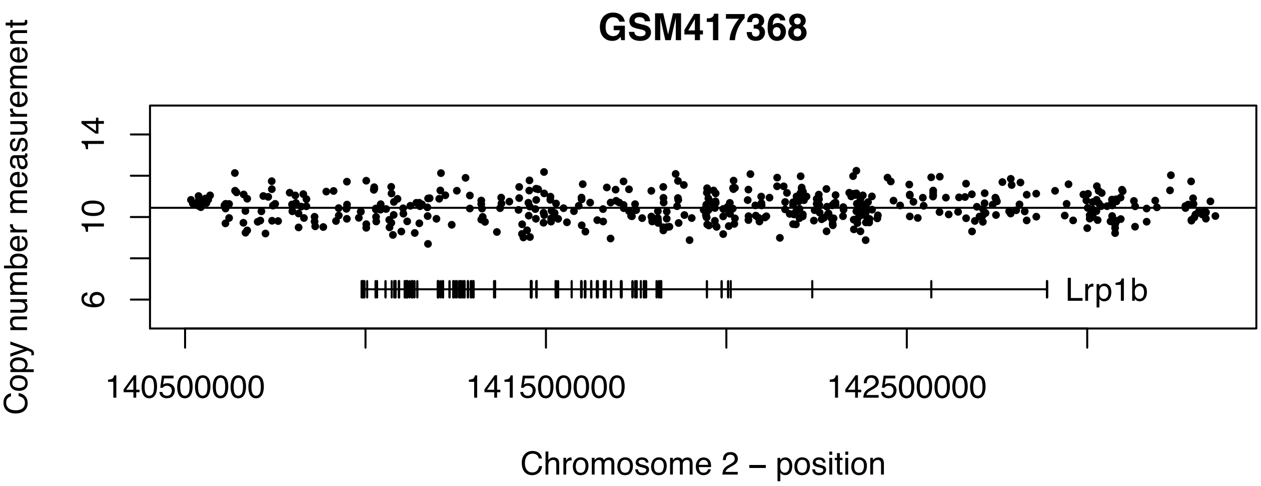

GSM417368
Copy number measurement
14
10
6
Lrp1b
140500000
141500000
142500000
Chromosome 2 – position

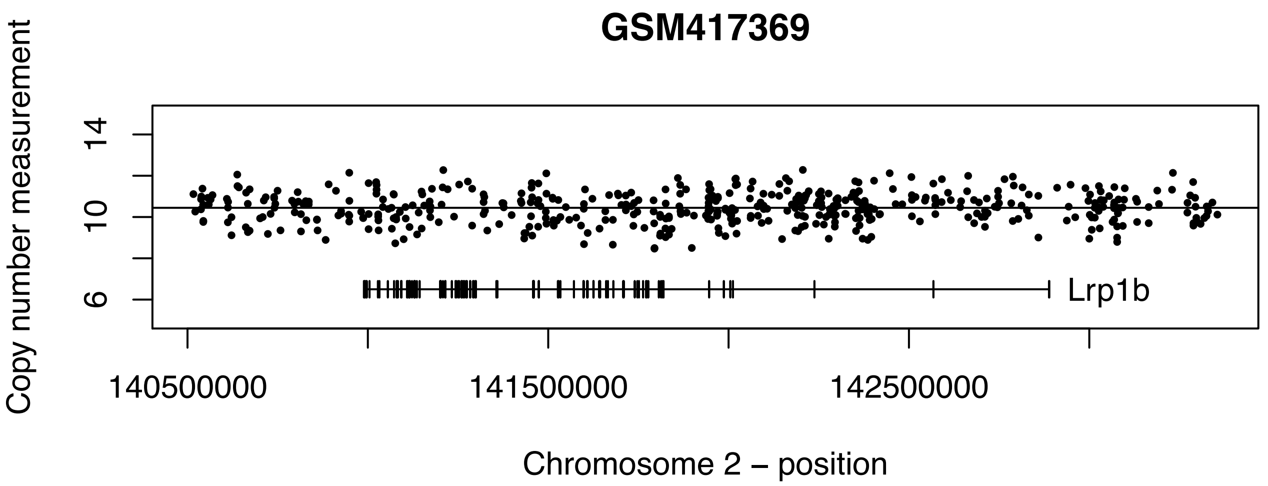

GSM417369
Copy number measurement
14
10
6
Lrp1b
140500000
141500000
142500000
Chromosome 2 – position
