# Supplementary material for: Somatic structural rearrangements in genetically engineered mouse mammary tumors
Source: Genome Biol. 2010 Oct 13;11(10):R100. doi: 10.1186/gb-2010-11-10-r100 (PMC3218656; doi:10.1186/gb-2010-11-10-r100)
Supplement: Additional file 5 — Primers used for fusion gene validation. [file gb-2010-11-10-r100-S5.DOCX]

**Additional File 5**

Primers used on cDNA for fusion gene and internally deleted gene validation

*Lrp1b:*

Lrp1b_3F CTAGCGCATGTGTCCATTTG
Lrp1b_2F AATTTCTTTGCCACGACCAT
Lrp1b_11R TAGCTTCCGCGTGAAAATCT
Lrp1b_12R CATTCCTCCTTCCAGCAGAG

*Tpd52l1-Rnf217 fusion:*

Tpd52l1_1F CGCTACCATCTGCTGCTCTG
Tpd52l1_2F GAGATGGGGATGCAGTAGGC
Rnf217_2R TCGGCCAAGTTCCAAGAAGT
Rnf217_3R TCAATTGGCAAGTGGGACAC

*Aldh8a1-6330407J23Rik fusion:*

Aldh8a1_5F AGGTCCAGCTTTGCTAACCA
Aldh8a1_6F GGGGGAGATTTGCCTTTGTA
6330407J23Rik_5R CTCTGTGGGTGTCTGCTGAG
6330407J23Rik_6R TCAGGGCAG CTTCTTCCTTA

All primers are oriented from 5’ to 3’.
